# Theoretical Study of Sesterfisherol Biosynthesis: Computational Prediction of Key Amino Acid Residue in Terpene Synthase

**DOI:** 10.1038/s41598-018-20916-x

**Published:** 2018-02-06

**Authors:** Hajime Sato, Koji Narita, Atsushi Minami, Mami Yamazaki, Chao Wang, Hironori Suemune, Shingo Nagano, Takeo Tomita, Hideaki Oikawa, Masanobu Uchiyama

**Affiliations:** 1 Graduate School of Pharmaceutical Sciences, The University of Tokyo, 7–3–1 Hongo, Bunkyo-ku, Tokyo, 113–0033 Japan; 2 Elements Chemistry Laboratory, RIKEN, and RIKEN Center for Sustainable Resource Science (Wako campus), 2–1 Hirosawa, Wako-shi, Saitama-ken, 351–0198 Japan; 3 Graduate School of Pharmaceutical Sciences, Chiba University, Chiba, 260–8675 Japan; 4 Division of Chemistry, Graduate School of Science, Hokkaido University, Sapporo, 060–0810 Japan; 5 Department of Engineering, Graduate School of Sustainability Science, Tottori University, Tottori, 680–8552 Japan; 6 Department of Chemistry and Biotechnology, Graduate School of Engineering, Tottori University, Tottori, 680–8552 Japan; 7 Biotechnology Research Center, The University of Tokyo, 1–1–1 Yayoi, Bunkyo-ku, Tokyo, 113–8657 Japan

## Abstract

The cyclization mechanisms involved in the biosynthesis of sesterterpenes are not fully understood. For example, there are two plausible reaction pathways for sesterfisherol biosynthesis, which differ in the order of ring cyclization: A-D-B/C (Path a) and A-B-C/D (Path b). It is difficult to capture intermediates of terpene cyclization, which is a complex, domino-type reaction, and so here we employed a combination of experimental and computational methods. Density functional theory calculations revealed unexpected intermediates and transition states, and implied that C–H···π interaction between a carbocation intermediate and an aromatic residue of sesterfisherol synthase (NfSS) plays a critical role, serving to accelerate the 1,2-H shift (thereby preventing triquinane carbocation formation) and to protect reactive carbocation intermediates from bases such as pyrophosphate or water in the active site. Site-directed mutagenesis of NfSS guided by docking simulations confirmed that phenylalanine F191 is a critical amino acid residue for sesterfisherol synthase, as the F191A mutant of NfSS produces novel sesterterpenes, but not sesterfisherol. Although both pathways are energetically viable, on the basis of our computational and experimental results, NfSS-mediated sesterfisherol biosynthesis appears to proceed *via* Path a. These findings may also provide new insight into the cyclization mechanisms in related sesterterpene synthases.

## Introduction

Terpenes/terpenoids comprise the largest class of natural products^1^. The biosynthesis of mono-, sesqui- and di-terpenes has been much more extensively studied than that of sesterterpenes. Since our identification of the first fungal sesterterpene synthase, ophiobolin F synthase, in 2013^2^, at least six sesterterpene synthases have been characterized using a genome mining approach^3–10^, but the cyclization mechanisms remain to be established due to the complexity of the multi-cyclic skeletons of the products and the “domino” character of the reactions.

**Figure 1:**
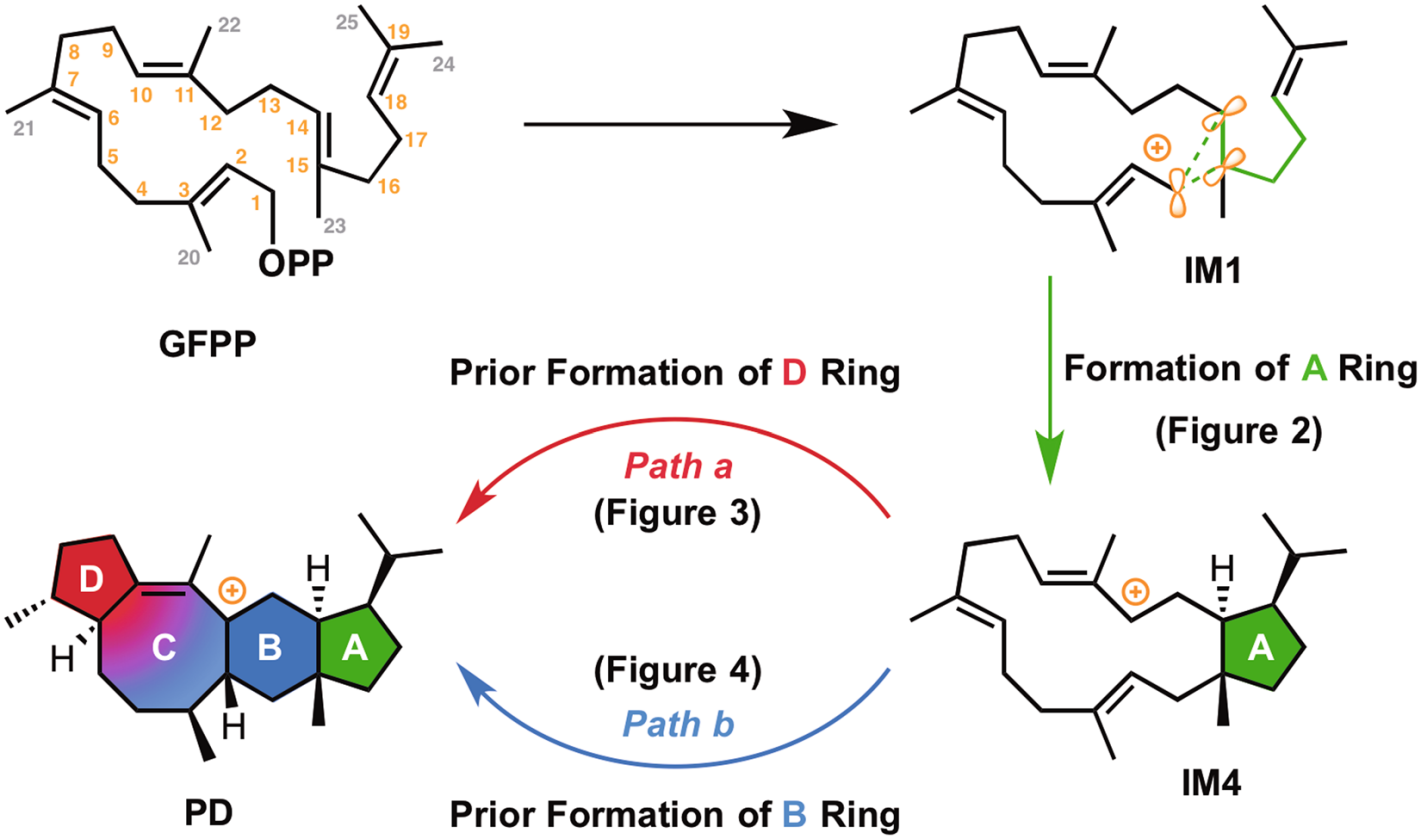
Two possible biosynthetic pathways for sesterfisherol.

We recently reported sesterfisherol synthase NfSS^3^ that is a representative enzyme of fungal sesterterpene synthase and closely related enzymes^4^. Isotopic labeling experiments with [1-^13^C,^2^H_3_]acetate and (8,8-^2^H_2_) geranylgeranyl diphosphate indicated that the ^13^C-labeling pattern of sesterfisherol (**1**) is identical to that of GFPP, and multiple hydrogen shifts take place during the cyclization process. However, the details remain unknown, in part because it is very difficult to capture the reaction intermediates of the multi-step terpene cyclization. One intriguing aspect of the NfSS-catalyzed reaction is the order of the ring-closing steps (**Path a**: A ring-D ring-B/C ring, **Path b**: A ring-B ring-C/D ring, Fig. 1), because many sesterterpenes that can plausibly be biosynthesized via pathways corresponding to either **Path a** or **Path b** have been isolated from both fungi and plants^3^.

In order to clarify this issue, we carried out density functional theory (DFT) calculations combined with the AFIR (artificial force induced reaction) method and Reaction Plus method to comprehensively unveil this biosynthetic pathway, in combination with experimental findings.

## Results

### Computed Mechanisms and Selectivity

**Figure 2:**
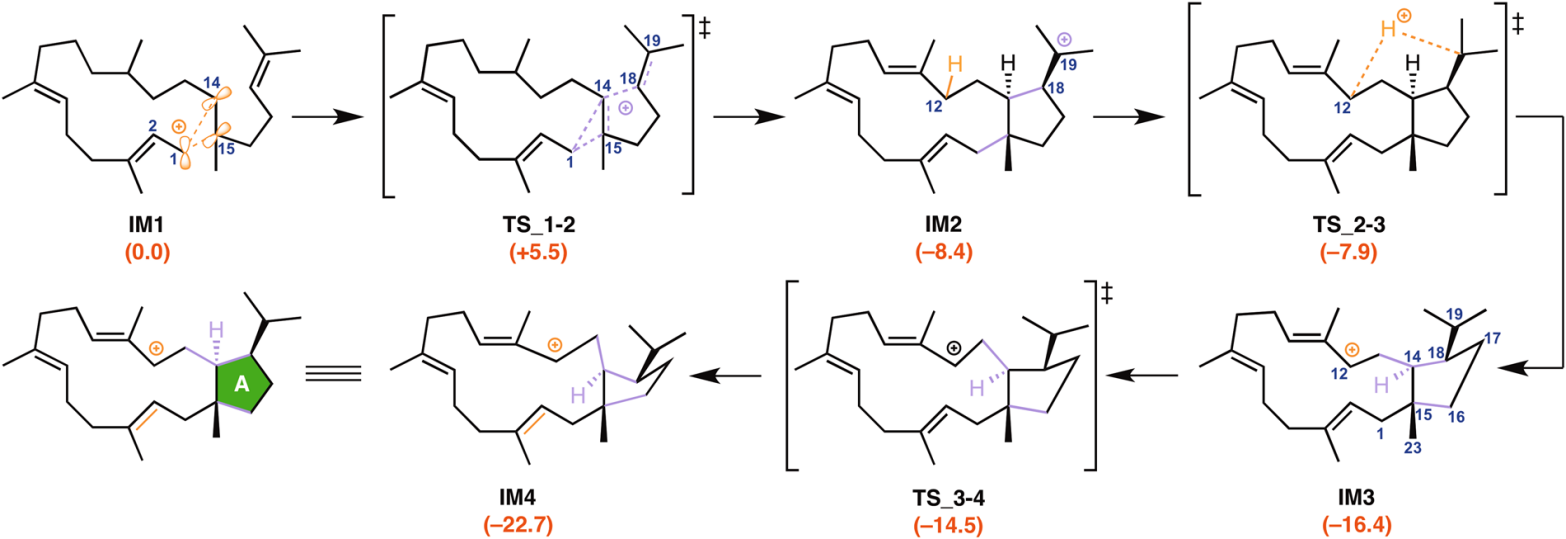
Computed reaction pathway from IM1 to the bifurcating point, IM4. IM: intermediate, TS: transition state.

**Figure 3:**
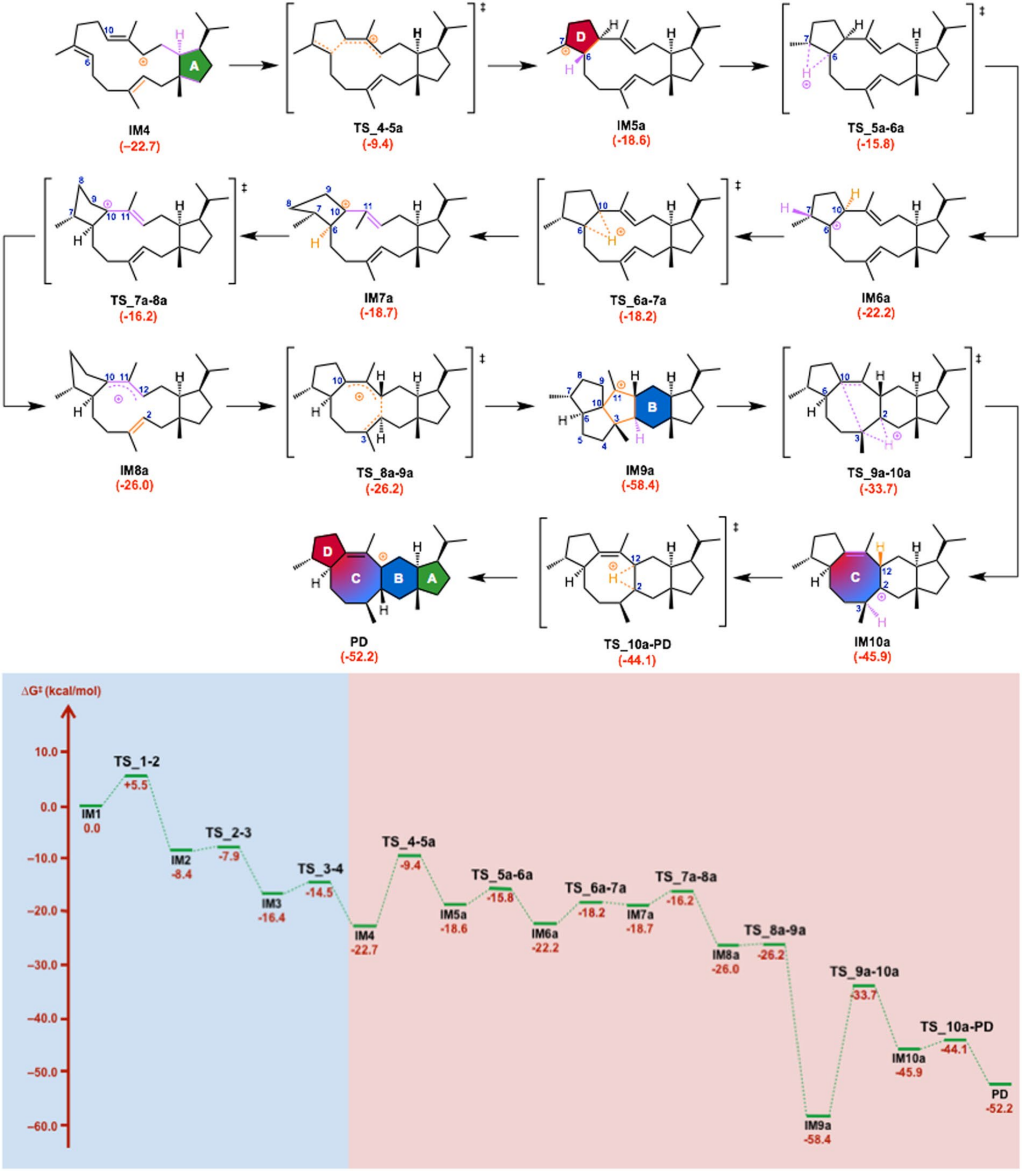
Computed reaction pathway and potential energy profile of Path a. Potential energies (kcal/mol, Gibbs free energies calculated at mPW1PW91/6–31 + G(d,p) based on M06–2X/6–31 G(d,p) geometries) relative to **IM1** are shown in red.

**Figure 4:**
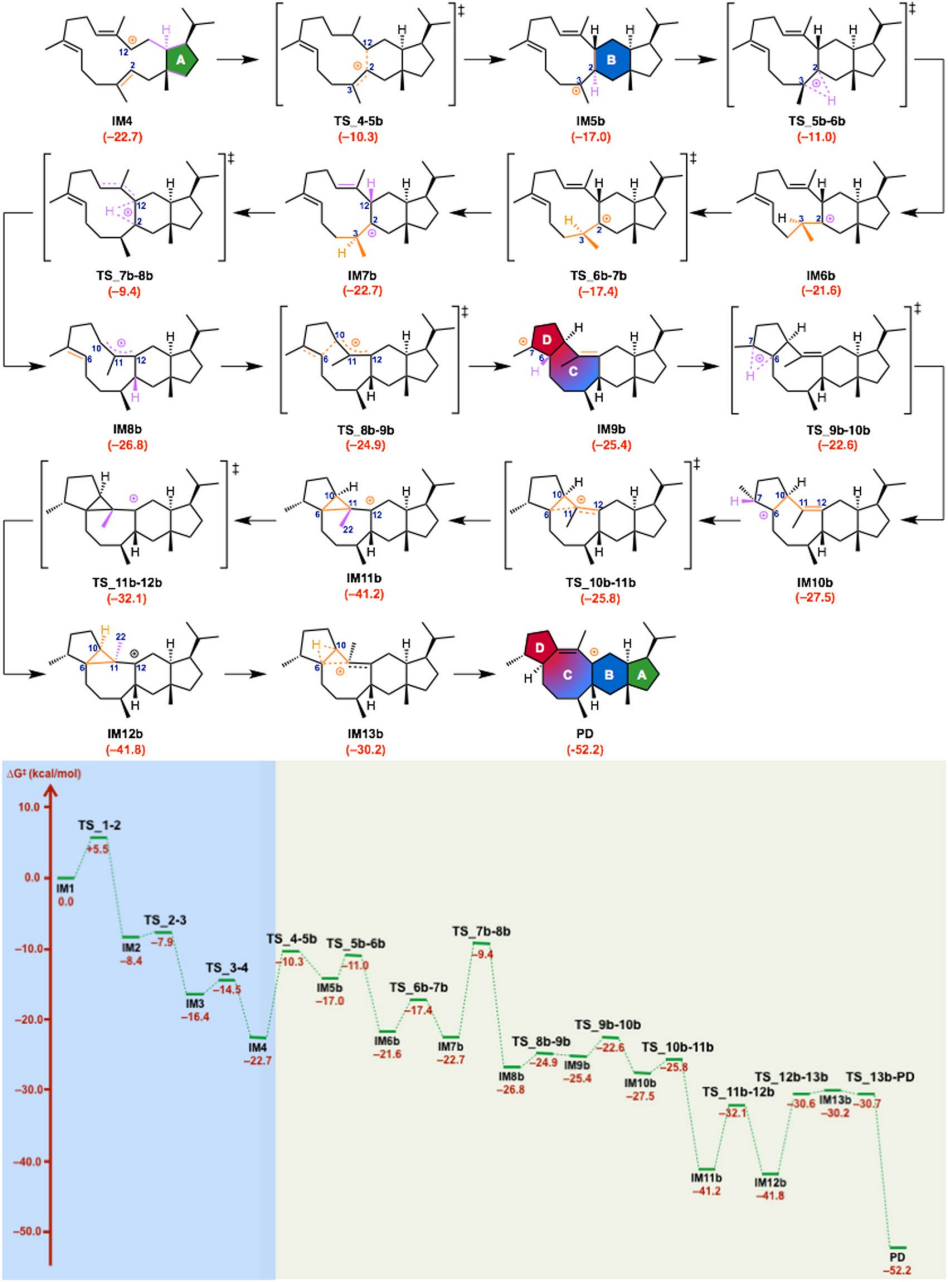
Computed reaction pathway and potential energy profile of Path b. Potential energies (kcal/mol, Gibbs free energies calculated at mPW1PW91/6–31 + G(d,p) based on M06–2X/6–31 G(d,p) geometries) relative to **IM1** are shown in red.

The computed pathways for the conversion of geranylfarnesyl diphosphate (**GFPP**) to the tetracyclic carbocation **PD** are illustrated in Fig. 1. These pathways contain more than 10 steps, and the energy diagram (Figs 2,3 and 4) suggests that they are both thermodynamically and kinetically favorable: (1) the activation barriers are all low enough for the reactions to proceed smoothly at ambient temperature, (2) the entire energy profile descends as the reactions proceed, and (3) the overall exothermicity is very large.

In the first stage of sesterfisherol biosynthesis, both pathways share exactly the same route for construction of the A ring from **IM1** to **IM4** (Fig. 2). Initially, NfSS promotes the dissociation of the pyrophosphate of **GFPP**, which yields an allylic carbocation **IM1** partially stabilized by cation-π interaction with a distal C14–C15 double bond. Cation-mediated annulation proceeds smoothly to form a bicyclic 5–15 fused-ring intermediate **IM2**, which undergoes sequential 1,5-hydrogen shift and conformational change (in **IM3**) to give a more stable allylic carbocation intermediate (**IM4)** with reasonable activation energies. **IM4** is the branch point in sesterfisherol biosynthesis. The driving force of the conformational change of **TS_3–4** is the steric repulsion among the substituent groups on the A ring. Through this reaction, the ^*i*^Pr group and C15-C1 bond both go to equatorial positions, whereas C14–C13 bond goes to the axial position (Figure S1). The next stage branches into two possible routes (**Path a** or **Path b**) according to the priority for the construction of the D or B ring.

In ***Path a*** (Fig. 3), the D ring is formed prior to the B ring. The allylic carbocation in **IM4** is partially stabilized with a distal C6–C7 double bond. Such cation-π interaction leads to smooth formation of the D ring with an activation energy of 13.3 kcal/mol, giving a tricyclic 5–12–5 fused-ring intermediate **IM5a**. Two successive hydrogen shifts (**IM5a→IM6a→IM7a**) and one conformational change (**TS_7a-8a**) afford an allylic carbocation intermediate (**IM8a**). In the **IM7a** structure, the C10–C11 bond is vertical to the D ring. The driving force of the conformational change **TS_7a-8a** is formation of the C10 carbocation plane. The D ring’s conformation is changed concomitantly (Fig. S2). The activation energy of **TS_8a-9a** is low (*∆*G^‡^ = 0.7 kcal/mol at M06–2X/6–31 G(d,p); –0.2 kcal/mol at mPW1PW91/6–31 + G(d,p)), and triquinane skeleton formation^11^ proceeds smoothly with a large stabilization (*∆*G = −32.4 kcal/mol). The final cyclization (B and C rings) then occurs with an activation energy of 24.7 kcal/mol from **IM9a** to give **IM10a** with a tetracyclic 5–6–8–5 fused-ring skeleton, followed by a sequential 1,2-hydrogen shift to give the allylic carbocation **PD**.

In ***Path b*** (Fig. 4), the B ring is formed prior to the D ring. The formation of the B ring occurs smoothly from **IM4** through a cation-π interaction with a distal C2–C3 double bond to give a tricyclic 5–6–11 fused-ring intermediate **IM5b** with an activation barrier of 12.4 kcal/mol. Then, a successive transformation involving (1) H-shift (**IM5b**→**IM6b**), (2) conformational change (**IM6b**→**IM7b**) and (3) a second H-shift (**IM7b**→**IM8b**) takes place to give the allylic carbocation intermediate **IM8b**. Unexpectedly, it was found that the C11 methyl group faces inside the 11-membered macro ring (Figure S3), and it flips during the final process in **Path b** (**IM12b**→**IM13b**). The conformational change **TS_6b-7b** is attributed to the rotation of the C3 methyl group (Figure S4). In **IM6b**, the C3 hydrogen contributes to the stabilization of the C2 carbocation through a hyper-conjugation interaction. In **IM7b**, the C3–C20 bond is perpendicular to the C2–C12 bond, which indicates that the C3 methyl group stabilizes the C2 carbocation. The 5-membered ring annulation proceeds smoothly to give a tetracyclic 5–6–8–5 fused-ring intermediate **IM9b**. Then, 1,2-hydrogen shifts occur to give homo-allylic carbocation **IM10b**, leading to the relatively more stable cyclopropylcarbinyl carbocation intermediate (**IM11b**), which is omnipresent in other terpene-forming reactions^12–15^. Aside from stereochemistry, the skeleton of **IM11b** is essentially the same as that of the intermediates of astellatene^10^ and aspterpenacid^16^, implying that they may be synthesized through **Path b**. Finally, conformational change (**IM12b**, Figs. S5), 1,2-hydrogen shift (**IM13b**) and C–C bond cleavage reaction proceed successively, leading to the final product **PD**.

### Site-Directed Mutagenesis

**Figure 5:**
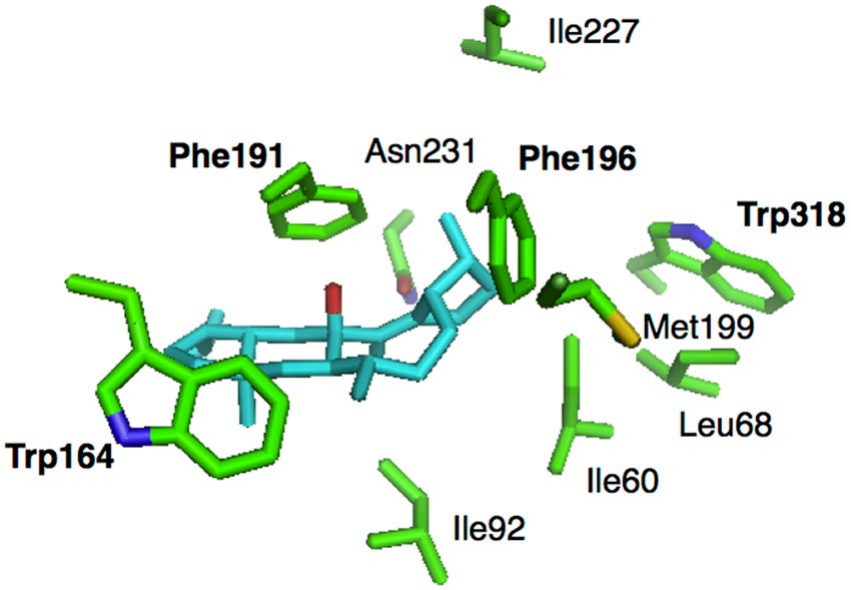
Superimposed view of the putative active site in the complex of NfSS with sesterfisherol.

**Figure 6:**
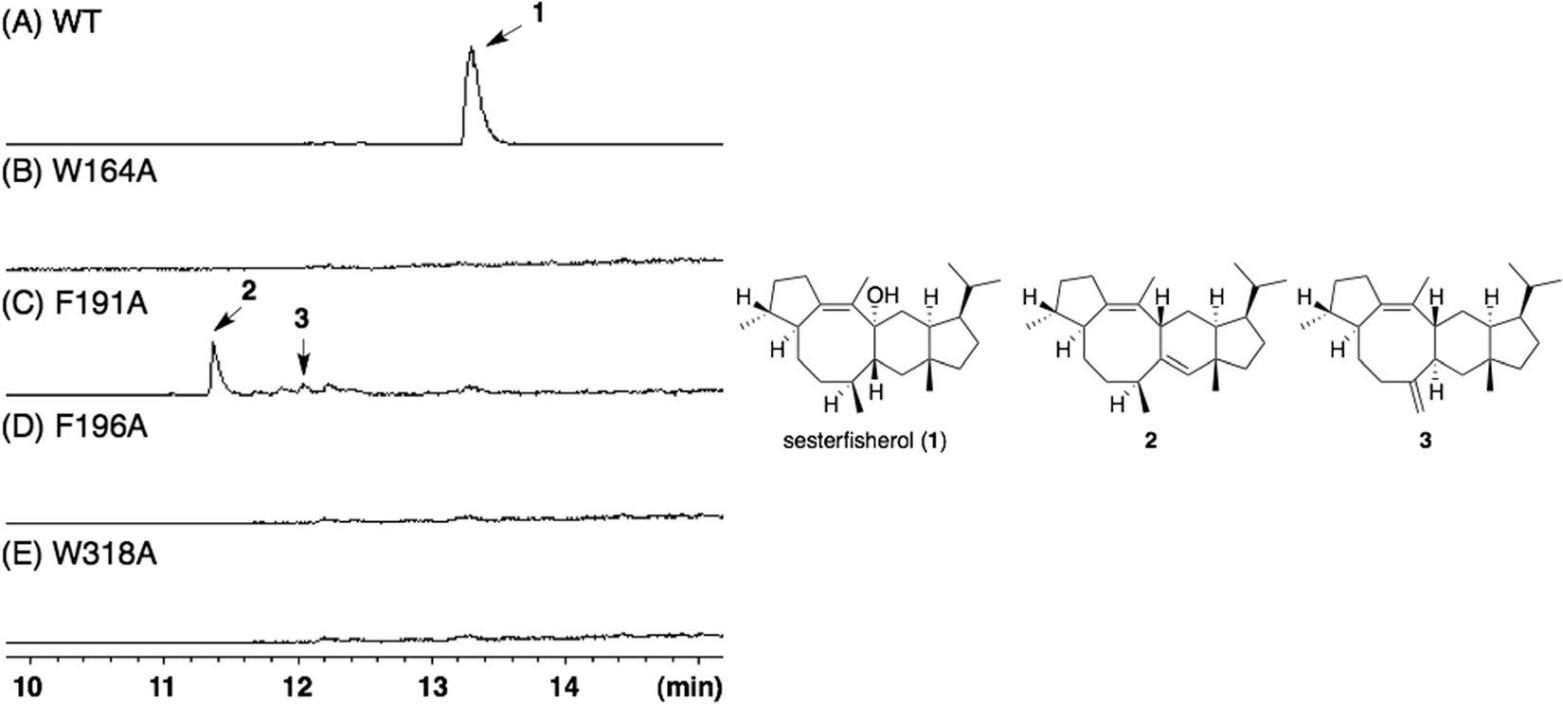
GC-MS profiles of metabolites formed by *E*. *coli* transformants: (**A**) NfSS wild type, and mutants (**B**) W164A, (**C**) F191A, (**D**) F196A, and (**E**) W318A. The chemical structures of sesterterpenes, sesterfisherol, **2**, and **3** are shown on the right side.

Having explored the branched cyclization mechanisms of NfSS (**Paths a** and **b**) by means of DFT calculations, we next experimentally investigated the NfSS-catalyzed cyclization pathway by means of site-directed mutagenesis, guided by docking simulations. Considering that the terpene synthase domain of NfSS shows moderate homology with that of PaFS^17^ (identity/similarity: 24%/44%), we built a 3D molecular model of NfSS using the coordinates from the reported crystal structure of PaFS, and conducted a docking simulation of NfSS with **1**. The results indicated involvement of several amino acid residues, such as I60, L68, I92, W164, F191, F196, and W318 (Figs 5, S6 and S7), in the putative active site. Among them, we focused on aromatic amino acid residues, because cation-π and C–H···π interactions are proposed to be important in the cyclizations catalysed by several terpene cyclases. Four plasmids were constructed and used for transformation of *E*. *coli* to afford mutants, EC-W164A, EC-F191A, EC-F196A, and EC-W318A. While EC-W164A, EC-F196A, and EC-W318A gave no product (Fig. 6), TLC and GC-MS analyses of the metabolites generated by EC-F191A showed the formation of two metabolites, **2** (0.18 mg/L) and **3** (0.04 mg/L) (Figures S8, and S9). HR-MS analysis revealed that the molecular formulae of **2** and **3** were C_25_H_40_. ^1^H- and ^13^C-NMR spectra of **2** showed one olefinic proton (δ_H_ = 5.64 (s)) and four olefinic carbons (δ_C_ = 129.1, 133.4, 139.9, and 144.6). Extensive 2D NMR analysis including HSQC, HMBC, COSY, and NOESY enabled us to determine the structure of **2** as shown in Figure S10. Comparison of the ^1^H-NMR spectra of **1** and **3** indicated that one of the methyl groups of **1** was converted into an exo-olefin (δ_H_ = 4.90 and 4.98). The structure of **3** was determined by extensive NMR analysis as shown in Figure S10. The carbon skeletons of **2** and **3** correspond to those of proposed intermediates in ***Path a***. This result strongly supports the idea that NfSS mediates biosynthesis of sesterfisherol via ***Path a***.

### C-H···π interaction

**Figure 7:**
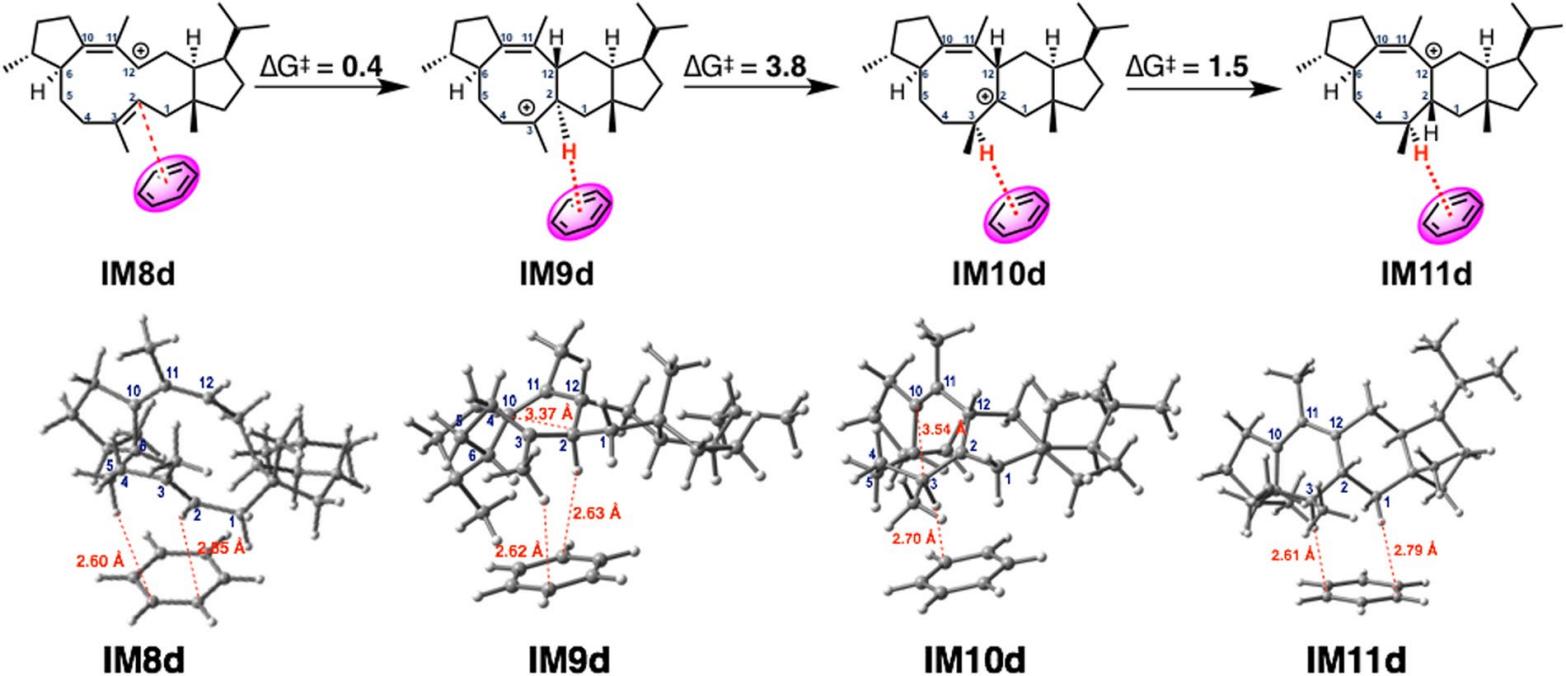
Computed reaction pathway from IM8d to IM11d, and the 3D structures.

**Path a** has a deep valley leading to the triquinane-type intermediate (**IM9a**). Since an activation energy of 25 kcal/mol is a borderline barrier from the viewpoint of whether a reaction can takes place spontaneously at ambient temperature inside the enzyme, we speculated that there might be an alternative catalytic mechanism for this step. Tantillo & Hong have uncovered several terpene biosynthetic processes by using computational methods, and suggested that C–H···π interaction plays an important role in accelerating hydrogen shifts in some cases^17,18^. Since F191 afforded cyclized products, **2** and **3**, which might be related to intermediate **IM10a**, we applied the theozyme approach in the present computational analysis. The results showed that C–H···π interaction can not only accelerate 1,2-H shifts, but also block triquinane skeleton formation (Fig. 7). The second intermediate **IM9d** is a C–H···π complex that undergoes two successive 1,2-H shifts *via* **TS_9d-10d** and **TS_10d-11d**^19^. These transition state structures are stabilized by the C–H···π interaction, and the activation barriers become quite small.

## Discussion

**Figure 8:**
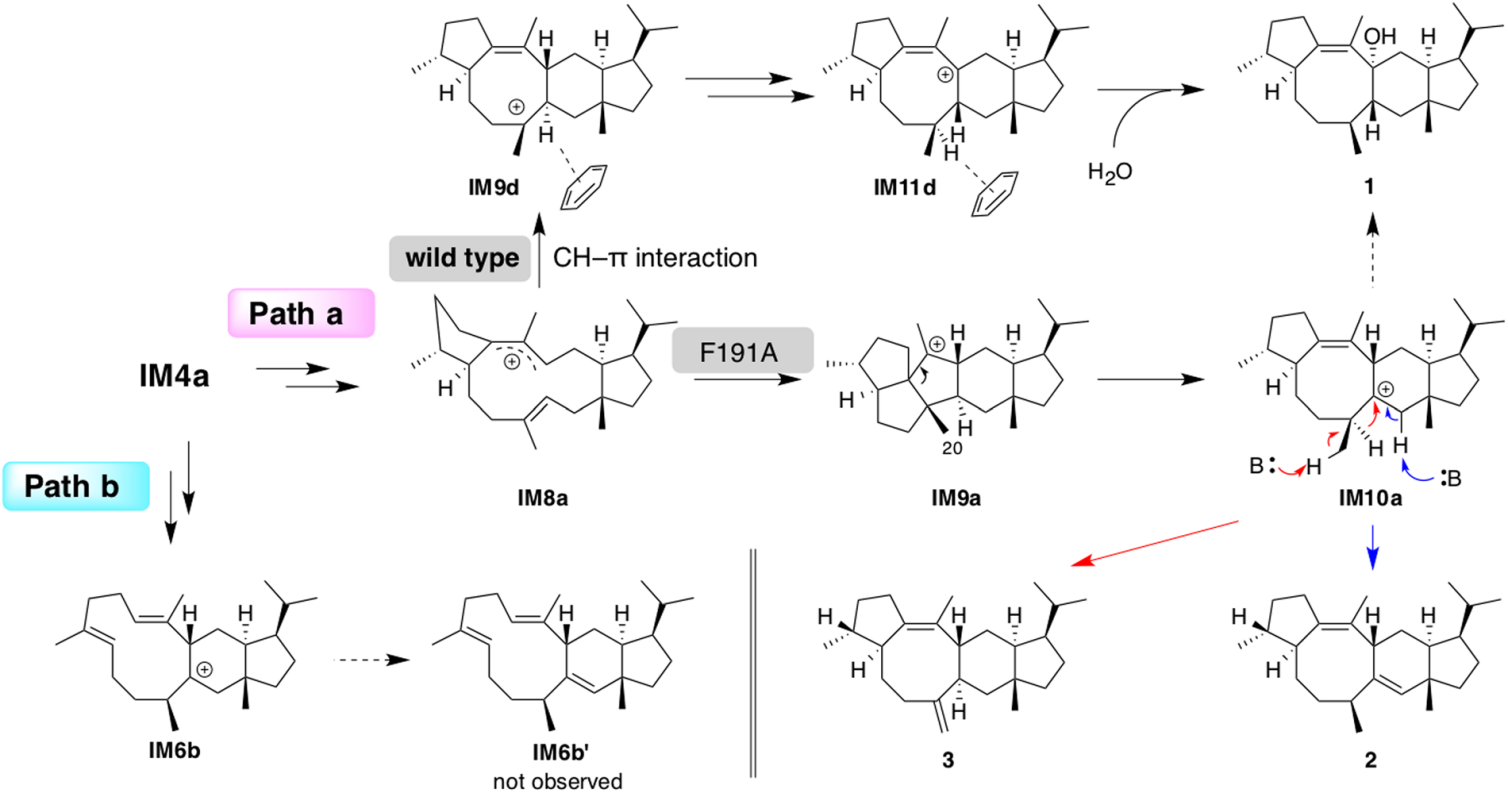
Proposed reaction mechanisms leading to products 2 and 3.

Two alternative cyclization pathways mediated by NfSS (**Paths a** and **b**) for the formation of **1** were considered plausible on the basis of DFT calculations. The differences are in the order of cyclizations; A-D-B/C in **Path a** and A-B-C/D in **Path b**. To investigate the cyclization pathway experimentally, we employed mutational analysis of amino acid residues identified as potentially important in docking simulations. Among four mutants, F191A afforded two compounds, **2** and **3**, which seem to be produced by deprotonation at the C1/C20 positions of **IM10a** in **Path a** (Fig. 8). If we assume that the orientation of the intermediates is retained during the cyclization, diphosphate dissociated from **GFPP** or water in the active site could serve as a base to give **2** and **3**. On the other hand, if the enzyme reaction employs **Path b**, F191A should generate tricyclic derivatives of **IM6b** or **IM7b** lacking a C6–C10 bond (Fig. 4, **IM6b’**). Therefore, the NfSS-catalyzed cyclization most likely proceeds via **Path a**, not **Path b**.

**Path a** contains one transformation, from **IM9a** to **IM10a**, with a moderately high activation process (*∆*G^‡^ = 24.7 kcal/mol), which is a borderline energy barrier for the process to occur at ambient temperature. Intriguingly, though, when involvement of C–H···π interaction was considered, energetically preferred transformations from **IM8d** to **IM11d** emerged as an alternative cyclization mechanism (Fig. 7). In this pathway, C2–C12 cyclization and two rounds of 1,2-H shifts occur successively to afford **IM11d**, leading to **1** (Fig. 8). Therefore, wild-type NfSS may employ this modified pathway. The absence of production of intermediates, such as **2** and **3**, supports this biosynthetic hypothesis. In contrast, F191A NfSS produced **2** as a major product, indicating the involvement of **IM10a** found in **Path a**. The deprotonation to give **2** and **3** might take place faster than the 1,2-H shift *via* **TS_10a-PD**, because **1** was not produced by the F191A mutant. Even though the **IM9d**-like carbocation can be formed along the intrinsic reaction coordinate (IRC^20–24^) path from **TS_9a-10a** to **IM10a**, the formation of **IM10a** proceeds predominantly, because the **IM9d-**like carbocation is a transient structure leading to **IM10a** without an energy barrier. If the **IM9d**-like carbocation was a stable intermediate, the F191A mutant should have given compound **3** as a major product. Overall, based on these considerations, we propose that F191 is a critical residue that serves 1) to accelerate the 1,2-H shift, thereby preventing triquinane carbocation intermediate formation, and 2) to block deprotonation at the C1/C20 positions. Since the present docking simulations with **1** cannot afford conclusive evidence regarding the putative C–H···π interaction, involvement of other amino acid residues cannot be ruled out. Solving the crystal structures of NfSS complexes with both a substrate analog and **1** would facilitate detailed understanding of the cyclization mechanisms.

Cation intermediates proposed in **Path a** have the same core structure as various fungal and plant sesterterpenes, such as nitiol^25^ and variculanol^26^ (Figure S11). Similarly, some natural sesterterpenes, such as astellatene^10^, thalianatriene^27^, and aspterpenacids^28^, could be produced from the cation intermediates found in **Path b** (Figure S11). The structural resemblances of natural sesterterpenes to these proposed cationic intermediates provide circumstantial support for the idea that both pathways are available in sesterterpene biosynthesis. The switching mechanism at the branch point **IM4** is very interesting, because there is only a tiny difference of the activation energy from **IM4** to **IM5a**/**IM5b** (**TS_4–5a**: 13.3 kcal/mol, **TS_4–5b**: 12.4 kcal/mol).

Recently, some plant sesterterpene synthases have been functionally characterized. As expected, these terpene synthases also follow either **Path a** or **Path b** to afford sesterfisherol-related plant sesterterpenes^10^. This conclusion was further supported by DFT calculations for the formation of plant sesterterpene astellatene. Among plant sesterterpene synthases, AtTPS19 from *Arabidopsis thaliana* is unique in that it produces four sesterterpenes, whose structures are closely related to the carbocation intermediates proposed in **Path a** and **Path b**. These results suggested that rational protein engineering of terpene synthases would facilitate convenient enzymatic syntheses of those sesterterpenes.

In this paper, we have elucidated the ring construction sequence in NfSS-catalyzed cyclization by employing a combination of of DFT calculations and mutational analysis. In addition, a theozyme approach based on the mutagenesis studies implied the importance of C–H···π interaction during the cyclization. The hydrocarbon skeletons of the proposed intermediates also allowed us to propose plausible cyclization mechanisms for several sesterterpenes found in fungi and plants. We consider that DFT calculations are a powerful tool for understanding the cyclization mechanisms of terpenoids, because some of the reaction intermediates (**IM9a**, **IM8b-IM12b**) both in **Path a** and **Path b** would have been hard to be predict in the absence of DFT calculation, especially as regards their regio- and stereo-selectivities.

## Methods

### General

All commercial reagents were used as received. Column chromatography was carried out on 60 N silica gel (Kanto Chemicals). Optical rotations were recorded on JASCO P-2200 digital polarimeter. ^1^H- and ^13^C-NMR spectra were recorded on Bruker DRX-500 or Bruker AMX-500 spectrometer (500 MHz for ^1^H-NMR and 125 MHz for ^13^C-NMR). NMR spectra were recorded in C_6_D_6_ (99.5 atom% enriched, Kanto). ^1^H chemical shifts were reported in δ value based on residual benzene (7.15 ppm) as a reference. ^13^C chemical shifts were reported in δ value based on benzene (128.0 ppm) as a reference. Data are reported as follows: chemical shift, multiplicity (s = singlet, d = doublet, t = triplet, q = quartet, m = multiplet, br = broad), coupling constant (Hz), and integration. GC-MS analyses were conducted with MS-2010 (Shimadzu). Mass spectra were obtained with a JEOL JMS-T100GCV (EI mode).

Oligonucleotides for polymerase chain reactions (PCRs) were purchased from Hokkaido System Science Co., Ltd. PCRs were performed with a BioRad S1000 thermal cycler.

### Computational details

All DFT calculations were carried out with the Gaussian 09 program^29^. Geometry optimizations were performed in the gas phase at the M06–2X/6–31 G(d,p) level^30^, without any symmetry restrictions. Vibrational frequency calculations at the same level of theory were performed to verify that a local minimum has no imaginary frequency and each TS has only a single imaginary frequency. Intrinsic reaction coordinate calculations^20–24^ for all TSs were performed with GRRM11^31^ and/or Reaction Plus programs^32–34^ based on Gaussian 09. Single point energies on M06–2X/6–31 G(d,p) geometries were computed with the mPW1PW91/6–31 + G(d,p) method^35,36^. This level of theory has been applied previously to a variety of terpene-forming carbocation reactions^12,16,37–39^. Gibbs free energy was used as the basis for discussion in this study.

### Docking simulations

A homology model of NfSS was constructed with Discovery studio by using the cyclization domain of fusicoccadiene synthase from *Phomopsis amygdali* (PDB ID: 5ERM) as a template. The structures of the final products obtained from DFT calculations were used for docking simulations. Docking between NfSS and an intermediate/product was performed using the AUTODOCK Vina program. Side chains of ten residues lining the substrate-binding pocket, I60, L68, I92, W164, F191, F196, M199, I227, N231, and W318, were treated as flexible. The search grid box 40 × 40 × 40 Å^3^ was placed to include the substrate-binding site. The exhaustiveness was set at 200 and default options were used for the remaining parameters.

### Construction of Plasmids

Mutations were introduced into a previously constructed pMalc4E-*NfSS* by PCR using respective primers described in Table S1 according to the manufacturer’s protocol for the PrimeSTAR Mutagenesis Basal Kit (Takara). These plasmids were separately introduced into *E*. *coli* BL21-Gold(DE3) for overexpression. The transformants were grown at 37 °C to an OD_600_ of ~0.6 in 500 mL flask. After cooling at 4 °C, isopropyl β-D-thiogalactopyranoside (0.1 mM) was added to the culture. The cells were incubated at 16 °C for 17 h, harvested by centrifugation at 4000 rpm, and then soaked with acetone. After filtration, the filtrate was concentrated in vacuo and the residue was extracted with EtOAc. The combined organic layers were concentrated in vacuo to afford crude extracts.

### Analysis of metabolites from *E*. *coli* transformants

After partial purification of the crude extracts by silica gel column chromatography (hexane), the metabolites were analyzed by GC-MS with a DB-1ms capillary column (0.32 mm × 30 m, 0.25 μm film thickness; J&W Scientific) under the following conditions. The sample was injected into the column at 100 °C in the splitless mode. After a 3 min isothermal hold at 100 °C, the column temperature was increased at 14 °C min^−1^ to 268 °C, with a 4 min isothermal hold at 268 °C. The flow rate of helium carrier gas was 0.66 mL min^−1^.

The crude extracts of EC-*F191A* were partially purified by silica gel column chromatography (hexane). Further purification by HPLC with a Wakopak^®^ Navi C18–5 (ϕ10 × 250 mm) under the following conditions (λ = 210 nm, 100% acetonitrile at a flow rate of 1.0 mL/min) gave **2** (2.1 mg from 11.2 L of LB medium) and **3** (0.5 mg from 11.2 L of LB medium).

**2**: [α]_D_^25^ + 78° (*c* 0.75, CHCl_3_); EI-HR-MS: calcd. for C_25_H_40_ [M]^+^: 340.3130, found: 340.3123. ^1^H NMR and ^13^C NMR data are summarized in Table S2.

**3**: [α]_D_^25^ + 9° (*c* 0.33, CHCl_3_); EI-HR-MS: calcd. for C_25_H_40_ [M]^+^: 340.3130, found: 340.3122. ^1^H NMR and ^13^C NMR data are summarized in Table S2.

## Electronic supplementary material

Supplementary Information
