# Supplementary material for: Theoretical Study of Sesterfisherol Biosynthesis: Computational Prediction of Key Amino Acid Residue in Terpene Synthase
Source: Sci Rep. 2018 Feb 6;8:2473. doi: 10.1038/s41598-018-20916-x (PMC5802712; doi:10.1038/s41598-018-20916-x)
Supplement: Supplementary file 1 — Supplementary Information [file 41598_2018_20916_MOESM1_ESM.doc]

**Supporting Information**

**Theoretical Study of Sesterfisherol Biosynthesis:**

**Computational Prediction of Key Amino Acid Residue in Terpene Synthase**

Hajime Sato^1,2,3^,*, Koji Narita^4^, Atsushi Minami^4^, Mami Yamazaki^3^, Chao Wang^1,2^, Hironori Suemune^5^, Shingo Nagano^6^, Takeo Tomita^7^, Hideaki Oikawa^4^, and Masanobu Uchiyama^1,2^,*

^1^ Graduate School of Pharmaceutical Sciences, The University of Tokyo, 7-3-1 Hongo, Bunkyo-ku, Tokyo 113-0033, Japan.

2 Elements Chemistry Laboratory, RIKEN, and RIKEN Center for Sustainable Resource Science (Wako campus), 2-1 Hirosawa, Wako-shi, Saitama-ken 351-0198, Japan.

3 Graduate School of Pharmaceutical Sciences, Chiba University, Chiba 260-8675, Japan.

^4^ Division of Chemistry, Graduate School of Science, Hokkaido University, Sapporo 060-0810, Japan.

^5^ Department of Engineering, Graduate School of Sustainability Science, Tottori University, Tottori 680-8552, Japan.

^6^ Department of Chemistry and Biotechnology, Graduate School of Engineering, Tottori University, Tottori 680-8552, Japan.

^7^ Biotechnology Research Center, The University of Tokyo, 1-1-1 Yayoi, Bunkyo-ku, Tokyo 113-8657, Japan.

**Table of Contents**

Figures

Figure S1: Three-dimensional representation of IM3, TS_3-4 and IM4 Page S2

Figure S2: Three-dimensional representation of IM7a, TS_7a-8a and IM8a Page S2

Figure S3: Three-dimensional representation of IM8b, IM9b and IM10b Page S3

Figure S4: Three-dimensional representation of IM6b, TS_6b-7b and IM7b Page S3

Figure S5: Three-dimensional representation of IM11b, TS_11b-12b and IM12bPage S3

Figure S6: Multiple sequence alignment Page S4

Figure S7: Homology modeling of NfSS in complex with 1 Page S4

Figure S8: GC-MS profiles of sesterterpenes Page S5

Figure S9: MS spectra of sesterterpenes Page S5

Figure S10: Summary of 2D-NMR experiments Page S6

Figure S11: Structures of sesterterpenes Page S7

Tables

Table S1: Oligonucleotides used for construction of expression plasmids Page S8

Table S2: NMR spectral data of sesterterpenes Page S9

Table S3: Energy profiles Page S10

Table S4: Cartesian coordinates Page S11

**NMR spectra** Page S40

**Figure S1.** Three-dimensional representation of **IM3**, **TS_3-4** and **IM4**.


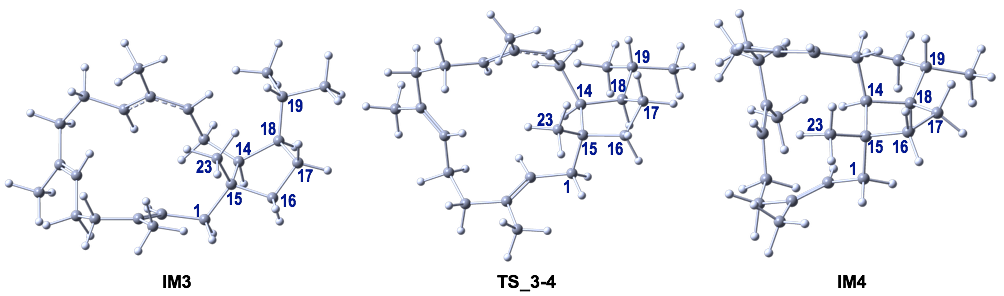


**Figure S2.** Three-dimensional representation of **IM7a**, **TS_7a-8a** and **IM8a**.


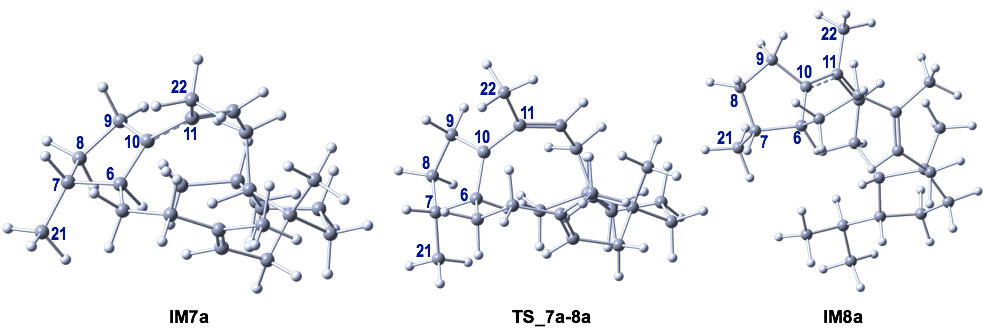


**Figure S3.** Three-dimensional representation of **IM8b**, **IM9b** and **IM10b**.


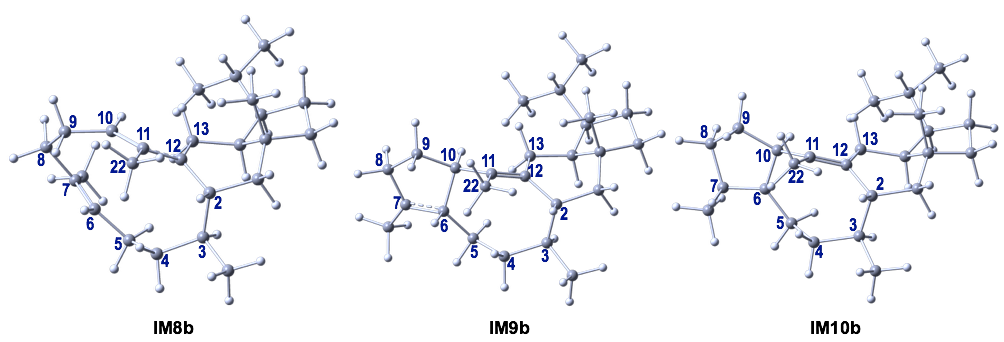


**Figure S4.** Three-dimensional representation of **IM6b**, **TS_6b-7b** and **IM7b**.


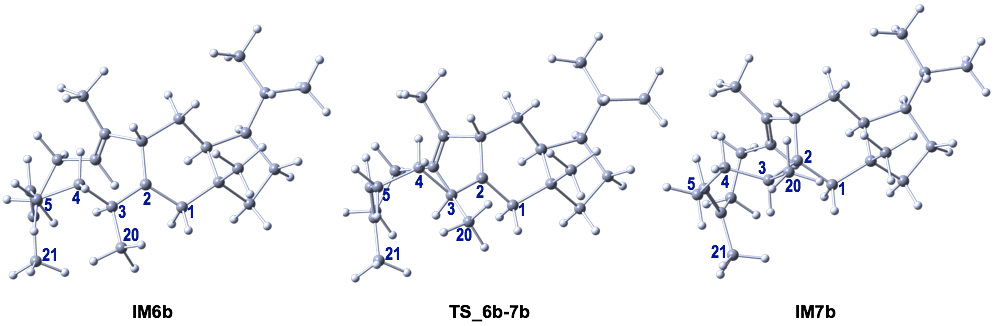


**Figure S5.** Three-dimensional representation of **IM11b**, **TS_11b-12b** and **IM12b**.


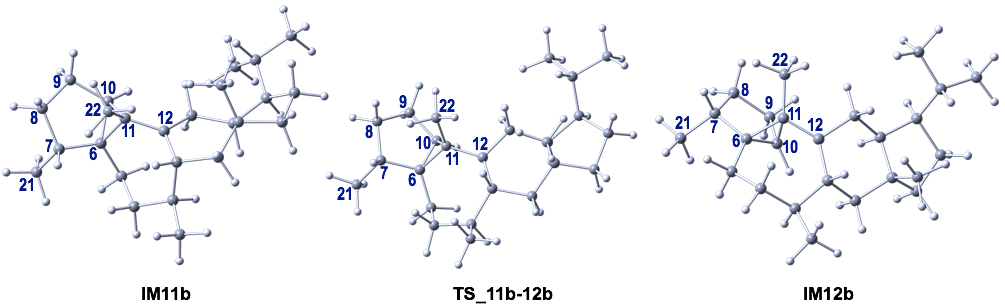


**Figure S6.** Multiple sequence alignment of functionally characterized sesterterpene synthases, fusicoccadiene synthase (PaFS;A2PZA5), phomopsene synthase (PaPS;AB254159), ophiobolin F synthase (AcOS;A1C8C3), and sesterfisherol synthase (NfSS;XP_001258098).

**Figure S7.** Superimposition of the structural model of NfSS (green) and PaFS (orange).


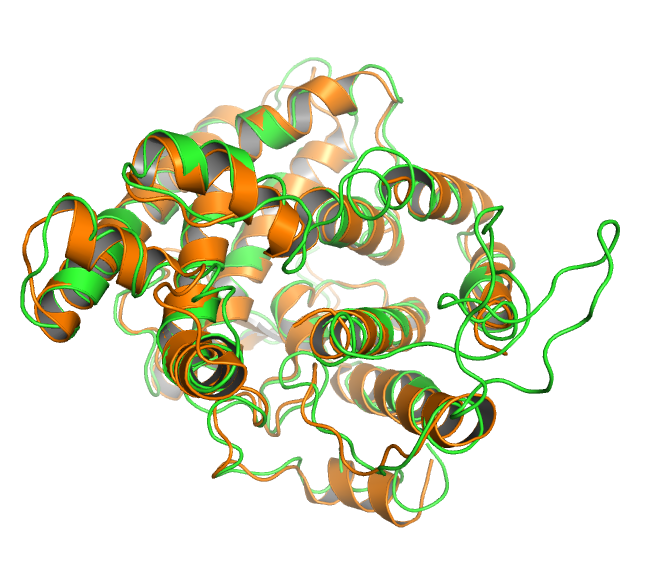


**Figure S8.** GC-MS profiles of (A) **2**, (B) **3**, and (C) **1**.

**Figure S9.** MS spectra of (A) **2**, (B) **3**, and (C) **1**.

**Figure S10.** Summary of 2D-NMR experiments: (A) **2** and (B) **3**.

**Figure S11.** Structures of sesterterpenes. Abbreviations F and P in parentheses indicate origin (F: fungi and P: plant).

**Table S1.** Oligonucleotides used for construction of expression plasmids.

| Insert | Sequence 5’-3’ |
| --- | --- |
| *NfSS* (W318A) | F: CACTTT**GCC**GCCAGCTGCTCTCCCCGT |
|  | R: GCTGGC**GGC**AAAGTGAGTACCAGCCAC |
| *NfSS* (F196A) | F: AGGGCA**GCT**TGGCCGATGGTGGAGTTT |
|  | R: CGGCCA**AGC**TGCCCTGATGCCGAAATC |
| *NfSS* (F191A) | F: GATGAT**GCA**GGCATCAGGGCATTCTGG |
|  | R: GATGCC**TGC**ATCATCCCTTCTGTAGGC |
| *NfSS* (W164A) | F: AAAGGA**GCA**TTGTCCGTTGCAGGAGGG |
|  | R: GGACAA**TGC**TCCTTTGTTATACATGTC |

**Table S2.** NMR spectral data of **2** and **3**.

**Table S3. Energy Profiles (a.u.) of Path a, Path b and theozyme calculation.**

| **Path a** | |  | **Path b** | |
| --- | --- | --- | --- | --- |
| IM1 | -976.320380 |  | IM1 | -976.320380 |
| TS_1-2 | -976.311647 |  | TS_1-2 | -976.311647 |
| IM2 | -976.333806 |  | IM2 | -976.333806 |
| TS_2-3 | -976.333001 |  | TS_2-3 | -976.333001 |
| IM3 | -976.346591 |  | IM3 | -976.346591 |
| TS_3-4 | -976.343509 |  | TS_3-4 | -976.343509 |
| IM4 | -976.356561 |  | IM4 | -976.356561 |
| TS_4-5a | -976.335396 |  | TS_4-5b | -976.336873 |
| IM5a | -976.350058 |  | IM5b | -976.347467 |
| TS_5a-6a | -976.345590 |  | TS_5b-6b | -976.337907 |
| IM6a | -976.355684 |  | IM6b | -976.354839 |
| TS_6a-7a | -976.349436 |  | TS_6b-7b | -976.348147 |
| IM7a | -976.350230 |  | IM7b | -976.356526 |
| TS_7a-8a | -976.346157 |  | TS_7b-8b | -976.335368 |
| IM8a | -976.361838 |  | IM8b | -976.363065 |
| TS_8a-9a | -976.362055 |  | TS_8b-9b | -976.360090 |
| IM9a | -976.413480 |  | IM9b | -976.360849 |
| TS_9a-10a | -976.374124 |  | TS_9b-10b | -976.356336 |
| IM10a | -976.393544 |  | IM10b | -976.364159 |
| TS_10a-PD | -976.390684 |  | TS_10b-11b | -976.361424 |
| PD | -976.403609 |  | IM11b | -976.386076 |
|  |  |  | TS_11b-12b | -976.371588 |
|  |  |  | IM12b | -976.387015 |
|  |  |  | TS_12b-13b | -976.369074 |
|  |  |  | IM13b | -976.368454 |
|  |  |  | TS_13b-PD | -976.369224 |
|  |  |  | PD | -976.403565 |

| **Theozyme** | |
| --- | --- |
| TS_8d-9d-R | -1208.473627 |
| TS_8d-9d_TS | -1208.472952 |
| TS_8d-9d-F | -1208.496629 |
| TS_9d-10d-R | -1208.496488 |
| TS_9d-10d_TS | -1208.490411 |
| TS_9d-10d-F | -1208.49951 |
| TS10d-11d-R | -1208.509725 |
| TS10d-11d_TS | -1208.507262 |
| TS10d-11d-F | -1208.518239 |

**Table S4. Cartesian coordinates**

**IM1**

C -0.25100445 -1.61341834 1.48593932

C 1.22978193 -1.90064894 1.30080281

C 2.62719334 2.44738379 1.20594398

C 1.79285407 -2.23256290 0.13567164

C 3.96986539 1.92682307 0.61455671

C 3.92033787 0.47003975 0.23920104

H 2.39891513 1.87218028 2.11012422

H 4.74338314 2.08324421 1.37339839

H 2.74955417 3.49415975 1.50144754

H 4.24659797 2.55064974 -0.23929244

H 1.15061965 -2.34071018 -0.74025661

C 0.92273560 1.08269387 0.13269116

H -0.32383255 -0.70471376 2.09787164

C 1.51173199 2.28972901 0.20324825

H 1.25361881 0.34816752 0.85675771

C -1.29876897 -0.21923574 -0.57473580

C -1.16447876 -1.51550721 0.25487237

C 0.08246995 0.54641276 -0.96741052

C -2.06568039 0.89703701 -0.06273374

H -2.18629926 -1.76409301 0.56442538

H -0.88870992 -2.30256554 -0.45351694

H -1.71176801 -0.48707776 -1.55218828

H -0.13817478 1.29008859 -1.73635769

H 0.62902565 -0.27357503 -1.45026597

C -4.34942282 1.24906697 -0.81318544

C -4.54049987 -0.20500447 -1.13942501

C -2.85634557 1.71812829 -0.99350792

H -4.93067503 1.87446772 -1.49717401

H -4.67984599 1.49283122 0.19849283

H -4.45362376 -0.45718502 -2.19626084

H -2.79189374 2.78056716 -0.73956981

H -2.55741086 1.55337981 -2.03191992

C -4.80767614 -1.19586437 -0.27861570

H -0.65941443 -2.40700868 2.12538411

C -4.97717163 -1.02592151 1.20859860

H -4.31319750 -1.71196755 1.74781586

H -4.78685878 -0.01276950 1.56702607

H -5.99733830 -1.29597183 1.50095518

C -4.98600751 -2.60926202 -0.76469533

H -5.98311736 -2.97914437 -0.50411015

H -4.86228225 -2.68893761 -1.84606451

H -4.26668641 -3.27945462 -0.27995570

C -1.99778775 1.30178266 1.33753664

H -2.70007147 2.09453747 1.59522745

H -2.06147990 0.45792533 2.02853186

H -0.95798346 1.69581473 1.43212106

C 1.29098225 3.43505212 -0.74138068

H 2.21261065 3.64932172 -1.29482865

H 0.49735861 3.25845704 -1.47001861

H 1.04709393 4.34333405 -0.18093671

C 1.99131432 -1.73495312 2.59097007

H 1.49142201 -2.27764640 3.40047823

H 3.01753526 -2.09500512 2.53480337

H 2.01660485 -0.67814336 2.89136140

C 3.24976330 -2.45456047 -0.16620922

C 3.73574112 -1.52441590 -1.28723168

C 3.78501332 -0.03543916 -0.99276542

H 3.38963293 -3.48709293 -0.50728818

## H 3.87386549 -2.33963949 0.72221302

H 4.73345150 -1.84316921 -1.61635050

H 3.08716787 -1.67496424 -2.16270519

H 3.95892957 -0.22144782 1.08151497

C 3.68693801 0.80748000 -2.23877728

H 4.34605318 0.41456376 -3.02007784

H 2.66600979 0.76633174 -2.64299489

H 3.94203303 1.85556988 -2.08241311

**TS_1-2**

C -0.20514700 -1.00184200 1.16066000

C 1.11854500 -1.70223700 1.38048300

C 2.77460200 2.40443400 0.74291700

C 1.77247200 -2.35191400 0.41466100

C 3.70422000 1.63331200 -0.20550300

C 3.61776000 0.12908700 -0.10344300

H 2.80107700 1.94942700 1.74468600

H 4.72903600 1.94038900 0.03864400

H 3.17762600 3.41396100 0.88238600

H 3.52265100 1.97542600 -1.22959500

H 1.27879700 -2.51935600 -0.54259900

C 0.72067000 1.70024900 -0.49938500

H -0.02979600 0.02563300 1.48943000

C 1.32345200 2.56405200 0.33932900

H 1.30816200 0.86430700 -0.87342500

C -1.61622200 0.07131200 -0.76374500

C -0.69742000 -0.99349200 -0.28350900

C -0.64053700 1.87044200 -1.05582300

C -2.11513800 1.24226300 -0.18007300

H -1.20589100 -1.93380900 -0.54463700

H 0.16058600 -0.96342100 -0.97025200

H -1.96678300 -0.10200500 -1.78023900

H -1.03950300 2.87688100 -0.91595100

H -0.71075200 1.64909100 -2.12195500

C -4.58850300 1.03772500 -0.49323700

C -4.51691000 -0.37235600 -1.00412200

C -3.32281700 1.83781600 -0.88171000

H -5.44984900 1.54726400 -0.93630600

H -4.72807500 1.07513600 0.59075100

H -4.61634400 -0.47051500 -2.08649400

H -3.43393400 2.88958600 -0.60020600

H -3.19073200 1.79631900 -1.96963700

C -4.32680000 -1.49892300 -0.30016400

H -0.97743600 -1.40567700 1.82801500

C -4.19818000 -1.56908000 1.19790600

H -3.23207300 -2.00741400 1.47749300

H -4.30051100 -0.60335700 1.69445300

H -4.96461300 -2.23593600 1.60627600

C -4.26436800 -2.83606100 -0.98950200

H -5.08875900 -3.47513900 -0.65579000

H -4.32293000 -2.74239700 -2.07553400

H -3.34034500 -3.36774300 -0.73116800

C -2.01403800 1.56833500 1.29745800

H -2.59894100 2.46547500 1.51192700

H -2.41685300 0.74599200 1.89326500

H -0.98711600 1.74470900 1.61700300

C 0.67236300 3.78422100 0.93363200

H 1.15675900 4.68472400 0.54194300

H -0.39953000 3.86591900 0.74823700

H 0.82700900 3.79512500 2.01794800

C 1.67452400 -1.45467000 2.75832100

H 0.92451300 -1.69638600 3.51912100

H 2.56165500 -2.04944300 2.97191000

H 1.92834600 -0.39439500 2.89230300

C 3.21065500 -2.78492200 0.41582200

C 3.91384500 -2.25712300 -0.84363900

C 3.94242400 -0.75556000 -1.05462700

H 3.28681000 -3.87823000 0.41147400

H 3.73299600 -2.43514700 1.30996300

H 4.94880400 -2.62271100 -0.85688100

H 3.44044900 -2.71207200 -1.72589000

H 3.31105800 -0.24600000 0.87178100

C 4.42889700 -0.36423500 -2.42657900

H 5.41409900 -0.80514900 -2.61399000

H 3.76095000 -0.75731500 -3.20154800

H 4.51929900 0.71324500 -2.56368000

**IM2**

C -0.95846148 -1.72071151 0.72545681

C 0.38975888 -1.95773355 1.38519208

C 2.87853723 2.74764633 -0.49621996

C 1.50334583 -2.03175021 0.65301552

C 3.31728112 1.54965900 -1.33818476

C 3.23904461 0.24588410 -0.57667844

H 3.39938487 2.70518061 0.47010215

H 4.34956870 1.73032230 -1.65126490

H 3.23302463 3.67288115 -0.97062063

H 2.73237949 1.50902825 -2.26699127

H 1.41756954 -1.96199344 -0.43067511

C 0.43366176 2.30466600 -0.90013714

H -1.59837660 -1.20577460 1.45136404

C 1.40049504 2.94378459 -0.22671782

H 0.73152180 1.57392397 -1.65143503

C -1.96014586 0.15146391 -0.79596910

C -0.88345626 -0.90790608 -0.57856392

C -1.04939461 2.53482608 -0.76066802

C -1.83531482 1.46789775 0.04881570

H -0.84114317 -1.57907528 -1.44410855

H 0.06285057 -0.35679324 -0.59936129

H -1.91500891 0.44260789 -1.84943961

H -1.23863596 3.49513007 -0.27107335

H -1.50141908 2.61125110 -1.75863259

C -4.06671543 0.63843437 0.52492854

C -3.53324401 -0.29120312 -0.55897638

C -3.29087722 1.92681079 0.25257908

H -5.15194569 0.75780612 0.46565840

H -3.82498673 0.25992858 1.52352731

H -3.97039226 -0.02504052 -1.53499086

H -3.38044679 2.65656057 1.06094500

H -3.66902688 2.39790666 -0.66380993

C -3.70752589 -1.71823195 -0.46374435

H -1.44431739 -2.69384162 0.54576397

C -4.26407685 -2.36147974 0.73739408

H -4.15158924 -3.44531948 0.73921869

H -3.87474763 -1.91608247 1.65578577

H -5.34074395 -2.11846098 0.72689427

C -3.42164920 -2.56266568 -1.63791764

H -4.34693362 -3.09536325 -1.90233354

H -3.05605044 -2.00374744 -2.49829694

H -2.70897948 -3.34817416 -1.35681078

C -1.15859399 1.29735219 1.41695104

H -0.97915963 2.29207405 1.83602621

H -1.77735082 0.75371545 2.13650885

H -0.18784838 0.79843297 1.34199042

C 1.15477580 3.97885878 0.84168177

H 1.77531098 4.86217565 0.65919773

H 0.11805862 4.30732723 0.91713436

H 1.45559815 3.58219190 1.81836500

C 0.36250535 -2.07258140 2.88383308

H -0.32973755 -2.86156853 3.20066613

H 1.34498495 -2.30505790 3.29638425

H 0.01238108 -1.13823809 3.33994533

C 2.91880565 -2.14848007 1.15960027

C 3.92926486 -2.10688485 0.01484237

C 3.97210190 -0.84671743 -0.82490234

H 3.05327737 -3.08561003 1.71260694

H 3.12973649 -1.33934214 1.87020004

H 4.93728744 -2.28069023 0.41304278

H 3.73622663 -2.95700741 -0.65580337

H 2.54438141 0.23922144 0.26467411

C 4.94889802 -0.93993217 -1.96950349

H 5.96897584 -1.07968871 -1.59420162

H 4.72214405 -1.81357322 -2.59052196

H 4.93986834 -0.06193544 -2.61497750

**TS_2-3**

C -0.91174800 -1.40395900 0.52524500

C 0.23064200 -2.25428500 1.00081300

C 2.85266000 2.55894900 0.53762800

C 1.36438500 -2.28529700 0.28753300

C 3.46838100 1.57371200 -0.45832400

C 3.26151200 0.11309700 -0.10568800

H 3.08277700 2.22023300 1.55765300

H 4.54374200 1.77823700 -0.48665300

H 3.36882200 3.52360600 0.44176600

H 3.10816200 1.79099600 -1.47243700

H 1.36797600 -1.83249100 -0.69954300

C 0.57068600 2.49586800 -0.54105800

H -1.29539100 -0.83853600 1.37933100

C 1.36918300 2.86681200 0.46996300

H 1.01420400 1.91726900 -1.35052300

C -1.76765900 0.44003900 -1.00762200

C -0.63836500 -0.50920100 -0.66125800

C -0.89840900 2.82011400 -0.70346000

C -1.89147200 1.74897300 -0.17802100

H -0.38952000 -1.12789300 -1.53158800

H 0.25412000 0.08826500 -0.44933900

H -1.62309400 0.76296100 -2.04365900

H -1.14177600 3.75957200 -0.19572400

H -1.10918400 2.98583600 -1.76744800

C -4.13231500 0.83265300 -0.28885300

C -3.23823900 -0.22003600 -0.97652900

C -3.35236700 2.14281800 -0.45109400

H -5.12650100 0.86355300 -0.74168000

H -4.26879900 0.61375900 0.77275600

H -3.51089400 -0.28746700 -2.03909000

H -3.71001700 2.92850400 0.21945100

H -3.45042200 2.51234000 -1.47939500

C -3.32546800 -1.62820900 -0.51910300

H -1.81309000 -2.04463600 0.24554700

C -4.11097300 -1.99843200 0.68777400

H -3.99030100 -3.04768600 0.95734700

H -3.89295300 -1.35366000 1.54134500

H -5.16730600 -1.82552500 0.42783000

C -3.07376200 -2.68652800 -1.53824400

H -4.01437600 -2.78704500 -2.10199900

H -2.30169000 -2.39169100 -2.25283700

H -2.84730700 -3.65782000 -1.09741800

C -1.67803900 1.57739000 1.32981700

H -1.82727400 2.54258500 1.82282600

H -2.38275200 0.87488400 1.78560400

H -0.65982300 1.24861500 1.55733700

C 0.91079100 3.69689900 1.64271700

H 1.62215200 4.50807200 1.82788600

H -0.07600700 4.13964000 1.50577500

H 0.88469700 3.08905900 2.55484000

C 0.08369200 -2.85726600 2.37058100

H -0.82392000 -3.46837000 2.43353300

H 0.93064200 -3.49626400 2.62358500

H 0.00122400 -2.07915600 3.13827800

C 2.72199600 -2.73849600 0.74787900

C 3.79531400 -2.36577100 -0.27601200

C 3.93339600 -0.90471000 -0.66233000

H 2.74482900 -3.82335500 0.90807000

H 2.94762100 -2.28018500 1.71914200

H 4.77246900 -2.71491100 0.08085000

H 3.60791000 -2.93619200 -1.19734100

H 2.54501800 -0.08932500 0.69248900

C 4.94968400 -0.69568500 -1.75717300

H 4.70562100 -1.31360700 -2.62825400

H 5.01500600 0.34015000 -2.08899300

H 5.94436800 -1.00902100 -1.42040600

**IM3**

C -0.66551133 -1.42949745 0.37561909

C 0.52914982 -1.86646119 0.94220912

C 2.90492764 2.48583097 0.87497391

C 1.62369391 -2.01257111 0.10413360

C 3.71068155 1.67249327 -0.13434553

C 3.54169950 0.17951531 0.01329109

H 3.06301111 2.07752145 1.88418883

H 4.77046681 1.91558976 0.00568771

H 3.32610887 3.49806420 0.91543976

H 3.45967912 1.99660431 -1.15177676

H 1.51383134 -1.76199438 -0.94959405

C 0.75837179 2.06835253 -0.37928515

H -1.54928617 -1.50759740 1.00745207

C 1.41896773 2.64986994 0.63319105

H 1.32178855 1.40197401 -1.03411918

C -2.01901259 0.16128658 -1.08867785

C -0.89404292 -0.88521419 -0.97968887

C -0.67102183 2.36129900 -0.77932045

C -1.81379574 1.45409608 -0.25703656

H -1.16581767 -1.73708400 -1.62254473

H 0.02732187 -0.46271720 -1.38847109

H -1.94893067 0.49051740 -2.13294848

H -0.91550902 3.38248303 -0.46873647

H -0.72945008 2.37045679 -1.87553273

C -4.20057372 1.01580435 -0.37616209

C -3.52011289 -0.27492729 -0.91306702

C -3.16954586 2.13550645 -0.51623066

H -5.13443611 1.23680668 -0.89675981

H -4.45509892 0.88298120 0.68159738

H -3.89803995 -0.48013424 -1.92361762

H -3.34930007 2.97118524 0.16684466

H -3.17378998 2.53857959 -1.53664734

C -3.93408065 -1.51713253 -0.08585261

H -3.61083911 -1.38677113 0.95957655

C -5.46765176 -1.60878539 -0.07170852

H -5.79376140 -2.47072349 0.51537614

H -5.93817510 -0.72019163 0.35155110

H -5.84464292 -1.73753865 -1.09261146

C -3.40209070 -2.85294644 -0.62711542

H -3.53093452 -2.90303911 -1.71447636

H -2.34945259 -3.05180852 -0.40839451

H -3.96530251 -3.68427697 -0.19573073

C -1.64658477 1.18060751 1.24069786

H -1.69471459 2.11727080 1.80455811

H -2.43192536 0.52743243 1.63376723

H -0.66518219 0.74115403 1.46570268

C 0.78145405 3.58806920 1.62688254

H 1.36539300 4.51000846 1.71155542

H -0.24344688 3.85953805 1.37603281

H 0.77086663 3.13051228 2.62342571

C 0.60622272 -2.23940391 2.40126026

H -0.37526518 -2.17473519 2.87304046

H 0.98520043 -3.25541888 2.53823582

H 1.27691695 -1.55706180 2.93345708

C 2.92386785 -2.58631162 0.50523784

C 4.01993784 -2.23377407 -0.49380842

C 4.20272806 -0.74740162 -0.69652467

H 2.78883680 -3.68064545 0.55171784

H 3.17245499 -2.28021274 1.52838150

H 4.96892289 -2.68425754 -0.18213814

H 3.77876497 -2.69452925 -1.46194948

H 2.87325152 -0.12543828 0.82090967

C 5.20526156 -0.39870176 -1.76223661

H 4.91278443 -0.83362162 -2.72431826

H 5.32137293 0.67638876 -1.89520086

H 6.18545493 -0.81908272 -1.51176077

**TS_3-4**

C -0.83727015 -1.84282206 1.32576401

C 0.43640932 -2.10167376 1.84777012

C 2.79510151 2.80742969 -0.62209372

C 1.51868406 -1.92702957 1.00717704

C 3.17414852 1.52808408 -1.36795253

C 3.18018534 0.30886678 -0.47346881

H 3.43904879 2.90102741 0.26349840

H 4.17359612 1.67222686 -1.78843593

H 3.04670967 3.67283391 -1.24893349

H 2.50824366 1.38213839 -2.22815858

H 1.34652104 -1.70729700 -0.04442986

C 0.36015812 2.19402833 -0.61853555

H -1.66233536 -1.87540766 2.03997740

C 1.35540011 2.97510969 -0.17929661

H 0.61771954 1.37950364 -1.29748713

C -1.77970476 -0.12932256 -0.44933299

C -1.21827734 -1.56850027 -0.06022874

C -1.09936936 2.29663366 -0.26630259

C -1.63018057 1.08353932 0.54707802

H -2.03114123 -2.28193406 -0.25716502

H -0.41081651 -1.81565154 -0.74759526

H -1.21460560 0.09604856 -1.35750332

H -1.29866594 3.19917402 0.31695407

H -1.69805642 2.38688159 -1.18328467

C -3.92761427 0.17882474 0.57399819

C -3.29416574 -0.14291035 -0.77923418

C -3.08238235 1.36102381 1.04729822

H -4.99142427 0.41705915 0.51693871

H -3.82309865 -0.68566439 1.24831853

H -3.45134099 0.73918637 -1.42194037

H -3.11596123 1.51118578 2.12996132

H -3.46393538 2.27815709 0.58518561

C -3.86491925 -1.33705696 -1.55121357

H -3.83067152 -2.23176485 -0.90976707

C -5.33846366 -1.10021436 -1.89538940

H -5.73683332 -1.93988898 -2.47024508

H -5.95522829 -0.98727205 -1.00102338

H -5.44997489 -0.19608197 -2.50351272

C -3.06754413 -1.60798080 -2.82958718

H -3.07845501 -0.72305328 -3.47547382

H -2.02051361 -1.86079592 -2.63177505

H -3.50496987 -2.43552686 -3.39335458

C -0.70746193 0.91089699 1.75379501

H -0.65183842 1.86562422 2.28813258

H -1.09484348 0.18219610 2.47798045

H 0.31331078 0.64125161 1.47296617

C 1.18119072 4.13544176 0.76680593

H 1.63963758 5.03633805 0.34548988

H 0.14231307 4.36722821 0.99785723

H 1.70262026 3.93261575 1.70937039

C 0.62207155 -2.38058523 3.31682035

H -0.33827242 -2.48253728 3.82428962

H 1.18572202 -3.30362199 3.47644408

H 1.17357882 -1.56561496 3.79659137

C 2.93207308 -1.94918323 1.43177406

C 3.90755220 -1.99368497 0.25917986

C 3.86009569 -0.82347499 -0.70228787

H 3.09504631 -2.78354224 2.12693369

H 3.09440315 -1.04597989 2.04705852

H 4.92659321 -2.08280530 0.65385929

H 3.73620762 -2.91971107 -0.30605588

H 2.60607883 0.41968368 0.44884196

C 4.68249598 -1.05768452 -1.94218832

H 4.31875142 -1.94072668 -2.47917497

H 4.66027859 -0.21515595 -2.63186230

H 5.72671976 -1.25816612 -1.67842196

**IM4**

C -0.76451546 -1.42726034 2.23453770

C 0.60779455 -1.66928069 2.15023620

C 2.70273893 2.60840249 -0.18431067

C 1.12782126 -2.03208875 0.91844302

C 2.96110136 1.52345127 -1.22610295

C 2.76204072 0.12067323 -0.69515813

H 3.30590236 2.39663033 0.71063968

H 3.99926991 1.61911557 -1.56562254

H 3.08365374 3.56587375 -0.56214507

H 2.34183379 1.70263196 -2.11267250

H 0.44661426 -2.10660522 0.07226507

C 0.23757210 2.22695870 -0.36876570

H -1.14441480 -1.27807808 3.24489270

C 1.26747338 2.82332353 0.24500152

H 0.46187347 1.54714959 -1.18992907

C -1.52327974 -0.12296894 0.16889459

C -1.74734319 -1.34221803 1.13594046

C -1.22536395 2.38420997 -0.05850300

C -1.81569705 1.24775772 0.82853661

H -2.75816250 -1.30573319 1.55100076

H -1.67473678 -2.24876274 0.52297101

H -0.48385542 -0.17659496 -0.17533510

H -1.42445176 3.32873987 0.45637843

H -1.78654843 2.43669152 -0.99794916

C -3.80788217 0.48215200 -0.42096318

C -2.52977171 -0.16482812 -1.00387186

C -3.35831660 1.32492254 0.79546442

H -4.32349822 1.08222518 -1.17373662

H -4.51354191 -0.29773718 -0.11152878

H -2.11308478 0.50859888 -1.76657817

H -3.77750350 0.91815851 1.72264636

H -3.69369093 2.36434169 0.73663135

C -2.75895015 -1.50290468 -1.71485998

H -3.16462906 -2.22575200 -0.99041712

C -3.79662486 -1.34264444 -2.82969003

H -3.94807882 -2.28844905 -3.35593178

H -4.76433678 -1.01771160 -2.44081291

H -3.45912881 -0.60126763 -3.56247771

C -1.45864900 -2.05589277 -2.30232024

H -1.03007253 -1.34272568 -3.01522688

H -0.69577623 -2.24757396 -1.53863803

H -1.63380899 -2.99618113 -2.83102352

C -1.25922677 1.41425840 2.23980295

H -1.47061260 2.42037086 2.61566597

H -1.73572090 0.72520093 2.94642015

H -0.17109726 1.27817757 2.26392281

C 1.13267499 3.79690767 1.38679053

H 1.68672559 4.71561723 1.16757449

H 0.10044466 4.06984741 1.60540937

H 1.57147315 3.37794213 2.30018652

C 1.48676997 -1.58689981 3.37303795

H 0.89715336 -1.38417746 4.26806242

H 2.03730237 -2.51783221 3.53211508

H 2.21842984 -0.77993337 3.26493105

C 2.51872847 -2.44521094 0.66506783

C 2.89005443 -2.39071238 -0.81277184

C 2.96007681 -1.00110500 -1.40171731

H 2.57833322 -3.49097800 1.01946152

H 3.21773949 -1.88581346 1.29739198

H 3.85916158 -2.88201048 -0.95818380

H 2.16940323 -2.99389819 -1.38344906

H 2.51570508 0.05955924 0.36741121

C 3.34422021 -0.99103494 -2.85712998

H 2.65822103 -1.61486880 -3.44071016

H 3.34135736 0.01090901 -3.28509231

H 4.34646370 -1.41352434 -2.99029071

**TS_4-5a**

C -0.00735100 0.24326100 2.11642900

C 1.26505100 -0.17286500 1.87952800

C 3.00682800 1.87327500 -0.61518400

C 1.47283200 -1.24909700 0.93591800

C 3.26941300 0.50367900 -1.24839400

C 3.02310700 -0.72513000 -0.40237400

H 3.30653700 1.86988700 0.44221200

H 4.33912800 0.44995800 -1.49392100

H 3.68159400 2.59450000 -1.09188600

H 2.73871200 0.43892800 -2.20119300

H 0.62495500 -1.43927700 0.27728200

C 0.59267500 1.69718400 -1.22365200

H -0.11373700 0.94652700 2.94165000

C 1.60150700 2.42292100 -0.73193600

H 0.81673000 0.67308600 -1.52191900

C -1.61848100 0.14897200 0.05976000

C -1.30093200 -0.24985000 1.52103200

C -0.84952900 2.09221500 -1.39731000

C -1.74846800 1.69714700 -0.19946100

H -2.11488200 0.08344800 2.17116700

H -1.30233500 -1.34323100 1.58421900

H -0.81596200 -0.25477600 -0.56609900

H -0.95217000 3.16834900 -1.57489700

H -1.23696600 1.59416900 -2.29504400

C -3.97348800 0.65606300 -0.06189000

C -2.97276800 -0.44497100 -0.41896400

C -3.25137000 1.91431900 -0.54325200

H -4.94694400 0.52440000 -0.53908500

H -4.14081200 0.68382100 1.02384200

H -2.91939300 -0.47199600 -1.52038800

H -3.64164500 2.83272100 -0.09704100

H -3.37803600 2.00469300 -1.62840800

C -3.31698600 -1.86824600 0.03393000

H -3.33328500 -1.89893900 1.13241500

C -4.70773400 -2.27784100 -0.45734300

H -4.91793500 -3.31956600 -0.20063800

H -5.49176600 -1.66112000 -0.01264700

H -4.77586800 -2.18051600 -1.54677700

C -2.27775300 -2.87194000 -0.47621500

H -2.26342800 -2.87385600 -1.57205000

H -1.26174500 -2.63781100 -0.13312400

H -2.51266600 -3.88642800 -0.14355000

C -1.38853400 2.57703900 1.00218300

H -1.58969500 3.62467400 0.75751400

H -1.99393800 2.33300500 1.88178300

H -0.32949500 2.50234600 1.26551100

C 1.48329900 3.85265600 -0.27594200

H 2.19127200 4.48628700 -0.82073400

H 0.48250600 4.25973300 -0.41678600

H 1.73473700 3.94016700 0.78796800

C 2.39955400 0.27834500 2.76604000

H 2.10982500 1.16191500 3.33692200

H 2.68072000 -0.50058700 3.48213700

H 3.29736900 0.53731700 2.19613200

C 2.22733000 -2.50902800 1.30342200

C 2.54944900 -3.12737100 -0.05134500

C 2.78103900 -1.96376200 -0.96923100

H 1.58589900 -3.14430500 1.92314000

H 3.12322500 -2.28103000 1.88636800

H 3.39594700 -3.82305200 -0.06227300

H 1.68348400 -3.68589800 -0.43008800

H 3.54928200 -0.71358700 0.55165200

C 2.59431500 -2.16147500 -2.43424100

H 1.62585000 -1.73753300 -2.73611100

H 3.36306800 -1.64751200 -3.01620200

H 2.58383800 -3.22192300 -2.69009600

**IM5a**

C 0.03449573 0.18275287 2.05808814

C 1.27985679 -0.08141074 1.64677853

C 2.88521900 1.75433537 -0.92225274

C 1.54452967 -1.03333851 0.50013471

C 3.02911273 0.30267560 -1.38729232

C 2.84971995 -0.80820346 -0.33074476

H 3.42785694 1.88999514 0.02142288

H 4.04024850 0.19701278 -1.79380024

H 3.43480726 2.37545874 -1.64222416

H 2.34615562 0.10156797 -2.22007680

H 0.70584762 -1.01795963 -0.20415667

C 0.40729701 1.76058003 -1.29250096

H -0.05116944 0.79979170 2.95022751

C 1.49679472 2.35031823 -0.79263094

H 0.53981297 0.79084115 -1.76935720

C -1.70618869 0.19845417 0.09183949

C -1.27411824 -0.31559105 1.48459384

C -1.02144153 2.24221575 -1.28011035

C -1.84619091 1.76097478 -0.05632072

H -2.06440319 -0.09439962 2.20957839

H -1.22749442 -1.41062039 1.43636747

H -0.96618687 -0.15700515 -0.62959048

H -1.07558225 3.33554824 -1.33328036

H -1.50618128 1.86601345 -2.18935836

C -4.06053923 0.71490000 0.16741980

C -3.09394438 -0.36022715 -0.33226003

C -3.36791373 1.99885114 -0.29021978

H -5.06909981 0.61467446 -0.24022156

H -4.14135761 0.67724443 1.26263003

H -3.12310323 -0.31362789 -1.43435714

H -3.72487644 2.89089899 0.23132919

H -3.56793935 2.14840595 -1.35781981

C -3.40545791 -1.81245808 0.04580535

H -3.32679399 -1.92250643 1.13578497

C -4.83287763 -2.19238493 -0.35475818

H -5.02290899 -3.25139077 -0.15897102

H -5.57527609 -1.61325488 0.19893524

H -4.99340335 -2.01492751 -1.42436562

C -2.41327954 -2.77083860 -0.62226811

H -2.51358072 -2.71365476 -1.71223493

H -1.37088796 -2.53180389 -0.37434456

H -2.60165266 -3.80588113 -0.32372041

C -1.41686037 2.56435078 1.17589106

H -1.64337187 3.62294501 1.01168464

H -1.96561893 2.25129000 2.07094135

H -0.34788417 2.47671980 1.37555731

C 1.50947926 3.70096767 -0.12914156

H 2.25970333 4.34948121 -0.59532490

H 0.54333417 4.20235439 -0.18060787

H 1.78351887 3.60636382 0.92888444

C 2.47254257 0.40652749 2.42890376

H 2.15128335 0.94596755 3.32110740

H 3.11074214 -0.42164771 2.76563406

H 3.10774809 1.08552430 1.84971927

C 1.72627489 -2.47608315 1.02094828

C 2.45444563 -3.18420673 -0.12191418

C 3.23581546 -2.14385483 -0.78894090

H 0.77530950 -2.94535730 1.27444284

H 2.35409495 -2.48020804 1.91802702

H 3.00591483 -4.10702607 0.07913758

H 1.72646369 -3.44041272 -0.92496428

H 3.66285546 -0.68705408 0.43551626

C 4.22992207 -2.42033038 -1.82907689

H 3.79606984 -2.07699378 -2.78244415

H 5.12411642 -1.80284235 -1.68930091

H 4.48839816 -3.47521315 -1.90632601

**TS_5a-6a**

C -0.05649900 0.22317600 1.99739600

C 1.22615900 0.02429100 1.67234300

C 2.65690200 2.11824500 -0.53001000

C 1.60999400 -0.90617000 0.54571600

C 3.06672400 0.75998500 -1.11013500

C 2.82346300 -0.52144200 -0.32516900

H 2.94479600 2.18907700 0.52474800

H 4.13592500 0.80495400 -1.33561000

H 3.27988300 2.86689600 -1.03609100

H 2.57417500 0.61561700 -2.08021100

H 0.77765800 -0.99606700 -0.16322700

C 0.28297000 1.80635000 -1.26121400

H -0.23540900 0.84706600 2.87155200

C 1.21574700 2.55735000 -0.67065700

H 0.59255200 0.83752700 -1.65112800

C -1.73223700 0.00314300 -0.03759000

C -1.29023300 -0.40989500 1.38549700

C -1.18881100 2.08685400 -1.41520500

C -2.04464300 1.53437400 -0.24535400

H -2.12353500 -0.24478700 2.07626300

H -1.13312400 -1.49529300 1.38165000

H -0.93573200 -0.28901000 -0.72808300

H -1.38504600 3.15937800 -1.52350900

H -1.53133100 1.61425600 -2.34390900

C -4.13368300 0.26078300 -0.08915800

C -3.03413700 -0.72852500 -0.47981200

C -3.55898000 1.58229300 -0.59755300

H -5.10389800 0.02633400 -0.53297900

H -4.26434500 0.27871900 1.00191400

H -3.01700700 -0.74765900 -1.58268400

H -4.04805900 2.46221800 -0.17111500

H -3.69426500 1.63176300 -1.68461900

C -3.20814000 -2.18278400 -0.02797700

H -3.15296800 -2.22591600 1.06809900

C -4.57676700 -2.72879200 -0.44210200

H -4.66169700 -3.79063700 -0.19490300

H -5.39100400 -2.20350700 0.06210400

H -4.72315100 -2.62356000 -1.52322400

C -2.10474100 -3.06871700 -0.61674100

H -2.18305400 -3.08727700 -1.70973500

H -1.09891000 -2.70831000 -0.36577600

H -2.19146700 -4.09835200 -0.25880400

C -1.81560200 2.39938800 0.99887300

H -2.13903900 3.42462000 0.79181100

H -2.40411300 2.04083500 1.85025700

H -0.76529400 2.43265000 1.29282500

C 0.97452700 3.92737900 -0.09618500

H 1.67785000 4.65051500 -0.52362200

H -0.03781700 4.28569700 -0.27941000

H 1.13905200 3.92533300 0.98818900

C 2.34205500 0.51980200 2.56288100

H 2.01087100 1.37193300 3.15977600

H 2.66847500 -0.25741100 3.26433100

H 3.23275700 0.83711700 2.00889500

C 1.98873800 -2.31278700 1.06457700

C 2.89916700 -2.90212900 -0.01478100

C 3.51837400 -1.71011200 -0.66896600

H 1.10634800 -2.92165600 1.26241100

H 2.54691200 -2.21847400 2.00246300

H 3.62851900 -3.64242300 0.32010900

H 2.30740700 -3.36840000 -0.81958700

H 3.78731400 -0.75466000 0.40911900

C 4.65128000 -1.83130200 -1.62175000

H 4.20565300 -2.06546200 -2.59850200

H 5.22361100 -0.91171000 -1.74023700

H 5.31074800 -2.65648400 -1.35108800

**IM6a**

C -0.05271192 0.32552497 2.08334183

C 1.24252364 0.12184300 1.81501795

C 2.66900330 2.15198493 -0.33852523

C 1.66020105 -0.82097660 0.71913893

C 3.12579455 0.78433682 -0.84122435

C 2.71326218 -0.49960606 -0.24616023

H 2.84023277 2.23545052 0.73963642

H 4.22685623 0.73986972 -0.85748906

H 3.35176727 2.88289419 -0.78981118

H 2.88275610 0.66822555 -1.91464279

H 0.85508138 -0.84219204 -0.06682331

C 0.37383192 1.79881280 -1.26384502

H -0.26785479 0.96192775 2.94036440

C 1.24966821 2.58149090 -0.62826725

H 0.72983298 0.83286324 -1.62374263

C -1.63058908 0.00293717 -0.02763959

C -1.25346538 -0.33534559 1.43412304

C -1.09673900 2.03386768 -1.48816667

C -1.97752129 1.51221849 -0.32055343

H -2.12136951 -0.14832170 2.07396901

H -1.08635047 -1.41832442 1.48890123

H -0.79404033 -0.29623911 -0.66871341

H -1.31562298 3.09475139 -1.65181653

H -1.39461627 1.51103722 -2.40481335

C -4.03185320 0.17554486 -0.19864975

C -2.88374428 -0.78796271 -0.50516347

C -3.47948214 1.49911519 -0.72770525

H -4.97175513 -0.10750313 -0.67764351

H -4.21419412 0.22502682 0.88380748

H -2.81225637 -0.83904950 -1.60480736

H -4.01060468 2.37500084 -0.34583638

H -3.57702970 1.51260844 -1.81962649

C -3.02721827 -2.23338839 -0.01679216

H -3.03327445 -2.24174602 1.08149571

C -4.34800284 -2.84553964 -0.48929616

H -4.40676554 -3.90166447 -0.21240394

H -5.20888982 -2.33770069 -0.04876166

H -4.43598007 -2.77936679 -1.57971102

C -1.85759597 -3.08961504 -0.51514589

H -1.87317658 -3.14568791 -1.60957880

H -0.88289023 -2.67812809 -0.22104100

H -1.91932382 -4.11011942 -0.12771897

C -1.81944524 2.43242671 0.89439222

H -2.13756958 3.44551064 0.62851335

H -2.45237270 2.10403943 1.72572345

H -0.78786746 2.48530575 1.24661401

C 0.95305329 3.96660418 -0.12034758

H 1.67485409 4.68157573 -0.53003342

H -0.04823455 4.30333183 -0.38550921

H 1.04611216 4.00911720 0.97131836

C 2.34652655 0.60300509 2.72922824

H 2.03547185 1.48896615 3.28676837

H 2.60354801 -0.17181731 3.46049323

H 3.26848271 0.84735519 2.19151655

C 1.88163300 -2.30489175 1.09904039

C 2.48567670 -2.89174601 -0.17239321

C 3.38687434 -1.74996553 -0.67442668

H 0.94863751 -2.77473106 1.41268428

H 2.58704229 -2.35638928 1.93610413

H 3.04858176 -3.81235046 -0.01605490

H 1.70543214 -3.09077917 -0.91751765

H 4.24499196 -1.70836834 0.03858161

C 3.92155299 -1.84539549 -2.09838782

H 3.09902274 -1.78065389 -2.81662754

H 4.65058382 -1.06619371 -2.32865721

H 4.41118456 -2.81076869 -2.23509018

**TS_6a-7a**

C 0.21727357 0.42773784 2.10478920

C 1.41496793 -0.11710179 1.83585118

C 3.25708661 1.22985274 -0.56531217

C 1.57479111 -1.22493487 0.87029167

C 3.26390101 -0.24667683 -0.95531875

C 2.35323854 -1.26287808 -0.31114907

H 3.52782193 1.35483589 0.48775050

H 4.26951139 -0.65707432 -0.78245302

H 4.08075770 1.69607570 -1.12113730

H 3.10716371 -0.33234435 -2.03600931

H 1.12412082 -0.87642937 -0.35630049

C 0.91489194 1.49314289 -1.40549301

H 0.23494655 1.21390717 2.85831221

C 1.99575339 2.02195822 -0.82123613

H 0.99607485 0.47431533 -1.78971957

C -1.46874297 0.41319034 0.06828924

C -1.14995804 0.09062700 1.55042378

C -0.44839614 2.10794550 -1.58081214

C -1.37423958 1.93508662 -0.34558022

H -1.89185385 0.58039104 2.18816433

H -1.31259226 -0.98295521 1.69277739

H -0.77632621 -0.17607279 -0.54905650

H -0.38078836 3.17628480 -1.81624936

H -0.93264400 1.63366363 -2.44211239

C -3.72069846 1.28228928 -0.05950969

C -2.92550775 -0.00092546 -0.30767761

C -2.83178079 2.33772161 -0.71114146

H -4.72348141 1.25527242 -0.49096218

H -3.83075665 1.46326426 1.01885294

H -2.92508028 -0.15841574 -1.39925075

H -3.06200841 3.35755868 -0.39199563

H -2.97414585 2.29948298 -1.79738880

C -3.48732899 -1.28406281 0.31512057

H -3.43890296 -1.20157032 1.40960817

C -4.95678479 -1.48293861 -0.06509480

H -5.32286175 -2.44573393 0.30148305

H -5.59438576 -0.70280658 0.35623678

H -5.07865604 -1.47086417 -1.15417682

C -2.67409040 -2.50257672 -0.12886276

H -2.76409279 -2.64121606 -1.21247329

H -1.60849264 -2.38970945 0.10022257

H -3.02681783 -3.41704735 0.35548637

C -0.89972190 2.85394007 0.78489939

H -0.93858167 3.89483538 0.44923803

H -1.55005576 2.77548127 1.66270361

H 0.12598187 2.64229200 1.09035894

C 2.11054626 3.45347080 -0.36900389

H 2.98964376 3.91606450 -0.83074141

H 1.23650368 4.04863559 -0.63009123

H 2.25329594 3.51633620 0.71620090

C 2.66377026 0.24644526 2.61989045

H 2.68281172 -0.29715500 3.56927199

H 3.58026185 -0.01021078 2.08370747

H 2.68461657 1.31528516 2.84521512

C 1.02472122 -2.60827391 1.14638263

C 1.15525731 -3.34892056 -0.18392914

C 2.36302169 -2.68900683 -0.86349191

H 0.02705644 -2.59609045 1.58515249

H 1.70579904 -3.02267364 1.90423957

H 1.27548413 -4.42602746 -0.06683284

H 0.26084921 -3.17475013 -0.79595529

H 3.28852982 -3.12533496 -0.45605058

C 2.36948340 -2.81040246 -2.38290713

H 1.52541801 -2.26617591 -2.82046250

H 3.29434935 -2.43710399 -2.82676684

H 2.27019471 -3.86084931 -2.66478058

**IM7a**

C 0.12412438 0.34195039 2.17954038

C 1.34041034 -0.10664012 1.77573089

C 3.27919126 1.21456467 -0.77053976

C 1.51316803 -1.19902662 0.85967412

C 3.27360739 -0.30155587 -1.01418049

C 2.22866856 -1.25029570 -0.41629965

H 3.71825390 1.43271568 0.20706916

H 4.25722411 -0.69227811 -0.72457518

H 4.00278377 1.63132212 -1.48405887

H 3.20303162 -0.47316553 -2.09337168

H 1.23602571 -1.02157583 -0.91140443

C 0.86378781 1.51453320 -1.39705436

H 0.16987531 1.15633947 2.90124831

C 1.99630723 2.01095568 -0.88433030

H 0.90793556 0.51571420 -1.82855146

C -1.48730555 0.41675017 0.16724056

C -1.24498011 0.02061440 1.65295689

C -0.50241979 2.14811655 -1.46007110

C -1.36202221 1.95159607 -0.17684055

H -1.98419888 0.50406625 2.29767119

H -1.42716250 -1.05496350 1.73589312

H -0.75099615 -0.15074633 -0.41660961

H -0.44147178 3.22339644 -1.66392396

H -1.04479349 1.70834326 -2.30497556

C -3.72982638 1.29002922 0.04762487

C -2.92094643 0.03852499 -0.29850526

C -2.82575805 2.41630614 -0.45122806

H -4.71481127 1.30721299 -0.42329677

H -3.88558462 1.35355290 1.13336771

H -2.87687816 -0.01575085 -1.39945516

H -3.04036524 3.37778924 0.02228213

H -2.97956109 2.54931675 -1.52797324

C -3.49551697 -1.29754600 0.18440016

H -3.51317512 -1.29906084 1.28364622

C -4.93679481 -1.48312243 -0.29654411

H -5.31011518 -2.47343726 -0.02263456

H -5.60938128 -0.74205902 0.14062148

H -4.99373547 -1.39337579 -1.38717955

C -2.63553762 -2.46707907 -0.30090311

H -2.64702777 -2.51496645 -1.39588805

H -1.58978095 -2.36612819 0.01115457

H -3.00875867 -3.42167545 0.07932473

C -0.78445909 2.81335406 0.94977891

H -0.81965764 3.86703294 0.65640198

H -1.37293828 2.71852028 1.86915726

H 0.25849208 2.57000959 1.16748655

C 2.15835795 3.43636058 -0.42063299

H 3.00205551 3.89689701 -0.94635038

H 1.27234559 4.04275048 -0.60597947

H 2.38940672 3.49396836 0.64970857

C 2.60245334 0.54705779 2.32367759

H 2.60703284 0.43316631 3.41080578

H 3.51114275 0.07778690 1.94212814

H 2.63349582 1.61351551 2.08868639

C 0.96473488 -2.55319723 1.14262809

C 1.17887018 -3.38656033 -0.11906269

C 2.42900033 -2.75427084 -0.73972605

H -0.01920896 -2.56114907 1.61433822

H 1.66828894 -2.89612480 1.92699941

H 1.29144500 -4.45010550 0.09386599

H 0.32831573 -3.26532550 -0.80192454

H 3.31812317 -3.08883731 -0.18605417

C 2.60675703 -3.06290378 -2.21943817

H 1.78442713 -2.63953471 -2.80708625

H 3.54824586 -2.67174476 -2.61032046

H 2.60926962 -4.14392443 -2.37741189

**TS_7a-8a**

C -0.34268000 -0.58045400 1.93405000

C -1.62545800 -0.22369400 1.77710300

C -2.80307900 -1.74855700 -0.84238400

C -2.02317700 0.88049900 0.91424200

C -2.94720200 -0.28969100 -1.26950400

C -2.17839400 0.83682300 -0.53258200

H -3.16384500 -1.87348200 0.18604700

H -4.00479400 -0.01364300 -1.19244100

H -3.51249000 -2.32362700 -1.45303100

H -2.68478000 -0.18415000 -2.32660100

H -1.07685900 0.67689900 -0.71409900

C -0.40580200 -1.80678300 -1.52749500

H -0.17991200 -1.38433600 2.64840500

C -1.44261500 -2.40178500 -0.93095800

H -0.60014500 -0.85585100 -2.02377200

C 1.48337200 -0.21898200 0.01121200

C 0.89747000 0.11686200 1.40935100

C 1.04315500 -2.22091200 -1.54063900

C 1.81664500 -1.73361800 -0.28104800

H 1.67888200 -0.03966700 2.16134200

H 0.69877500 1.19658500 1.42308900

H 0.76031900 0.13512000 -0.72890200

H 1.15796700 -3.30719800 -1.62737600

H 1.51578600 -1.78781500 -2.42918500

C 3.88011100 -0.49253200 0.16390600

C 2.82339200 0.53545400 -0.24258900

C 3.35387200 -1.75639200 -0.51489200

H 4.88752700 -0.22683800 -0.16378300

H 3.90776900 -0.60930100 1.25657700

H 2.90633100 0.63783200 -1.33751200

H 3.80868700 -2.67596800 -0.13758100

H 3.57718300 -1.70046400 -1.58652700

C 2.95434600 1.95204600 0.32734700

H 2.77526900 1.92497200 1.41079000

C 4.36285800 2.50875400 0.10830300

H 4.42071600 3.55282500 0.42785400

H 5.11143700 1.94609100 0.67052700

H 4.63274800 2.46744900 -0.95298100

C 1.92624500 2.88228000 -0.32549600

H 2.15355500 3.00567500 -1.39046800

H 0.90688700 2.48099900 -0.25698100

H 1.93230800 3.87447200 0.13470300

C 1.50944000 -2.66985800 0.89214300

H 1.86099300 -3.67849300 0.65413700

H 2.02557500 -2.35371700 1.80584500

H 0.44126300 -2.73639200 1.09768200

C -1.40896800 -3.76969100 -0.30393400

H -1.59903400 -3.71327300 0.77542700

H -2.20257200 -4.39289100 -0.73066000

H -0.45774200 -4.27968100 -0.45563900

C -2.77215500 -0.77012300 2.61632400

H -2.47050500 -1.73083600 3.03796900

H -3.00327500 -0.08774600 3.44051700

H -3.68791300 -0.92201600 2.03864900

C -2.25718800 2.24274900 1.42202600

C -2.11452500 3.15567700 0.19316600

C -2.55944000 2.26677200 -0.97898900

H -1.68721600 2.48583800 2.32150600

H -3.32569900 2.19080100 1.72350800

H -2.70387300 4.06859900 0.28366800

H -1.06410200 3.44364000 0.07755300

H -3.65525800 2.29791800 -1.05106700

C -1.95261800 2.66637500 -2.31852000

H -0.85922100 2.60424300 -2.27750200

H -2.30520300 2.02530200 -3.13017300

H -2.22117200 3.69482300 -2.57038000

**IM8a**

C -0.12252665 -0.22216948 1.77396175

C -1.50675853 -0.19681848 1.76872283

C -2.60188530 -1.80172713 -0.91172536

C -2.27698732 0.49920942 0.82137010

C -2.68391412 -0.36298245 -1.42937411

C -1.97523096 0.78130116 -0.59559990

H -3.08549102 -1.86038719 0.07622051

H -3.74180829 -0.09826344 -1.50562363

H -3.23145636 -2.42502599 -1.56026325

H -2.27545341 -0.28546718 -2.44120086

H -0.90765382 0.78498266 -0.82274586

C -0.17570512 -1.92753677 -1.43524613

H 0.26887568 -0.76447141 2.63255780

C -1.23311934 -2.43807429 -0.79726971

H -0.35858795 -1.05937069 -2.06468658

C 1.64947544 -0.06076680 -0.22950410

C 0.93725925 0.53294728 1.03861916

C 1.27416828 -2.33049130 -1.35661764

C 2.02095993 -1.59354066 -0.21079542

H 1.71414634 0.71079943 1.79113195

H 0.54262747 1.51241868 0.74702923

H 0.97248431 0.13501773 -1.06171196

H 1.39658015 -3.41124059 -1.22645429

H 1.75347993 -2.07503291 -2.30824378

C 4.03772392 -0.25366318 0.10444525

C 2.99269715 0.67812351 -0.51071751

C 3.56277731 -1.61562032 -0.39962626

H 5.05732198 -0.01920309 -0.20824810

H 4.00934783 -0.20257649 1.20256594

H 3.13489936 0.60882741 -1.60183852

H 4.02466973 -2.46132687 0.11619938

H 3.81338518 -1.70652021 -1.46270193

C 3.07047846 2.17077374 -0.17083817

H 2.78441836 2.32265249 0.87914952

C 4.49635225 2.70177914 -0.33524704

H 4.52183186 3.78487314 -0.18908700

H 5.18373961 2.25108873 0.38436274

H 4.87168744 2.49160875 -1.34305937

C 2.11562450 2.96641834 -1.06589824

H 2.46885253 2.94606104 -2.10251110

H 1.10174444 2.54988415 -1.06250421

H 2.05299310 4.01248699 -0.75429375

C 1.71055553 -2.30086907 1.11109520

H 2.09378384 -3.32517468 1.07635834

H 2.18982146 -1.80397162 1.96269551

H 0.63389469 -2.37463695 1.29230782

C -1.21689720 -3.68659867 0.04253183

H -1.45730078 -3.45852382 1.08955179

H -1.98524124 -4.38392081 -0.30899730

H -0.25561995 -4.20071104 0.01796209

C -2.24033983 -0.81442104 2.94645249

H -1.57250411 -1.45828840 3.52070509

H -2.62608744 -0.04614070 3.62411472

H -3.08749986 -1.42106296 2.61482421

C -3.63838459 1.02826611 1.13384514

C -4.00158804 1.98030663 -0.02623776

C -2.69583519 2.12580083 -0.84740842

H -3.61742786 1.51503831 2.11692660

H -4.33881552 0.18725272 1.25143629

H -4.81289286 1.56615502 -0.62711455

H -4.34389157 2.94771761 0.34593360

H -2.90886731 2.24294779 -1.91432851

C -1.85772455 3.30406633 -0.35587297

H -1.62807542 3.20725992 0.71391991

H -0.91126765 3.37786326 -0.89737695

H -2.39529071 4.24519157 -0.49254792

**TS_8a-9a**

C -0.25759411 -0.48795298 1.41820353

C -1.57998840 -0.12941617 1.68307248

C -2.65071392 -1.84633738 -0.65700174

C -2.32833959 0.63083178 0.79330714

C -2.78147767 -0.47895345 -1.33990478

C -2.08016778 0.76055972 -0.66786950

H -3.10706908 -1.80063159 0.34307962

H -3.84956431 -0.25270227 -1.40900701

H -3.27771683 -2.55854540 -1.20913498

H -2.41313435 -0.52248487 -2.36975828

H -1.01915367 0.75756811 -0.93014549

C -0.23403496 -1.99324222 -1.23236925

H 0.10157967 -1.34142891 1.98778672

C -1.27256447 -2.45569148 -0.51738782

H -0.44372369 -1.15927048 -1.89814336

C 1.69153950 -0.09401551 -0.36265454

C 0.81185973 0.36525616 0.83113155

C 1.19720713 -2.45776257 -1.27761580

C 2.13706326 -1.60335484 -0.38316737

H 1.47356587 0.48458818 1.70679184

H 0.40937740 1.35896074 0.62444924

H 1.07524966 0.08707396 -1.24960735

H 1.29286026 -3.51283349 -1.00203462

H 1.53263474 -2.37564362 -2.31764805

C 4.13901373 -0.25909073 -0.30368753

C 3.00883459 0.75177903 -0.53883152

C 3.56993393 -1.51805041 -0.95010424

H 5.08159849 0.06226869 -0.75162070

H 4.31980831 -0.41278085 0.76834069

H 3.05471651 0.99834863 -1.61020376

H 4.14614620 -2.42642118 -0.75630392

H 3.53515966 -1.37361078 -2.03719402

C 3.11886152 2.09472827 0.19897290

H 2.83488090 1.95933805 1.25235113

C 4.55829340 2.61698488 0.18374017

H 4.60799690 3.61144008 0.63499326

H 5.23740888 1.96356528 0.73605389

H 4.92608991 2.69915423 -0.84517903

C 2.20046454 3.14402735 -0.43809610

H 2.57860862 3.41047435 -1.43100590

H 1.17317317 2.78917139 -0.57093798

H 2.16590564 4.05801107 0.16053997

C 2.22264519 -2.25525187 1.01130335

H 2.92220477 -3.09479831 0.97471202

H 2.57904518 -1.57354746 1.78995055

H 1.26464006 -2.67595832 1.32580644

C -1.22118153 -3.64758155 0.39970396

H -1.44729207 -3.35476538 1.43348995

H -1.98699837 -4.37583336 0.11203243

H -0.25422801 -4.15142083 0.38792878

C -2.24493113 -0.64715969 2.94591913

H -1.59991814 -1.35479154 3.46905651

H -2.44717065 0.18058729 3.63314117

H -3.19682156 -1.14179665 2.73172390

C -3.64246440 1.26108037 1.12229196

C -4.06442025 2.04299261 -0.14097626

C -2.80586882 2.06815769 -1.04266573

H -3.50898874 1.89866338 2.00656623

H -4.37116851 0.49772277 1.43036656

H -4.89839713 1.54283966 -0.63748365

H -4.40000599 3.05272610 0.10313440

H -3.08148458 2.06050578 -2.10190832

C -1.92392145 3.28106246 -0.75271068

H -1.61899847 3.30731505 0.30195439

H -1.01673146 3.26566688 -1.36358735

H -2.45543023 4.21134415 -0.96563024

**IM9a**

C -0.07770363 -0.05263131 0.69049631

C -1.37165433 0.51847280 1.04671334

C -2.55972418 -1.62779031 -1.58736394

C -2.45450429 -0.19127300 0.38767595

C -2.74291731 -0.20588996 -2.12651682

C -2.78091531 0.77139720 -0.91948107

H -3.52903014 -2.09901273 -1.38954728

H -3.66875340 -0.11134783 -2.70023314

H -2.02314734 -2.27878346 -2.28429864

H -1.92796371 0.06845279 -2.79983009

H -2.04989157 1.57761809 -1.05356859

C -0.34503917 -0.97360445 -0.50574633

H -0.00906324 -0.72648788 1.58164597

C -1.79780317 -1.46272694 -0.27062116

H -0.35825117 -0.31285741 -1.38141949

C 2.14667658 0.16792947 -0.40859300

C 1.19770895 0.81020681 0.61707145

C 0.79430077 -1.98416206 -0.71041559

C 2.16338544 -1.37849741 -0.29540690

H 1.66843360 0.88160165 1.60283763

H 0.93316235 1.82731538 0.30775957

H 1.71802620 0.37939705 -1.39747810

H 0.61773429 -2.89258603 -0.12593714

H 0.81541339 -2.28582816 -1.76320132

C 4.39629965 -0.69033996 -0.87501612

C 3.64406775 0.62166706 -0.50052630

C 3.31675347 -1.71134454 -1.25139013

H 5.12489519 -0.53261427 -1.67296735

H 4.95609519 -1.06149824 -0.01136248

H 3.72185502 1.31723607 -1.34640768

H 3.65818827 -2.74733434 -1.16619583

H 2.98353647 -1.55460453 -2.28474965

C 4.26350055 1.36463855 0.70221091

H 4.04768381 0.80281486 1.62407578

C 5.78666836 1.46406844 0.54971254

H 6.21335238 2.04947271 1.36824017

H 6.27997933 0.49073687 0.54717569

H 6.03849884 1.97229451 -0.38816551

C 3.72034427 2.79107692 0.84737778

H 3.95870447 3.37154898 -0.05070056

H 2.64058625 2.84047350 0.99534612

H 4.19015873 3.29382179 1.69704605

C 2.52655673 -1.84919041 1.12415249

H 2.70221813 -2.92947511 1.11954239

H 3.42958264 -1.36581904 1.50285754

H 1.73199844 -1.66241869 1.85472903

C -1.86226055 -2.71507606 0.59976507

H -2.89406717 -2.97810692 0.84101317

H -1.43804696 -3.56470162 0.05936012

H -1.30912339 -2.61314674 1.54038265

C -1.45919788 1.66170566 1.97089969

H -1.41724662 2.57065017 1.35028721

H -2.38321140 1.69646089 2.54654262

H -0.58512608 1.69748519 2.62537908

C -3.82006197 -0.31634009 1.09745235

C -4.88135211 0.18844042 0.10765112

C -4.18419805 1.33538554 -0.63013254

H -3.83404555 0.29263042 2.00558490

H -3.99820226 -1.34434292 1.41641342

H -5.15212730 -0.60684493 -0.59434516

H -5.79810249 0.50523016 0.61079871

H -4.65865217 1.53036834 -1.59822500

C -4.20286741 2.63805334 0.16786200

H -3.90719140 2.49362634 1.21033588

H -3.54591848 3.39227851 -0.27600907

H -5.21497137 3.04893429 0.18162385

**TS_9a-10a**

C 0.31546300 0.53528200 1.04835700

C 1.55934700 -0.21812900 1.50997600

C 2.90634400 2.02825500 -0.09254600

C 2.46512200 -0.73349600 0.67245100

C 3.29168300 0.87867300 -1.05360700

C 2.52458100 -0.43684200 -0.81139000

H 3.04932400 1.69302600 0.93600400

H 4.36195200 0.70887600 -0.90333600

H 3.56618000 2.87728400 -0.28361300

H 3.16934500 1.20081800 -2.09483800

H 1.52174200 -0.37720200 -1.25985100

C 0.34446100 1.77571900 0.13488900

H -0.11136800 0.98986500 1.95155500

C 1.49604100 2.50435800 -0.27143500

H 0.70983400 1.46644300 -0.98441100

C -1.77953300 0.10549700 -0.38171500

C -0.72229100 -0.51375300 0.52503100

C -1.03513800 2.45235900 0.00886500

C -2.22545800 1.48055300 0.14724200

H -1.17486100 -0.99182700 1.40019400

H -0.16696300 -1.29048500 -0.00747100

H -1.27541400 0.32523000 -1.34015700

H -1.07404100 3.24508900 0.76726000

H -1.11355900 2.94102100 -0.96482300

C -4.15037700 0.46613500 -0.87853500

C -3.08339800 -0.66529800 -0.77398600

C -3.38433300 1.79435000 -0.81311800

H -4.75699500 0.37931000 -1.78227200

H -4.84326200 0.40925100 -0.03446400

H -2.92762500 -1.09081900 -1.77381400

H -4.00679800 2.63119500 -0.48247700

H -2.98005400 2.04784600 -1.80153600

C -3.51916400 -1.84642500 0.11823100

H -3.49805900 -1.52673800 1.17136500

C -4.94878000 -2.28591700 -0.22189300

H -5.22245900 -3.16883800 0.36128300

H -5.69434700 -1.51452500 -0.02131400

H -5.01817000 -2.55476100 -1.28223400

C -2.60488100 -3.06486000 -0.05430800

H -2.65485300 -3.42052200 -1.08952500

H -1.55774300 -2.86700900 0.17804700

H -2.93563600 -3.88385700 0.58991700

C -2.76300100 1.44272100 1.58718000

H -3.12342600 2.43632100 1.87172500

H -3.60235200 0.75088200 1.67909500

H -2.01945400 1.13888700 2.32763700

C 1.38608300 3.82280600 -0.96981000

H 2.12378200 3.88886500 -1.77297500

H 0.39879900 4.08491400 -1.33700200

H 1.67452100 4.56933000 -0.21635300

C 1.56732800 -0.50236100 2.99036400

H 1.67702600 0.42398100 3.56631500

H 0.62282400 -0.96135300 3.30554000

H 2.37774100 -1.17391100 3.27399000

C 3.60788400 -1.67096800 1.03190400

C 4.32720500 -1.95510700 -0.30332800

C 3.28186900 -1.64951300 -1.39466000

H 3.20513300 -2.59205300 1.46754900

H 4.28248600 -1.24257100 1.77968900

H 5.19898600 -1.30280600 -0.41211500

H 4.69230100 -2.98244700 -0.36978300

H 3.76072900 -1.39373500 -2.34664400

C 2.32852800 -2.82528600 -1.60426700

H 1.83057300 -3.09672500 -0.66578400

H 1.55329900 -2.58308400 -2.33753600

H 2.86735900 -3.70568900 -1.96218600

**IM10a**

C 0.34111928 0.52287619 1.02916934

C 1.59206149 -0.26504035 1.40140278

C 3.00542403 2.17457070 0.04234839

C 2.48161258 -0.63536803 0.47407571

C 3.35926349 1.15651815 -1.03712388

C 2.52093512 -0.13432283 -0.94880146

H 3.24904296 1.78223486 1.03234535

H 4.42054456 0.90606486 -0.93757723

H 3.59944440 3.08358969 -0.09607244

H 3.23691037 1.59488220 -2.03489560

H 1.50354341 0.07029067 -1.32917400

C 0.43799779 1.60956042 0.03969158

H 0.14128715 1.21710992 1.88461842

C 1.53592421 2.61824034 0.00519975

H 1.37923753 3.20388239 -0.90923322

C -1.70847295 -0.25650487 -0.31787517

C -0.88623853 -0.45180671 0.94529903

C -0.70565146 1.93704089 -0.85168911

C -2.01744404 1.23335345 -0.54393373

H -1.50823499 -0.32892642 1.83782124

H -0.47527043 -1.46247167 0.97472856

H -1.05853662 -0.55132641 -1.15824412

H -0.79562931 3.03053273 -0.92163966

H -0.32736757 1.63696089 -1.84911061

C -3.93056083 0.01827962 -1.31127565

C -3.05995083 -1.01712124 -0.53670896

C -2.99213826 1.16038816 -1.72448594

H -4.44478171 -0.42840642 -2.16433993

H -4.70822836 0.41711120 -0.65239299

H -2.85097178 -1.86652852 -1.19945209

H -3.51840116 2.10047512 -1.91336058

H -2.44177883 0.89433822 -2.63595924

C -3.78596643 -1.61338992 0.68848814

H -3.90563129 -0.83018183 1.45282702

C -5.18208413 -2.10462778 0.28339117

H -5.68645615 -2.56167649 1.13824076

H -5.82543870 -1.30667566 -0.09165873

H -5.09932192 -2.86719845 -0.49959488

C -3.03696827 -2.80050362 1.30521772

H -2.86564335 -3.57130992 0.54560281

H -2.07259621 -2.53802418 1.74079367

H -3.63679833 -3.25209667 2.09970072

C -2.67687047 1.91217221 0.66597913

H -2.98479084 2.92927306 0.40390504

H -3.56478029 1.37190770 0.99940505

H -2.00244879 1.98497721 1.52700376

C 1.22834913 3.56632232 1.20564796

H 1.86434707 4.44739608 1.10334820

H 0.18640958 3.89754572 1.22982812

H 1.47159851 3.06841105 2.14781183

C 1.61475789 -0.74288859 2.82783774

H 1.77423728 0.09498156 3.51691086

H 0.66525902 -1.21286310 3.10876168

H 2.41221811 -1.46679906 2.99566594

C 3.61262298 -1.63329520 0.66594920

C 4.22365908 -1.82193103 -0.74169723

C 3.14934359 -1.30939614 -1.72394778

H 3.21416634 -2.57439663 1.05922722

H 4.35293239 -1.27995187 1.39088390

H 5.14197386 -1.23657650 -0.83875844

H 4.48902599 -2.86249459 -0.94143073

H 3.59966620 -0.96230484 -2.66026389

C 2.10318425 -2.38159924 -2.02465603

H 1.62338352 -2.72833576 -1.10138860

H 1.32013346 -1.99769832 -2.68612128

H 2.55792798 -3.24804373 -2.51040867

**TS_10a-PD**

C 0.33842400 0.28157800 0.67320300

C 1.48356500 -0.43473600 1.30903600

C 3.06467000 2.00420800 0.14665000

C 2.51734400 -0.79679100 0.53469300

C 3.52530400 0.99504000 -0.90136700

C 2.75138100 -0.34282000 -0.88941300

H 3.23707400 1.61204200 1.15262800

H 4.58759300 0.79491300 -0.72634800

H 3.68356000 2.90381900 0.06004000

H 3.45735700 1.42921300 -1.90562000

H 1.80512200 -0.21723400 -1.43430500

C 0.45291200 1.50989800 -0.03331000

H 0.04607500 1.30242100 1.30794400

C 1.61612600 2.49217100 0.02255800

H 1.53016800 3.03839900 -0.92637900

C -1.87143900 -0.13419200 -0.52065500

C -0.94640600 -0.56675400 0.60473200

C -0.71292800 2.02800600 -0.84398100

C -2.05927900 1.39284900 -0.51832500

H -1.44344500 -0.54045400 1.58206600

H -0.59609400 -1.59242700 0.46098500

H -1.33192100 -0.35428700 -1.45463900

H -0.73738600 3.12186900 -0.78074200

H -0.42392000 1.79199700 -1.88162300

C -4.12335200 0.43494300 -1.27676200

C -3.29597300 -0.75317900 -0.69590800

C -3.12243100 1.55177700 -1.61220100

H -4.71919800 0.13795600 -2.14180400

H -4.82870100 0.80125000 -0.52509400

H -3.22013100 -1.53657800 -1.46050400

H -3.58119100 2.54446300 -1.62955300

H -2.66362200 1.37720600 -2.59314400

C -3.96450600 -1.42459700 0.52300000

H -3.93283200 -0.73328100 1.37931300

C -5.43403500 -1.73976300 0.21460600

H -5.89665200 -2.25720500 1.05858200

H -6.02948700 -0.84905700 0.00708800

H -5.50107400 -2.40065200 -0.65714400

C -3.28285400 -2.73990000 0.91942100

H -3.28235900 -3.43179900 0.06997300

H -2.25160200 -2.62406000 1.25559700

H -3.83372300 -3.22021400 1.73223500

C -2.58658900 1.94928800 0.81383800

H -2.77035900 3.02413500 0.72186400

H -3.52640100 1.47628500 1.10353000

H -1.89644400 1.80885500 1.65514700

C 1.30540300 3.49428000 1.15848200

H 2.00233200 4.33248200 1.10636100

H 0.28929200 3.89519500 1.10341700

H 1.43895500 3.00634300 2.13041000

C 1.26240800 -0.92907800 2.71577200

H 0.45482200 -1.66840800 2.76475200

H 2.16494800 -1.39702000 3.10891500

H 0.99243000 -0.10785400 3.38895300

C 3.62484100 -1.75294400 0.93767300

C 4.49327700 -1.91338800 -0.32805300

C 3.57607600 -1.49705600 -1.49562000

H 3.18963500 -2.70830400 1.25120900

H 4.19668400 -1.37591200 1.79202200

H 5.36664300 -1.25794600 -0.27160900

H 4.86733400 -2.93255900 -0.44728600

H 4.16012000 -1.14428700 -2.35260100

C 2.66962800 -2.64582300 -1.93561300

H 2.06121200 -3.00949400 -1.09860800

H 1.98853400 -2.33032900 -2.73139000

H 3.25723500 -3.48759800 -2.30936700

**PD**

C 0.32032468 0.10833016 0.89095381

C 1.32940633 -0.85945347 1.09714852

C 2.91277313 1.94205394 0.91629088

C 2.46278067 -0.93353527 0.29723901

C 3.76360706 1.09292901 -0.02571518

C 2.93186590 0.00789517 -0.75836580

H 2.67792722 1.40190331 1.84033786

H 4.60260247 0.63485647 0.50888484

H 3.50247471 2.81173854 1.22147307

H 4.19402207 1.73005557 -0.80650350

H 2.11142327 0.49281284 -1.29072691

C 0.37035369 1.50796663 0.33406461

H -0.27770089 2.01740299 1.06373107

C 1.62855227 2.42587734 0.23119905

H 1.85523804 2.54803682 -0.83664380

C -1.83203865 -0.31572752 -0.13981632

C -1.08997011 -0.29732882 1.22273116

C -0.45566824 1.55614864 -0.99862715

C -1.88885178 1.07245267 -0.79627530

H -1.56127325 0.42507097 1.89781329

H -1.13738339 -1.28139274 1.67998462

H -1.18822583 -0.90967110 -0.80935478

H -0.42808647 2.59272393 -1.35189231

H 0.04787995 0.94148832 -1.75669985

C -3.76429383 -0.23971755 -1.58598321

C -3.23947655 -0.95880633 -0.29749346

C -2.64906981 0.70647469 -2.08124690

H -4.06916861 -0.94978368 -2.35719099

H -4.65366722 0.34646626 -1.33617627

H -3.09075073 -2.02611090 -0.50723748

H -3.04483339 1.57986175 -2.60678805

H -1.97366507 0.18343668 -2.76863663

C -4.26106961 -0.90651053 0.85962056

H -4.48275774 0.14167052 1.10110200

C -5.56169242 -1.57918984 0.40030868

H -6.32107606 -1.51755791 1.18353125

H -5.97828655 -1.12839844 -0.50274961

H -5.38162446 -2.64043331 0.19336162

C -3.78940537 -1.61072435 2.13808103

H -3.37160824 -2.59775245 1.90527360

H -3.04522534 -1.04369341 2.69944584

H -4.63615964 -1.76931999 2.81088661

C -2.69499659 2.11402435 -0.00589189

H -2.62844280 3.08411879 -0.50844184

H -3.75188693 1.84930612 0.04818202

H -2.34720779 2.25397573 1.02269532

C 1.22144611 3.80526054 0.76320212

H 1.99104809 4.54739559 0.53973388

H 0.28296584 4.15020864 0.31697805

H 1.08726713 3.77281821 1.84972703

C 1.09398468 -1.98639561 2.09033292

H 0.62816551 -2.86000190 1.62174879

H 2.04212769 -2.31048793 2.51961613

H 0.46346427 -1.66730341 2.91974562

C 3.40673019 -2.10229309 0.35210959

C 4.46146249 -1.85596351 -0.73909964

C 3.78156178 -0.86702872 -1.70256016

H 2.82502708 -3.01785196 0.17202952

H 3.82770740 -2.22234020 1.35710000

H 5.36134977 -1.41702717 -0.30108767

H 4.76034256 -2.78021581 -1.23714777

H 4.51783460 -0.24893105 -2.22559808

C 2.89807194 -1.58150665 -2.72481346

H 2.14259891 -2.20609841 -2.23210104

H 2.37199772 -0.86450476 -3.36154507

H 3.49455527 -2.23005050 -3.37033760

**TS_4-5b**

C -0.15980900 0.00812000 -1.33499200

C -1.33133800 -0.76783600 -1.61390100

C -3.18905900 2.01205400 -0.21763800

C -1.58790700 -1.84064700 -0.81423700

C -3.76467900 1.13139500 0.89956100

C -3.69248500 -0.32300900 0.54428000

H -3.58145000 1.68353200 -1.19021300

H -4.81420700 1.42248200 1.02947700

H -3.57854700 3.03384000 -0.10543000

H -3.26257600 1.35103800 1.84658300

H -0.86146800 -2.06277900 -0.03145100

C -0.77466300 1.47378400 0.41924700

H -0.03191400 0.89551600 -1.94854000

C -1.69417800 2.19253100 -0.30120600

H -1.15885600 0.65138200 1.02406100

C 1.80861500 -0.04605300 0.42431200

C 1.10792900 -0.61277600 -0.81797700

C 0.53425000 2.09845000 0.88904600

C 1.87931300 1.49091800 0.46155900

H 1.79131400 -0.51523300 -1.67735800

H 0.94694300 -1.67830700 -0.67359300

H 1.19199500 -0.34531400 1.28503900

H 0.53571300 3.16830300 0.65763900

H 0.47521000 2.02960100 1.98274800

C 4.03018100 0.68901900 1.19293500

C 3.26847300 -0.57435700 0.70658900

C 2.95391400 1.71329600 1.54009800

H 4.69412000 0.47202200 2.03220400

H 4.66078900 1.07782100 0.38643800

H 3.19413900 -1.27761500 1.54644000

H 3.32341200 2.74285400 1.56523300

H 2.52671500 1.48941100 2.52536400

C 4.04513500 -1.32554000 -0.39986100

H 4.04314400 -0.71321000 -1.31572000

C 5.50593100 -1.51635300 0.03458300

H 6.05821000 -2.07003600 -0.72846400

H 6.02910800 -0.57402100 0.20268000

H 5.54901400 -2.09736900 0.96310200

C 3.48267600 -2.71736600 -0.72103400

H 3.35922700 -3.29564700 0.20157800

H 2.52740900 -2.70735100 -1.24604800

H 4.18268400 -3.26572600 -1.35673200

C 2.36192800 2.09002600 -0.86819100

H 2.56477800 3.15837700 -0.74353000

H 3.28330900 1.61741800 -1.21884100

H 1.62715900 1.99436000 -1.67308800

C -1.27796800 3.36586500 -1.14158700

H -1.90658800 3.44118700 -2.03431200

H -1.42708400 4.29535300 -0.57673900

H -0.23002900 3.32602400 -1.44315600

C -2.23056800 -0.29357500 -2.72731800

H -1.89900300 -0.70932100 -3.68364200

H -3.27308300 -0.58400000 -2.58630700

H -2.18660500 0.79598400 -2.81749000

C -2.79161700 -2.71843800 -0.76512700

C -3.28360600 -2.76041100 0.69743100

C -3.40806200 -1.37742100 1.31730900

H -2.50786600 -3.73192400 -1.07629200

H -3.57079100 -2.38532200 -1.45379200

H -4.23107100 -3.30665000 0.76139600

H -2.56221000 -3.33904700 1.28750800

H -3.90949500 -0.51414200 -0.50518500

C -3.16777800 -1.31203300 2.79883300

H -2.14472400 -1.62394700 3.04116800

H -3.33853700 -0.31858100 3.21454500

H -3.83605600 -2.00882800 3.31642900

**IM5b**

C -0.14901894 0.09346175 -1.31042590

C -1.32934314 -0.79715407 -1.69647364

C -2.80451850 2.30004838 0.44911336

C -1.67870998 -1.82236410 -0.91405430

C -3.27113696 1.23513935 1.44657138

C -3.51743684 -0.09508927 0.76853163

H -3.62205843 2.58602373 -0.23082429

H -4.17539664 1.62471685 1.92853277

H -2.55420209 3.24862340 0.95483076

H -2.52802514 1.12847167 2.24500192

H -1.00594509 -2.10740804 -0.10138025

C -0.70224521 0.95329633 -0.13290566

H 0.07069444 0.75320347 -2.15459391

C -1.64429665 1.99562940 -0.42988102

H -1.08395620 0.27818415 0.63104070

C 1.79577640 -0.12179811 0.35992959

C 1.15135294 -0.66113924 -0.91775006

C 0.35460671 1.93723038 0.63309188

C 1.78342862 1.42064118 0.39517307

H 1.85070130 -0.58870447 -1.75664708

H 0.92029654 -1.72047698 -0.80029547

H 1.15483920 -0.43490645 1.19900111

H 0.28736399 2.98945140 0.33403193

H 0.07641500 1.87768011 1.68866867

C 3.85994231 0.74400485 1.38034351

C 3.25349645 -0.54766243 0.75837785

C 2.68762623 1.70473950 1.60742559

H 4.41476648 0.54242907 2.29899188

H 4.56770550 1.20104308 0.68196593

H 3.16545139 -1.30385117 1.54911391

H 2.99340318 2.75268904 1.67977608

H 2.15566105 1.44294688 2.53011961

C 4.16424913 -1.17598444 -0.31823279

H 4.16196851 -0.53145252 -1.21091630

C 5.60643984 -1.27408978 0.19671992

H 6.24032707 -1.76786825 -0.54398243

H 6.05163047 -0.30194574 0.41621086

H 5.63862367 -1.87461314 1.11316711

C 3.71889746 -2.58687680 -0.71880031

H 3.70884055 -3.23783104 0.16261014

H 2.73007422 -2.62502062 -1.17514300

H 4.42449885 -3.01679340 -1.43466517

C 2.37832811 2.05557640 -0.86891397

H 2.48368328 3.13705036 -0.73378076

H 3.36816317 1.65067081 -1.08941040

H 1.76327299 1.88610196 -1.75679897

C -1.45829767 2.88477076 -1.60404872

H -2.10282749 2.48681033 -2.40065400

H -1.80528713 3.90009407 -1.39601627

H -0.43452187 2.89381171 -1.97757054

C -2.21450281 -0.30117028 -2.81485671

H -1.63553112 0.23319241 -3.57385973

H -2.72916867 -1.12304881 -3.31441885

H -2.99900140 0.38248409 -2.45248066

C -3.01200810 -2.51879209 -0.88761208

C -3.53079880 -2.59142447 0.56326953

C -3.47497343 -1.29598173 1.35346005

H -2.93719102 -3.54212421 -1.27209271

H -3.73270395 -1.99692451 -1.52430489

H -4.55184741 -2.99111379 0.57301367

H -2.92442486 -3.32644201 1.10818751

H -3.67077551 -0.04904080 -0.31033183

C -3.31377623 -1.47898394 2.83885425

H -2.32571120 -1.89422812 3.07151762

H -3.44698570 -0.55558806 3.40422624

H -4.04905164 -2.20206640 3.20650274

**TS_5b-6b**

C -0.22474655 -0.26825301 -1.11200678

C -1.17033557 -1.45781062 -0.99196738

C -2.82020855 1.37443305 -1.68585479

C -1.58118681 -1.87000528 0.21156355

C -3.94561309 1.22955507 -0.58033162

C -3.96832802 -0.15667736 -0.02436171

H -2.70754988 0.38392137 -2.14257404

H -4.87647217 1.45676151 -1.10916081

H -3.14406938 2.07275221 -2.45935252

H -3.82279106 1.99560803 0.18986531

H -1.16877786 -1.35433062 1.08415219

C -0.80203658 0.97294983 -0.17639316

H -0.26062753 0.06314972 -2.15789403

C -1.51148508 1.79808663 -1.14220499

H -1.58061578 0.47372446 0.40538640

C 1.83124508 -0.15115839 0.47104449

C 1.26370911 -0.66883708 -0.83934968

C 0.17699473 1.68256502 0.79175424

C 1.65622901 1.37502806 0.57697867

H 1.88727207 -0.32839073 -1.67146961

H 1.27739163 -1.76059402 -0.85321533

H 1.22023543 -0.57819404 1.28192913

H 0.00095768 2.76502901 0.79315284

H -0.09240465 1.33791737 1.79673032

C 3.78244342 0.85346345 1.57083112

C 3.32349305 -0.45050690 0.85222633

C 2.51999825 1.69033165 1.80512031

H 4.32545015 0.64851173 2.49570668

H 4.46918819 1.40934791 0.92373986

H 3.31117279 -1.27001561 1.58242494

H 2.72877793 2.75798238 1.92472269

H 1.99906244 1.35005246 2.70869997

C 4.32249287 -0.89029853 -0.24217690

H 4.32807017 -0.13927814 -1.04692492

C 5.73519624 -0.96001569 0.35455400

H 6.45429079 -1.27529276 -0.40550007

H 6.07725476 -0.00543511 0.75840697

H 5.76338723 -1.69788792 1.16479997

C 4.00711543 -2.26374532 -0.84833068

H 3.84040536 -3.00071462 -0.05451443

H 3.13637420 -2.26675146 -1.50360162

H 4.85430813 -2.61327622 -1.44448436

C 2.19745557 2.11078956 -0.65606647

H 2.16570220 3.19440634 -0.49464854

H 3.23538248 1.84297564 -0.86630343

H 1.62447808 1.88440007 -1.56280703

C -1.00785434 3.10432099 -1.59984419

H 0.00949440 3.33342776 -1.29643980

H -1.13996390 3.21206324 -2.68135759

H -1.68783300 3.85179116 -1.15392897

C -1.54152206 -2.11916750 -2.29267537

H -0.63490077 -2.47452092 -2.79703684

H -2.19141369 -2.98272143 -2.14997960

H -2.02994657 -1.43022272 -2.99308452

C -2.72612055 -2.77983129 0.55730779

C -3.64484943 -2.03558281 1.56260733

C -3.83648572 -0.55753264 1.24715555

H -2.38570267 -3.71095110 1.02170734

H -3.28262394 -3.06180557 -0.34041301

H -4.61336369 -2.54259858 1.63328299

H -3.19498221 -2.10731663 2.55935692

H -4.01202044 -0.93104139 -0.79104545

C -3.77739959 0.36324374 2.43349449

H -2.79633434 0.29695471 2.92084066

H -3.97897656 1.40721187 2.19064359

H -4.51298484 0.04974496 3.18179083

**IM6b**

C -0.07661859 -0.18567191 -1.08415004

C -1.04272142 -1.34168292 -0.74536476

C -2.70500401 1.26381635 -1.71574995

C -1.51790744 -1.51955828 0.49162698

C -4.15825213 0.90339414 -1.31265993

C -4.10834327 -0.32631052 -0.45519716

H -2.29023985 0.42460084 -2.27732136

H -4.73069453 0.72473147 -2.22740187

H -2.69431866 2.13550350 -2.37865784

H -4.62885311 1.74786434 -0.80255548

H -1.18838765 -0.83857035 1.28255555

C -0.48066884 1.06379273 -0.36580972

H -0.29210051 0.05732468 -2.13835334

C -1.84952872 1.58882682 -0.46982586

H -2.27435329 0.89397098 0.30709738

C 2.01884583 -0.21214226 0.33255746

C 1.40252136 -0.65468348 -0.98174786

C 0.44259575 1.71280699 0.59562797

C 1.91086671 1.31574146 0.48036329

H 1.98018636 -0.27102250 -1.82869285

H 1.39498312 -1.74305823 -1.06595263

H 1.38356891 -0.63500947 1.12903633

H 0.29982148 2.79896891 0.58384452

H 0.03674976 1.38810202 1.57729392

C 3.96489219 0.67025401 1.52858882

C 3.48851989 -0.57806303 0.72402889

C 2.72721371 1.54918181 1.75632086

H 4.45882352 0.39914525 2.46354077

H 4.69810800 1.23045477 0.93885575

H 3.43694454 -1.43982019 1.40162054

H 2.97442876 2.60072859 1.92887761

H 2.15789855 1.19162672 2.62388832

C 4.49590008 -0.97961727 -0.37628186

H 4.56185693 -0.17010674 -1.11839326

C 5.88355530 -1.16538638 0.25239000

H 6.61339686 -1.44460519 -0.51130719

H 6.25177041 -0.26560648 0.74902840

H 5.85383348 -1.97164124 0.99446067

C 4.12807472 -2.28128094 -1.09925182

H 3.88401391 -3.06631420 -0.37440064

H 3.28878930 -2.17798889 -1.78713216

H 4.97829544 -2.63502240 -1.68826729

C 2.51204096 2.08574235 -0.70527655

H 2.54401655 3.15636800 -0.48010355

H 3.52969933 1.76118052 -0.92951043

H 1.91815742 1.96191999 -1.61879092

C -2.08008072 3.02001317 0.02769535

H -1.81528271 3.15527333 1.07754825

H -1.51898573 3.74095813 -0.57413556

H -3.13964078 3.26133241 -0.07174102

C -1.34895888 -2.22395477 -1.92527053

H -0.42223164 -2.49419352 -2.44314043

H -1.84196374 -3.15012493 -1.63254600

H -1.98496054 -1.71297922 -2.65883575

C -2.61100688 -2.45989954 0.92300865

C -3.74133711 -1.66278358 1.61566687

C -4.17363811 -0.40130656 0.88291944

H -2.22854394 -3.20186191 1.63171146

H -3.00502583 -3.01581482 0.06962531

H -4.60014077 -2.32050888 1.79257334

H -3.39186315 -1.36247989 2.61157539

H -3.81259356 -1.22497268 -0.99591685

C -4.61898222 0.72350643 1.78195871

H -3.79914699 1.06000469 2.42926367

H -5.00854524 1.58488964 1.23822688

H -5.41006870 0.36822412 2.45072110

**TS_6b-7b**

C -0.03883400 -0.40042900 -1.07289200

C -0.95161000 -1.51263800 -0.44315700

C -2.72199200 0.70417800 -1.94092000

C -1.44728300 -1.44226200 0.79707800

C -4.17519700 0.45254800 -1.42473700

C -4.06679600 -0.57459800 -0.32334400

H -2.30455800 -0.26370400 -2.22100500

H -4.78613300 0.07792500 -2.25203300

H -2.73178300 1.33740900 -2.83477100

H -4.61791800 1.39228600 -1.08032000

H -1.18259200 -0.61227300 1.44913400

C -0.55174300 0.86385800 -0.48764600

H -0.31594800 -0.40095700 -2.13708200

C -1.89699400 1.39798400 -0.82929800

H -2.44083000 1.20050000 0.12573100

C 2.02480800 -0.19010700 0.38136300

C 1.47000300 -0.75316000 -0.92398400

C 0.21402300 1.53289900 0.60464000

C 1.73015200 1.32817600 0.46387000

H 2.02789000 -0.35938700 -1.78018800

H 1.54203100 -1.84324900 -0.94639600

H 1.46424500 -0.65944500 1.20566600

H -0.05809100 2.58740400 0.70148800

H -0.11151300 1.03226600 1.53833600

C 3.89115200 1.01808200 1.46636400

C 3.55353800 -0.33804400 0.73882900

C 2.53759300 1.73311400 1.71048500

H 4.44639400 0.85682700 2.39250100

H 4.50694600 1.64152300 0.81107500

H 3.65349300 -1.16164600 1.45670900

H 2.64533100 2.81635500 1.81836600

H 2.05110500 1.33077200 2.60831100

C 4.54531000 -0.64354100 -0.41433400

H 4.39367500 0.07445200 -1.23194000

C 5.99718000 -0.51110100 0.10535100

H 6.70280300 -0.78313800 -0.68547800

H 6.23583700 0.50215800 0.43665500

H 6.14768400 -1.19749900 0.94737200

C 4.39028300 -2.08411700 -0.95112000

H 4.49820000 -2.79388700 -0.12255500

H 3.43256100 -2.26417800 -1.44014800

H 5.17926300 -2.29536300 -1.67994400

C 2.20192900 2.11065600 -0.77786400

H 2.09152100 3.18642900 -0.59975700

H 3.25029400 1.90480300 -1.00447100

H 1.60510900 1.85431100 -1.66446400

C -1.91351300 2.94642400 -1.03731000

H -1.62224000 3.50039300 -0.14485500

H -1.26309200 3.22212900 -1.87190900

H -2.93886100 3.23099200 -1.28868100

C -1.22779000 -2.63501500 -1.42237100

H -0.29208400 -2.95419700 -1.89610000

H -1.65954200 -3.50192100 -0.92020200

H -1.91282500 -2.31362700 -2.21702300

C -2.49297100 -2.34943300 1.40956800

C -3.64437900 -1.45879900 1.97255100

C -4.09522100 -0.36660300 0.99756200

H -2.06579300 -2.93156000 2.23431900

H -2.88475800 -3.05565400 0.67460100

H -4.49190500 -2.09050000 2.26343800

H -3.28456400 -0.96910700 2.88653400

H -3.78310600 -1.56294500 -0.68342100

C -4.52912100 0.92549100 1.66490800

H -3.71063500 1.35415600 2.25885300

H -4.88010100 1.67357900 0.95121300

H -5.35068900 0.71619200 2.36088000

**IM7b**

C 0.24610155 0.23258456 1.24207353

C 1.21594398 -0.97806244 1.13200936

C 2.92379776 1.93622841 0.98865274

C 1.44476282 -1.61990802 -0.02145359

C 4.14316376 1.40774502 0.20321598

C 3.95715390 -0.02831763 -0.17633734

H 2.72098700 1.29234185 1.84986519

H 5.02108341 1.51376543 0.84991405

H 3.17492522 2.91855147 1.39952655

H 4.31552071 2.04991137 -0.66569098

H 0.88992577 -1.33287130 -0.91031687

C 0.60033061 1.08131311 0.07946668

H 0.57275721 0.76264701 2.14523440

C 1.66638110 2.10321178 0.11703039

H 1.96323844 2.31500016 -0.91828191

C -1.84357811 -0.48677285 0.02704098

C -1.23518426 -0.20058337 1.39020523

C -0.22082582 1.02782522 -1.15829463

C -1.70882858 0.73058451 -0.90920938

H -1.79309359 0.57951297 1.91816440

H -1.24236112 -1.08929314 2.02651994

H -1.22250291 -1.27456015 -0.42381559

H -0.05883991 1.93887480 -1.74709016

H 0.22642283 0.22066047 -1.76304824

C -3.72313472 -0.41343239 -1.53437563

C -3.31188563 -0.99490601 -0.14500727

C -2.46700475 0.23125344 -2.14390440

H -4.15516470 -1.17165917 -2.19013768

H -4.49140220 0.35448222 -1.40052552

H -3.26742446 -2.08919050 -0.21520525

H -2.70086401 1.02413284 -2.86041890

H -1.86096054 -0.52152524 -2.66363848

C -4.35843085 -0.69900435 0.95188014

H -4.42190609 0.38758589 1.11018810

C -5.73380446 -1.19775928 0.48838394

H -6.49029879 -0.99060200 1.24909327

H -6.06567033 -0.73506938 -0.44310705

H -5.70612785 -2.28261156 0.33403648

C -4.04139032 -1.37312259 2.29242227

H -3.81551490 -2.43509925 2.14237321

H -3.20605192 -0.91853984 2.82520282

H -4.90922990 -1.31544065 2.95458135

C -2.36506019 2.00011000 -0.34964533

H -2.26864828 2.82267865 -1.06685824

H -3.42922918 1.85357091 -0.15932540

H -1.90810588 2.32435459 0.59208978

C 0.84042303 3.35427094 0.60225075

H 0.53932496 3.23662032 1.64635130

H 1.51203832 4.21123692 0.52714363

H -0.04085094 3.55590717 -0.00830279

C 1.86847349 -1.32884073 2.44298636

H 2.37966338 -2.28997083 2.39331748

H 2.59280352 -0.57375204 2.76719191

H 1.10463955 -1.39812735 3.22520729

C 2.54193762 -2.59698244 -0.32693694

C 3.36265256 -2.03141511 -1.51332511

C 3.74772673 -0.56606991 -1.38198840

H 2.13334933 -3.56911281 -0.62124654

H 3.18223740 -2.76881327 0.54037274

H 4.25373229 -2.65150114 -1.66380528

H 2.76659214 -2.14349939 -2.42817876

C 3.81312104 0.18643326 -2.68360489

H 4.18110972 1.20720002 -2.57469574

H 4.47610107 -0.33165323 -3.38458381

H 2.82655182 0.22148748 -3.16388407

H 3.90403214 -0.69321854 0.68556134

**TS_7b-8b**

C 0.28207600 0.14691900 0.66705000

C 1.40094300 -0.58510900 1.35400400

C 3.09222400 1.97918200 0.18334400

C 1.79408300 -1.69916000 0.72228300

C 3.72541900 1.16222100 -0.97032900

C 3.98333200 -0.25075000 -0.53076100

H 3.27883800 1.48031300 1.13748100

H 4.66391800 1.65072200 -1.25556700

H 3.61463300 2.93849600 0.25705700

H 3.08582900 1.18910100 -1.85987200

H 1.25401500 -1.97930200 -0.18495100

C 0.44315300 1.40531900 0.01447500

H -0.00076900 1.18921200 1.30897600

C 1.61453100 2.38982800 0.06601600

H 1.51510900 2.91029100 -0.89584000

C -1.94968400 -0.14697900 -0.55175700

C -1.02555800 -0.65317300 0.54047700

C -0.71276300 1.97485600 -0.79010800

C -2.08088600 1.38258400 -0.48378800

H -1.50258200 -0.65515400 1.52913100

H -0.71880500 -1.68499800 0.36519200

H -1.43025900 -0.34576200 -1.50131600

H -0.70626400 3.06684700 -0.70508700

H -0.42950900 1.74988600 -1.83078400

C -4.18045300 0.53306500 -1.27262600

C -3.39759600 -0.70570100 -0.73590400

C -3.14002400 1.62618700 -1.56605300

H -4.78472200 0.29004300 -2.14860400

H -4.87416000 0.89624900 -0.50901300

H -3.35738700 -1.46580400 -1.52628300

H -3.56267800 2.63458700 -1.54010600

H -2.69016800 1.47647100 -2.55511800

C -4.08142900 -1.39154500 0.46627500

H -4.00603100 -0.73668800 1.34811100

C -5.56777100 -1.62935900 0.16984600

H -6.03903300 -2.16406900 0.99814000

H -6.12754100 -0.70587200 0.01249500

H -5.67702100 -2.24704600 -0.72887700

C -3.45320700 -2.74998800 0.79983600

H -3.51038400 -3.41160500 -0.07152800

H -2.40854400 -2.69400200 1.10819800

H -4.00291300 -3.23143800 1.61273700

C -2.58599900 1.90478200 0.87088300

H -2.71142300 2.99112700 0.82733800

H -3.55215900 1.47100300 1.13371200

H -1.91310100 1.68902800 1.71054000

C 1.28453600 3.43596700 1.16040700

H 1.48990500 3.03098600 2.15595400

H 1.91708000 4.31470400 1.02502600

H 0.24227700 3.76570600 1.13218900

C 1.96987700 -0.07032800 2.65149800

H 3.02978900 -0.31659200 2.74802200

H 1.87208400 1.01295000 2.75112800

H 1.44791800 -0.52317800 3.50059700

C 3.01177300 -2.54246200 0.96361800

C 3.77081300 -2.69482900 -0.38262300

C 3.76811900 -1.39645500 -1.17798800

H 2.72494400 -3.53267600 1.33453600

H 3.65204700 -2.09626100 1.72862800

H 4.78630200 -3.05710100 -0.19344300

H 3.27523000 -3.46982700 -0.97796500

C 3.37360600 -1.50094100 -2.62378300

H 3.41308900 -0.54306000 -3.14459000

H 4.04600600 -2.18909800 -3.14702800

H 2.36334900 -1.91751700 -2.72696100

H 4.29839600 -0.32546900 0.51199300

**IM8b**

C 0.44374875 0.45810193 0.83286504

C 1.34011575 -0.16504434 1.72820835

C 2.67691752 1.76197477 -0.83415670

C 1.60748683 -1.49173850 1.44844224

C 2.84095087 0.38818379 -1.53295384

C 3.21755319 -0.73027819 -0.59856598

H 3.19731462 1.75610120 0.13244298

H 3.63530285 0.49155290 -2.28256761

H 3.22255746 2.49403329 -1.43570041

H 1.93832075 0.13801854 -2.10341255

H 0.89911957 -2.04341095 0.83567275

C 0.46727761 1.93920171 0.66009999

H 1.02984944 2.38006202 1.48631162

C 1.26527392 2.36522025 -0.64459639

H 0.64524204 2.09742419 -1.51018381

C -1.65622889 0.49782006 -0.56611177

C -0.47468967 -0.33336317 -0.06459796

C -0.98520290 2.54838931 0.63360102

C -2.08227581 1.49205962 0.53259896

H -0.83071895 -1.21835034 0.47021026

H 0.14013374 -0.69709450 -0.90281981

H -1.29737303 1.11462855 -1.39965386

H -1.11806362 3.16897971 1.52539826

H -1.06846696 3.20621501 -0.23576113

C -4.09674397 0.69087209 -0.49083483

C -2.96350813 -0.20359551 -1.07159067

C -3.41904881 1.98854275 -0.03193465

H -4.89541968 0.86358819 -1.21507627

H -4.55727065 0.19976513 0.37264558

H -2.98063809 -0.11237761 -2.16535042

H -4.01343826 2.55030918 0.69390823

H -3.23095752 2.64474326 -0.89070125

C -3.14276080 -1.70474527 -0.77442866

H -2.94822233 -1.88529771 0.29476081

C -4.57908745 -2.15139310 -1.07176626

H -4.68096968 -3.23029129 -0.92866242

H -5.31452188 -1.66067114 -0.43192094

H -4.83499233 -1.92838699 -2.11369182

C -2.19739422 -2.57096227 -1.61543553

H -2.44686425 -2.46466014 -2.67659830

H -1.14223872 -2.30844248 -1.50427950

H -2.30722472 -3.62760573 -1.35636654

C -2.30413606 0.82028707 1.89782014

H -2.80710633 1.51907668 2.57264029

H -2.91983880 -0.07991843 1.82613767

H -1.36573371 0.53272135 2.38795761

C 1.43437055 3.89105469 -0.59301665

H 2.16092921 4.15684509 0.18247609

H 1.81962829 4.25392105 -1.54873927

H 0.50958248 4.42857152 -0.38262684

C 2.17747791 0.60086283 2.72456243

H 3.00380364 1.14419104 2.25532629

H 1.56493562 1.32576624 3.26507820

H 2.59836534 -0.07935449 3.46585373

C 2.82909968 -2.27625521 1.78255895

C 3.25260311 -2.96905119 0.46090991

C 2.99477796 -2.04610763 -0.72823049

H 2.59135824 -3.02576607 2.54914522

H 3.61616252 -1.63632086 2.18615901

H 4.29541862 -3.29536009 0.51605754

H 2.65009236 -3.87458072 0.33227369

C 2.41341568 -2.69138323 -1.95364069

H 2.25545381 -1.98380144 -2.76877616

H 3.09061279 -3.47213211 -2.31734539

H 1.46241116 -3.18988094 -1.72715790

H 3.72389345 -0.39468953 0.30794364

**TS_8b-9b**

C 0.35654277 0.63978006 0.73099508

C 1.44155097 0.22779392 1.44385240

C 2.31170017 1.96072480 -0.90524912

C 2.07346436 -1.02708291 1.00488244

C 2.57990483 0.49015958 -1.33144208

C 3.11628123 -0.44879528 -0.25696860

H 2.82140497 2.17831974 0.04123831

H 3.33655773 0.50676371 -2.12476172

H 2.80719798 2.59484815 -1.64609819

H 1.68650737 0.05438027 -1.79166484

H 1.36158110 -1.71554131 0.54601302

C 0.09037362 2.10563501 0.50879515

H 0.58446347 2.68731339 1.29095093

C 0.85222968 2.47611509 -0.81625510

H 0.28666633 2.05219471 -1.65904687

C -1.82410347 0.26664719 -0.52406844

C -0.51188599 -0.35352145 -0.04266078

C -1.41283180 2.50646656 0.45196311

C -2.34772891 1.29712328 0.49541791

H -0.72062234 -1.19963671 0.62316541

H 0.03045954 -0.76661956 -0.90173651

H -1.59470903 0.84942107 -1.42732096

H -1.64329542 3.19744902 1.26958383

H -1.60644094 3.04866756 -0.48060211

C -4.26808395 0.14413245 -0.36729054

C -3.04072127 -0.64790695 -0.90485332

C -3.75669695 1.55465699 -0.05190081

H -5.10188040 0.14136713 -1.07284346

H -4.63568782 -0.31827807 0.55458308

H -3.10242984 -0.67421913 -2.00118745

H -4.39946701 2.09692649 0.64767982

H -3.68697194 2.14922012 -0.97148931

C -3.02221101 -2.12249713 -0.45426017

H -2.78188096 -2.16641741 0.61924252

C -4.39756474 -2.76981000 -0.65931000

H -4.36427126 -3.83126278 -0.39924619

H -5.17766306 -2.30451670 -0.05466566

H -4.69657812 -2.69401925 -1.71111341

C -1.99809699 -2.95708803 -1.23318751

H -2.25139818 -2.95198471 -2.29917838

H -0.97242682 -2.59382397 -1.13683980

H -2.01487641 -3.99760369 -0.89645278

C -2.43231848 0.73612771 1.92445078

H -2.98565529 1.43441482 2.56003348

H -2.94703325 -0.22777031 1.96130661

H -1.44528999 0.59768164 2.37710094

C 0.88644546 4.00203390 -0.96231584

H 1.54234077 4.43663568 -0.19969586

H 1.27318973 4.29157603 -1.94266552

H -0.10043132 4.45300273 -0.84688785

C 2.23394756 1.05838615 2.41901293

H 2.38087661 0.49161092 3.34415112

H 3.23522046 1.32596234 2.05186193

H 1.72926831 1.98472053 2.68704623

C 3.12029770 -1.81912727 1.79039659

C 3.81821120 -2.58774054 0.65444117

C 3.57780083 -1.75398099 -0.56519214

H 2.67394780 -2.47955193 2.53559430

H 3.81289970 -1.14876648 2.30608567

H 4.88946981 -2.78317306 0.79307892

H 3.35593621 -3.56907631 0.48368680

C 3.55184835 -2.35574575 -1.90975474

H 3.87563417 -1.65784146 -2.68503123

H 4.12064200 -3.28605740 -1.95018960

H 2.50076104 -2.60661702 -2.13053153

H 3.81003215 0.05452884 0.43100594

**IM9b**

C 0.31854693 0.61107108 0.71722853

C 1.40053645 0.18526618 1.41098227

C 2.27889520 1.88034388 -0.95125617

C 2.05876892 -1.05818998 0.91549583

C 2.53924539 0.39883813 -1.35449195

C 3.04792137 -0.53281972 -0.24936063

H 2.79920532 2.11067853 -0.01327354

H 3.29326542 0.39849972 -2.14957286

H 2.77298956 2.49538797 -1.70930046

H 1.64026453 -0.04081767 -1.79962638

H 1.33568345 -1.72698923 0.44004490

C 0.07829601 2.08290184 0.48640466

H 0.59901395 2.66290477 1.25297352

C 0.82535747 2.41679523 -0.85467343

H 0.24488304 1.98375172 -1.68282155

C -1.88800275 0.27856621 -0.50667742

C -0.57435661 -0.36466284 -0.05523079

C -1.41641173 2.51387913 0.45476150

C -2.37391868 1.32244844 0.51779827

H -0.78157725 -1.21576082 0.60421434

H -0.05853132 -0.77551625 -0.93202276

H -1.66829942 0.85615964 -1.41583541

H -1.62099828 3.21090681 1.27430049

H -1.61795527 3.05755950 -0.47545003

C -4.33180827 0.20761352 -0.31085851

C -3.12960582 -0.61253040 -0.86291034

C -3.78572950 1.60779365 -0.00833658

H -5.17597764 0.21990708 -1.00401640

H -4.69558850 -0.24301736 0.61826165

H -3.20974005 -0.64410781 -1.95803267

H -4.40582736 2.16576682 0.69947507

H -3.71806901 2.19799373 -0.93095512

C -3.13366579 -2.08457487 -0.40400094

H -2.86956860 -2.12734240 0.66388803

C -4.52614206 -2.70458288 -0.57433586

H -4.50771499 -3.76590651 -0.31189475

H -5.28266422 -2.22237898 0.04685024

H -4.84831159 -2.62588776 -1.61911965

C -2.14429067 -2.94306676 -1.20107622

H -2.42775486 -2.94608521 -2.25951332

H -1.11222207 -2.59235944 -1.13800141

H -2.16640589 -3.97958640 -0.85221163

C -2.44926819 0.76810100 1.94991696

H -2.97442976 1.48232589 2.59194601

H -2.98859424 -0.18177489 1.99768333

H -1.45864879 0.60482208 2.38478078

C 0.88319824 3.93831948 -1.03694985

H 1.55494181 4.37957780 -0.29195384

H 1.26141944 4.20145222 -2.02811853

H -0.09489766 4.40719051 -0.91868672

C 2.20729852 0.99974825 2.38624378

H 2.35744765 0.42308006 3.30565565

H 3.21095198 1.26059572 2.01678226

H 1.71471517 1.92890004 2.66729107

C 3.03882771 -1.90171170 1.73626274

C 3.81908271 -2.62671539 0.62856041

C 3.80294132 -1.72526758 -0.54408033

H 2.54672386 -2.59465082 2.41900957

H 3.71087982 -1.26476178 2.32047001

H 4.82299006 -3.00190304 0.85940706

H 3.25799559 -3.51498137 0.27973875

C 4.38098248 -2.07933077 -1.84427016

H 5.02564507 -1.26477259 -2.19842003

H 4.92472681 -3.02299184 -1.83355473

H 3.55993519 -2.12670154 -2.57568751

H 3.79085562 0.01296689 0.38262496

**TS_9b-10b**

C 0.24522200 0.61314000 0.66084700

C 1.37266900 0.28546300 1.32326700

C 2.10058600 1.87471000 -1.11557800

C 2.09514700 -0.95640700 0.86255100

C 2.38298000 0.38114200 -1.46614000

C 2.93709300 -0.51271000 -0.36525200

H 2.63415000 2.16006900 -0.20144200

H 3.07074200 0.34169500 -2.31532800

H 2.56142900 2.46357500 -1.91436800

H 1.46352900 -0.10248900 -1.81161000

H 1.39174400 -1.70319100 0.47708500

C -0.07722300 2.05879900 0.35620600

H 0.41897100 2.70467400 1.08502900

C 0.63142300 2.36460300 -1.01078700

H 0.05344200 1.87440900 -1.80867400

C -1.97275000 0.09969400 -0.48156900

C -0.62352700 -0.45343600 -0.01782800

C -1.59106600 2.41397900 0.32386300

C -2.48847500 1.18559500 0.48292400

H -0.78105700 -1.25918200 0.70932200

H -0.11598800 -0.91327100 -0.87457900

H -1.79596000 0.62557100 -1.43099600

H -1.81883600 3.15396100 1.09844500

H -1.83281700 2.88408300 -0.63681400

C -4.40713200 -0.06759000 -0.23196400

C -3.17815400 -0.86764400 -0.75389600

C -3.92112800 1.37210400 -0.03097200

H -5.26281600 -0.13820300 -0.90731300

H -4.73378600 -0.47259800 0.73136700

H -3.27551200 -0.97516500 -1.84280300

H -4.55364600 1.94654200 0.65231300

H -3.89712500 1.90374100 -0.99068600

C -3.10966300 -2.30532200 -0.20019100

H -2.83095600 -2.26636000 0.86410800

C -4.47580500 -2.99434100 -0.30865500

H -4.40780100 -4.03391800 0.02335100

H -5.24557800 -2.50457100 0.28994000

H -4.81317100 -2.99980700 -1.35160800

C -2.09406700 -3.17196400 -0.95425900

H -2.38520800 -3.24999600 -2.00777400

H -1.07532500 -2.78093500 -0.92220500

H -2.07402800 -4.18552000 -0.54339100

C -2.51135100 0.72272000 1.94891000

H -3.05780800 1.45227800 2.55497800

H -3.00459100 -0.24573400 2.06862600

H -1.50611500 0.63326200 2.37121500

C 0.62803200 3.87716500 -1.26021000

H 1.29398300 4.37668500 -0.54739000

H 0.97641700 4.11324000 -2.26900400

H -0.36656200 4.31025700 -1.14144900

C 2.17472700 1.19036000 2.22258700

H 2.33137700 0.70042200 3.19033700

H 3.17630000 1.42508000 1.83047500

H 1.67660700 2.13673200 2.42342700

C 3.13792300 -1.66413300 1.73901600

C 4.10869800 -2.28354600 0.71748300

C 4.05709700 -1.37776700 -0.47058400

H 2.70095500 -2.40955000 2.40281900

H 3.68116500 -0.93987000 2.35496200

H 5.12258200 -2.48196800 1.07107000

H 3.70762200 -3.23761900 0.33462700

C 5.05004500 -1.43087600 -1.56614000

H 5.03445700 -0.54890900 -2.20571400

H 6.05604700 -1.61636700 -1.18570000

H 4.77286900 -2.29756300 -2.18323700

H 3.88249400 0.03408400 0.13681700

**IM10b**

C 0.24262356 0.66016680 0.69578053

C 1.37692015 0.36850498 1.35756970

C 2.10251091 1.91241347 -1.02853567

C 2.15826852 -0.83967386 0.89604064

C 2.40134906 0.40679921 -1.40547234

C 2.87153380 -0.52695323 -0.36530494

H 2.62417880 2.18612015 -0.10766582

H 3.09714710 0.37237146 -2.24504872

H 2.59204002 2.47970990 -1.82494988

H 1.45949787 -0.03797105 -1.76891690

H 1.46129577 -1.60921937 0.48642129

C -0.10409536 2.09901221 0.39153474

H 0.36716208 2.75222595 1.13050350

C 0.63368158 2.40303008 -0.96018973

H 0.07229716 1.91466115 -1.77054361

C -1.94110153 0.10027442 -0.48900068

C -0.59007238 -0.42483607 0.00360845

C -1.62144940 2.42598927 0.32049379

C -2.49637189 1.17892866 0.46248066

H -0.74585278 -1.23128164 0.73106109

H -0.06230040 -0.87801048 -0.84665063

H -1.75474387 0.62609547 -1.43666949

H -1.88205311 3.16215346 1.08798185

H -1.84784504 2.89056490 -0.64688090

C -4.37533022 -0.11459961 -0.28567918

C -3.12122302 -0.89208023 -0.78109047

C -3.92182947 1.33507559 -0.08082722

H -5.21574114 -0.20417734 -0.97751809

H -4.71281641 -0.52286517 0.67239876

H -3.19487734 -1.00428168 -1.87125032

H -4.57845678 1.89919667 0.58794947

H -3.88944944 1.86406810 -1.04173153

C -3.03360416 -2.32648502 -0.22134588

H -2.77498235 -2.27923635 0.84773739

C -4.38377346 -3.04262901 -0.35176545

H -4.30085640 -4.07950064 -0.01512354

H -5.17333538 -2.56661919 0.23184810

H -4.70244469 -3.05792664 -1.40040554

C -1.98838196 -3.17530333 -0.95506432

H -2.25990149 -3.26309165 -2.01293540

H -0.97746622 -2.76513547 -0.90828081

H -1.95438247 -4.18653329 -0.53990823

C -2.53861575 0.71837893 1.92882367

H -3.11342651 1.43647456 2.52194601

H -3.01233791 -0.26054317 2.04090291

H -1.54074055 0.65242283 2.37283872

C 0.64313916 3.91552956 -1.21123205

H 1.29389034 4.41492621 -0.48482594

H 1.00991900 4.15108379 -2.21337443

H -0.35475883 4.34626252 -1.11122655

C 2.15312980 1.26770738 2.28444893

H 2.32627160 0.74785598 3.23338203

H 3.14555333 1.53417090 1.89343042

H 1.62231642 2.18884406 2.51850064

C 3.18285129 -1.59987816 1.75966271

C 4.04166245 -2.31429335 0.71278068

C 4.12433409 -1.29086389 -0.43600442

H 2.69821145 -2.27306900 2.46674185

H 3.78815476 -0.88744974 2.32953378

H 5.02988124 -2.60126381 1.07338548

H 3.54207535 -3.21792990 0.34415430

C 4.65010573 -1.77494744 -1.78380693

H 4.80707958 -0.96152834 -2.49380932

H 5.60785853 -2.27519427 -1.63250044

H 3.95650421 -2.49723747 -2.22104837

H 4.77963501 -0.45715696 -0.06748225

**TS_10b-11b**

C 1.33039212 0.40443925 0.41229105

C 0.82049365 0.48294355 -2.48975927

C 1.69089651 -1.07451987 0.41118893

C 0.75907830 -1.04223961 -2.04389167

C 1.73032096 -1.47514394 -1.02466885

H 1.76719847 0.93866797 -2.18703595

H 0.85846752 -1.67743453 -2.92449072

H 0.84159864 0.44309328 -3.58174170

H -0.23984662 -1.20130787 -1.62000843

C 0.03400307 0.72935797 0.24389180

H 0.84430856 -1.67118585 0.80302710

C -0.33219276 1.92903675 -0.60291597

H 0.48206837 2.65659862 -0.57218230

C -0.35854291 1.39870121 -2.08101437

H -1.28983780 0.82933046 -2.21804674

C -2.47400059 0.44571783 0.58232857

C -1.09539386 -0.20916748 0.68743484

C -1.65772259 2.64691711 -0.22628530

C -2.39976591 1.94953677 0.91439565

H -0.89162084 -0.48681117 1.72966640

H -1.10954652 -1.14895569 0.12179539

H -2.76963745 0.40147614 -0.47656142

H -1.45708708 3.69169618 0.03294085

H -2.32104259 2.66444419 -1.09986326

C -4.46518690 1.14244995 1.83357690

C -3.69060423 -0.13207302 1.38836873

C -3.88982319 2.29859512 1.00859610

H -5.54523381 1.03109680 1.71504855

H -4.28445038 1.33604965 2.89583843

H -4.32014065 -0.68789420 0.68047603

H -4.07513389 3.27916052 1.45679103

H -4.32676430 2.30328832 0.00195417

C -3.41045233 -1.10745643 2.54989717

H -2.64114298 -0.67197989 3.20606245

C -4.67718351 -1.33009471 3.38612497

H -4.49275153 -2.06961502 4.16989417

H -5.03258633 -0.41790753 3.86803012

H -5.48493791 -1.71256857 2.75149235

C -2.93495022 -2.48041859 2.05996608

H -3.70647925 -2.93623939 1.42949275

H -2.01226684 -2.44478465 1.47762248

H -2.76418344 -3.15148586 2.90643145

C -1.70448588 2.23120772 2.25637995

H -1.84751191 3.28112252 2.53035421

H -2.10721295 1.61784861 3.06685804

H -0.62627972 2.04899291 2.21233868

C -0.36306221 2.59107797 -3.04636476

H 0.60144417 3.10968425 -3.01220098

H -0.54084528 2.27131624 -4.07597415

H -1.13671035 3.31449785 -2.78237406

C 2.52259323 1.31842230 0.27815620

H 3.11332186 1.28090407 1.19997358

H 3.20383093 1.03275384 -0.53587162

H 2.24081932 2.35845352 0.12654712

C 2.99755286 -1.62257426 1.01930039

C 3.34626577 -2.80531483 0.11075946

C 2.90058684 -2.31975686 -1.28416294

H 2.87427636 -1.89738436 2.06667644

H 3.78398855 -0.86425928 0.96235412

H 4.40296325 -3.07412433 0.12680477

H 2.76728224 -3.69743679 0.37473667

H 3.59809491 -1.48153483 -1.55244392

C 2.87318806 -3.33023538 -2.42487750

H 2.69516230 -2.86137269 -3.39403931

H 3.83594213 -3.84129410 -2.47298395

H 2.10055279 -4.08241262 -2.24741667

**IM11b**

C 1.25207286 -0.16318670 0.27828586

C 0.46134673 -0.00095041 -2.79513520

C 1.40922895 -1.66028999 0.50699328

C 0.59780228 -1.33804909 -2.04429755

C 1.58941318 -1.23170880 -0.90535267

H 1.45052816 0.46928986 -2.88394220

H 0.94924219 -2.10996341 -2.73097011

H 0.13476381 -0.20693769 -3.81895211

H -0.38076806 -1.68406588 -1.70373802

C -0.06980696 0.33292443 0.04449082

H 0.51188013 -2.19887328 0.79932590

C -0.24502531 1.56424379 -0.75099585

H 0.70846131 2.09416829 -0.82600894

C -0.53126560 1.03034711 -2.23375699

H -1.53432837 0.58372274 -2.22302607

C -2.53563173 0.47238974 0.55647238

C -1.28473657 -0.40630825 0.52487843

C -1.38234517 2.50865857 -0.25329319

C -2.14549407 1.91727829 0.93012102

H -1.03087613 -0.79609625 1.51913791

H -1.44396717 -1.29573040 -0.09557010

H -2.92581781 0.52723305 -0.46847237

H -0.94529573 3.47844387 0.00507254

H -2.09260331 2.68232332 -1.06685711

C -4.22694646 1.45640254 2.02900322

C -3.75143796 0.08439978 1.46699706

C -3.52626410 2.53112523 1.18767051

H -5.31456414 1.55086395 2.01414381

H -3.91855005 1.56000656 3.07416717

H -4.53732008 -0.31033281 0.81042583

H -3.48060390 3.50364804 1.68561632

H -4.04647018 2.66865045 0.23174281

C -3.53718577 -0.98770065 2.55433044

H -2.63615022 -0.73595615 3.13587059

C -4.72750908 -1.02279973 3.52097558

H -4.60325672 -1.82889362 4.24850793

H -4.85087976 -0.09144005 4.07620813

H -5.65512234 -1.21303143 2.96936986

C -3.37941932 -2.39207714 1.95940590

H -4.29424719 -2.67246272 1.42634418

H -2.54992053 -2.48769131 1.25537793

H -3.22192011 -3.12897226 2.75137579

C -1.28752034 1.98906635 2.20262230

H -1.17764441 3.03112256 2.51668228

H -1.72591340 1.43012212 3.03287486

H -0.27209181 1.59576735 2.05853519

C -0.52193495 2.24640334 -3.16898364

H 0.48006703 2.68478941 -3.20877327

H -0.79173161 1.93239501 -4.18017876

H -1.22194971 3.02479430 -2.86561377

C 2.30093086 0.86920866 0.69262386

H 1.81656610 1.78803333 1.02792825

H 2.86754031 0.48529927 1.54284119

H 3.00284264 1.12983375 -0.10239591

C 2.73955462 -2.13651884 1.05908724

C 3.81289734 -1.61739394 0.07576855

C 3.08953181 -1.25540179 -1.24881229

H 2.72536090 -3.22927381 1.07910218

H 2.90983017 -1.79974857 2.08551938

H 4.33768393 -0.75275012 0.48336036

H 4.57244947 -2.38066227 -0.10569026

H 3.41913946 -0.27547244 -1.61343347

C 3.39234434 -2.28302762 -2.34700581

H 2.97892450 -1.99480075 -3.31611650

H 4.47474950 -2.36533110 -2.46573090

H 3.00735410 -3.27338405 -2.08175244

**TS_11b-12b**

C 0.35428800 0.18589000 -0.00111700

C 1.55161000 -0.61640800 0.32888300

C 2.87404900 2.09507000 -0.33567800

C 2.03864300 -1.55860900 -0.77664700

C 2.95913100 0.85311800 -1.22066400

C 2.87475400 -0.38338100 -0.36174300

H 3.29153400 1.87416600 0.65551300

H 3.87349400 0.83746100 -1.81656200

H 3.47903300 2.90954000 -0.74614600

H 2.13163200 0.86104400 -1.94709600

H 1.51265700 -1.52362600 -1.72866500

C 0.28334800 1.65109700 0.10530200

H 0.28710100 1.53413400 1.23215900

C 1.45414700 2.64466900 -0.19876400

H 1.18291300 3.05653300 -1.18066500

C -2.10805100 0.10442500 -0.68539000

C -0.89226400 -0.63622600 -0.15560300

C -1.06404500 2.29831000 -0.28917200

C -2.27955800 1.44404700 0.04415000

H -1.08171100 -1.07260900 0.84087000

H -0.61155900 -1.49701200 -0.77046500

H -1.87387700 0.37173600 -1.72553800

H -1.13161800 3.27373500 0.19920000

H -1.03450300 2.48980000 -1.36905200

C -4.47311100 0.65723000 -0.54439000

C -3.51781900 -0.56502800 -0.73189600

C -3.59879700 1.92353200 -0.57342000

H -5.26170300 0.67946800 -1.29882900

H -4.97460000 0.59336200 0.42487300

H -3.65983500 -0.97262600 -1.74069000

H -4.04856900 2.76089000 -0.03243500

H -3.41997700 2.25002000 -1.60490000

C -3.80647000 -1.73466200 0.23478300

H -3.50303700 -1.44691400 1.25351400

C -5.30603100 -2.05955100 0.25519100

H -5.49740900 -2.92857800 0.88951500

H -5.91878700 -1.23785200 0.62878700

H -5.65049000 -2.30414000 -0.75602900

C -3.06668900 -3.01855700 -0.16290200

H -3.35521700 -3.31632000 -1.17682500

H -1.97890500 -2.93585300 -0.13925900

H -3.34026200 -3.83654900 0.50859200

C -2.43632900 1.30653100 1.56709100

H -2.52625000 2.29902600 2.01947000

H -3.33011300 0.74098100 1.83581400

H -1.59004000 0.80478400 2.05222900

C 1.45354000 3.78675600 0.82392000

H 1.72105300 3.40932500 1.81673600

H 2.20203000 4.53090500 0.54259400

H 0.49525800 4.30239900 0.89932600

C 1.41932300 -1.10969000 1.78830200

H 1.17921900 -2.17492500 1.83316800

H 2.35247900 -0.92855900 2.32353800

H 0.63716700 -0.56947000 2.32995500

C 2.70410000 -2.84981200 -0.35315700

C 3.84042500 -2.42795800 0.60771300

C 4.12170600 -0.91749000 0.36310500

H 3.10485900 -3.33175400 -1.24934600

H 2.01191200 -3.56326000 0.10618300

H 3.55835200 -2.61544200 1.64503500

H 4.74237400 -3.01675100 0.42595900

H 4.26129300 -0.39963200 1.32095200

C 5.38853400 -0.71919100 -0.47153200

H 5.63522200 0.33735900 -0.60420400

H 6.23664700 -1.19397300 0.02643300

H 5.28162700 -1.17549600 -1.46189600

**IM12b**

C -0.46176673 0.12878614 -0.63521422

C -1.56921086 -0.69173101 -0.97135929

C -2.78985374 1.99706195 -1.25224035

C -1.80415048 -0.56911023 0.68995552

C -3.63040569 0.89560158 -0.62958658

C -2.90444419 -0.40653794 -0.33284213

H -2.37487136 1.66676106 -2.21397073

H -4.44436834 0.64364402 -1.31825771

H -3.45686088 2.83347503 -1.48221444

H -4.10395072 1.26252152 0.28765996

H -1.49077252 0.29672598 1.26505161

C -0.38860944 1.63497119 -0.38777200

H 0.14665630 1.94130047 -1.30386540

C -1.66579204 2.50109253 -0.34787294

H -2.03620345 2.52018571 0.68899596

C 1.74475000 -0.16678564 0.51975428

C 0.90702005 -0.53094625 -0.70828166

C 0.53470123 1.96475165 0.81157509

C 1.92381154 1.34835681 0.68046273

H 1.38987219 -0.18603472 -1.63332296

H 0.81194945 -1.61160420 -0.78621191

H 1.12892075 -0.46394461 1.38550352

H 0.60316592 3.05070313 0.91082891

H 0.05979731 1.60423373 1.73676123

C 3.80739056 0.25106156 1.71791656

C 3.13142303 -0.81748173 0.79596396

C 2.77861032 1.37586104 1.95496348

H 4.16588435 -0.18136914 2.65409276

H 4.68514995 0.66530874 1.21321743

H 2.95663753 -1.73613807 1.37189875

H 3.25132815 2.34573330 2.13390615

H 2.14790016 1.14906716 2.82314994

C 4.03640991 -1.24130900 -0.38205463

H 4.24452187 -0.36600821 -1.01295864

C 5.36862227 -1.76555737 0.17069567

H 6.03758666 -2.04638411 -0.64638885

H 5.89076147 -1.03417381 0.79037659

H 5.19485376 -2.65932667 0.78114267

C 3.42747238 -2.34381844 -1.25870836

H 3.03604189 -3.15760130 -0.63622461

H 2.62633159 -1.99437883 -1.91187184

H 4.19538465 -2.77379128 -1.90698281

C 2.69562406 2.01525050 -0.46922624

H 2.68789497 3.10153899 -0.33358594

H 3.73946440 1.69836474 -0.49193415

H 2.27670038 1.80678132 -1.45868179

C -1.31313894 3.93873548 -0.75518613

H -1.05796918 3.97301259 -1.81996055

H -2.17252610 4.59393842 -0.59750019

H -0.47487202 4.35539915 -0.19457903

C -1.31997356 -1.99483537 -1.70524831

H -0.87027768 -2.77978612 -1.09437070

H -2.25764370 -2.37166921 -2.11293560

H -0.64332491 -1.79467137 -2.53908011

C -1.84197679 -1.92636254 1.35321727

C -2.98022070 -2.69512847 0.64047212

C -3.81315465 -1.64483970 -0.13924456

H -2.06322252 -1.76689248 2.41248990

H -0.87654702 -2.44178302 1.30924962

H -2.58325622 -3.46596026 -0.01944436

H -3.60890117 -3.20954933 1.36974903

H -4.13104917 -2.03527168 -1.11283481

C -5.06479330 -1.27125256 0.66351405

H -5.69790255 -0.54705149 0.14852159

H -5.66335962 -2.16807978 0.83596647

H -4.79370480 -0.85708709 1.64134160

**TS_12b-13b**

C -0.13999700 -0.21263300 -0.75517200

C -1.04689400 -1.20117900 -0.65661500

C -2.46019500 1.44020400 -1.77133100

C -2.37216000 -1.06919400 0.04771300

C -3.70360500 0.59596200 -1.44231200

C -3.57128500 -0.43657700 -0.39695700

H -1.79153300 0.87566800 -2.42402500

H -4.01878000 0.02366500 -2.33256400

H -2.82276100 2.28947600 -2.35621900

H -4.55978000 1.23118800 -1.18860600

H -2.37012200 -0.04295400 0.60354400

C -0.30755100 1.25810900 -0.38791200

H 0.30846000 1.77774000 -1.13535800

C -1.68338300 1.95530300 -0.53436200

H -2.28774600 1.81677300 0.37865600

C 2.03207500 -0.31144700 0.30597500

C 1.31576900 -0.52374800 -1.04538900

C 0.39022200 1.47096400 0.97610400

C 1.88218700 1.12344000 0.85728300

H 1.71273200 0.14597700 -1.81598100

H 1.46205700 -1.54812900 -1.38804500

H 1.45644200 -0.93043100 1.01352600

H 0.26819100 2.50348900 1.32061300

H -0.08483000 0.82000400 1.72606900

C 3.87411700 0.13955500 1.81531700

C 3.50807200 -0.72636900 0.56390100

C 2.62447500 0.96516400 2.19112800

H 4.22643400 -0.47042300 2.64987800

H 4.69441000 0.81834600 1.56334900

H 3.50590500 -1.78861200 0.84383900

H 2.88056000 1.92249800 2.65514400

H 1.99208100 0.41303200 2.89690300

C 4.55680400 -0.60789400 -0.56314600

H 4.62422700 0.43939600 -0.88753300

C 5.92805600 -1.03492600 -0.02327600

H 6.69459700 -0.92873900 -0.79514300

H 6.24569500 -0.45118500 0.84322500

H 5.90036800 -2.08943800 0.27532600

C 4.23929100 -1.47660500 -1.78578100

H 4.00102100 -2.50165100 -1.47705900

H 3.40965600 -1.09766900 -2.38336100

H 5.11028000 -1.53014000 -2.44469300

C 2.59728400 2.18893800 0.00609600

H 2.29809800 3.18971900 0.33582500

H 3.68106500 2.12461100 0.11500500

H 2.38088800 2.11380800 -1.06335700

C -1.46899700 3.46934800 -0.66264200

H -0.92744200 3.69516100 -1.58700200

H -2.42712100 3.99445200 -0.69584200

H -0.89393600 3.87426500 0.17226700

C -0.77915900 -2.62704100 -1.10236400

H -0.25677600 -3.21474400 -0.33923800

H -1.71449100 -3.14455300 -1.33538900

H -0.17292900 -2.64013600 -2.00808400

C -2.75062000 -2.11142900 1.12198000

C -4.29232100 -2.14986000 1.11322700

C -4.72159600 -0.80891800 0.49031900

H -2.33651600 -1.81279900 2.08892000

H -2.31697100 -3.08152800 0.88139600

H -4.63823100 -2.97575400 0.48641300

H -4.71759300 -2.29066100 2.10821800

H -5.62009700 -0.88598700 -0.13420600

C -4.95633600 0.30061300 1.53861000

H -5.11605500 1.28043400 1.08214300

H -5.84148900 0.05182400 2.12592300

H -4.10348500 0.36534900 2.22314800

**IM13b**

C -0.15263954 -0.20371775 -0.82586986

C -1.03922781 -1.21840149 -0.74590461

C -2.49905560 1.45347084 -1.80821327

C -2.36765409 -1.16444382 -0.08607886

C -3.73286146 0.63547661 -1.40998158

C -3.52473050 -0.40228207 -0.35004083

H -1.85527191 0.86539423 -2.46625711

H -4.14735689 0.10139880 -2.27536312

H -2.86072619 2.29725879 -2.40151077

H -4.52533937 1.30362071 -1.05570435

H -2.55939553 0.00865996 0.44032749

C -0.32671687 1.26236665 -0.44474629

H 0.31045341 1.78681095 -1.16997229

C -1.69229988 1.97540192 -0.59603203

H -2.28479478 1.85482788 0.32861793

C 1.99440843 -0.32029017 0.29157200

C 1.31218389 -0.50629720 -1.08128952

C 0.33907595 1.45049385 0.94082218

C 1.83283081 1.10543477 0.86062945

H 1.72341370 0.18387436 -1.82547463

H 1.47323090 -1.52002386 -1.44589152

H 1.39992136 -0.94962566 0.97466310

H 0.20284097 2.47780122 1.29537135

H -0.15846394 0.78908555 1.66717054

C 3.79571084 0.09953988 1.85548581

C 3.46219024 -0.74342214 0.57927340

C 2.53865490 0.92350193 2.21126212

H 4.12247455 -0.52614333 2.68874178

H 4.62511489 0.77915025 1.63806950

H 3.45099827 -1.81060528 0.83950740

H 2.78523795 1.87238717 2.69698881

H 1.88616637 0.36254833 2.89137450

C 4.54013832 -0.60608739 -0.51768606

H 4.62126866 0.44760442 -0.81687794

C 5.89434122 -1.05222675 0.04890681

H 6.68211900 -0.93062114 -0.69884150

H 6.19005201 -0.49089259 0.93772444

H 5.85465590 -2.11349450 0.32097271

C 4.25015815 -1.44544855 -1.76735360

H 3.99548823 -2.47515188 -1.48846380

H 3.44113325 -1.04613930 -2.37993735

H 5.13874198 -1.49195611 -2.40277843

C 2.57178688 2.18373528 0.04707393

H 2.26696909 3.17887900 0.38821079

H 3.65231720 2.11431129 0.18170336

H 2.38155919 2.12999748 -1.02863207

C -1.45368670 3.48351925 -0.74298901

H -0.92923137 3.68929738 -1.68178090

H -2.40310988 4.02460609 -0.76139999

H -0.85290559 3.88730113 0.07461733

C -0.70073580 -2.64460746 -1.15813853

H -0.11633338 -3.17286834 -0.39692951

H -1.60573046 -3.22657441 -1.35113493

H -0.12740019 -2.64086596 -2.08510355

C -2.66343651 -2.13485517 1.05970694

C -4.19373202 -2.11159334 1.21680077

C -4.64518591 -0.76596822 0.61354406

H -2.12163499 -1.80474438 1.95313414

H -2.27717184 -3.12481516 0.81604156

H -4.63038321 -2.93840633 0.65172786

H -4.50798922 -2.21877661 2.25618971

H -5.55976779 -0.86996639 0.01935420

C -4.85518925 0.33323755 1.66341673

H -5.04927990 1.30882984 1.20978860

H -5.70761706 0.08372665 2.29767601

H -3.97319708 0.42186518 2.30956220

**TS13b-PD**

C -0.15712600 -0.15432700 -0.86811600

C -1.01992300 -1.19674700 -0.83497500

C -2.53466300 1.52641200 -1.75035700

C -2.33940400 -1.21028100 -0.17703100

C -3.74254500 0.67597200 -1.34915500

C -3.48585200 -0.39829700 -0.32851400

H -1.89682800 0.97454500 -2.44521000

H -4.18310000 0.17485100 -2.22000600

H -2.92114400 2.38660500 -2.30331900

H -4.52590300 1.32189300 -0.93772800

H -2.59040400 -0.00044900 0.45062700

C -0.34929500 1.28725400 -0.41016800

H 0.29864400 1.85085200 -1.09471500

C -1.71155500 2.01216600 -0.53681700

H -2.29751700 1.87120500 0.38840200

C 1.97769400 -0.31160900 0.26726900

C 1.31485400 -0.42061300 -1.12387900

C 0.29564300 1.40040500 0.99460100

C 1.79378100 1.07542200 0.91905300

H 1.72348300 0.32227100 -1.81667400

H 1.49732500 -1.40563200 -1.55045000

H 1.38155500 -0.98698500 0.90359900

H 0.14016900 2.40500500 1.40283000

H -0.20635100 0.69470100 1.67511800

C 3.75148900 0.02722400 1.87990500

C 3.44575100 -0.73736400 0.54828200

C 2.48160400 0.81722300 2.26658800

H 4.07115100 -0.64653400 2.67763300

H 4.57784500 0.72572700 1.71829400

H 3.44281700 -1.81893800 0.74045600

H 2.71239000 1.73670200 2.81275300

H 1.82473000 0.21039400 2.90162700

C 4.53707700 -0.51978000 -0.52215400

H 4.61059000 0.55138000 -0.75324100

C 5.88814300 -0.98686900 0.03503000

H 6.68463300 -0.81095800 -0.69234800

H 6.16573900 -0.47897900 0.96105000

H 5.85646300 -2.06334000 0.24012600

C 4.27356100 -1.28155200 -1.82617700

H 4.02627500 -2.32928400 -1.61633000

H 3.46903100 -0.85223900 -2.42431600

H 5.17125000 -1.27911100 -2.45033000

C 2.53394800 2.20794900 0.18412200

H 2.21302700 3.17754900 0.57957900

H 3.61285600 2.14128700 0.33228900

H 2.36146000 2.21763000 -0.89587100

C -1.45668300 3.52036000 -0.64771800

H -0.95019200 3.74562300 -1.59195500

H -2.39873500 4.07400900 -0.62969700

H -0.83179800 3.89289000 0.16718500

C -0.62922100 -2.59480600 -1.30087100

H -0.01759900 -3.12677400 -0.56414700

H -1.51037600 -3.20484200 -1.51346500

H -0.06494000 -2.53067900 -2.23110900

C -2.61104900 -2.24798600 0.90761900

C -4.13510300 -2.23369600 1.11053800

C -4.60517900 -0.85322000 0.60581400

H -2.04078400 -1.97066400 1.80240600

H -2.22908800 -3.22093500 0.59715000

H -4.58898000 -3.02614600 0.51110400

H -4.41591600 -2.40525400 2.15091400

H -5.51562900 -0.93490000 0.00252700

C -4.84106300 0.16038100 1.73072600

H -5.05200200 1.16174500 1.34532300

H -5.69064300 -0.14751000 2.34301200

H -3.96281600 0.22157200 2.38522700

**PD**

C -0.26202900 0.05115000 -0.92708700

C -1.21966400 -0.96399400 -1.15078400

C -2.90993200 1.77964300 -1.21727300

C -2.40146300 -1.04311200 -0.42494100

C -3.79204500 0.94447400 -0.29151700

C -2.97335300 -0.07039900 0.54828000

H -2.59408500 1.20502600 -2.09527400

H -4.57590900 0.42874500 -0.85648200

H -3.50805000 2.61009300 -1.60439300

H -4.29694500 1.60134700 0.42570700

H -2.20818600 0.47153300 1.10702900

C -0.39949100 1.47222600 -0.44386300

H 0.28150800 1.97178800 -1.14988800

C -1.69299800 2.34566500 -0.47444200

H -1.99690400 2.51271500 0.56784300

C 1.82150500 -0.24743600 0.26974800

C 1.18118000 -0.31498500 -1.14184800

C 0.32696100 1.61203100 0.93979700

C 1.78583600 1.16983400 0.86310400

H 1.67502200 0.39513300 -1.81369500

H 1.29528700 -1.31522000 -1.54945100

H 1.14945300 -0.83344400 0.91823400

H 0.24147100 2.66259200 1.23837400

H -0.20993600 1.01660400 1.69026200

C 3.63943500 -0.03789900 1.84568800

C 3.23324600 -0.83293600 0.55893500

C 2.46109800 0.88843800 2.21505600

H 3.91110400 -0.70082900 2.66946600

H 4.52462800 0.56840100 1.63151000

H 3.10324900 -1.89405600 0.80856800

H 2.78866100 1.79782700 2.72631300

H 1.75431400 0.37249200 2.87574400

C 4.33558600 -0.79788800 -0.52218700

H 4.54173100 0.24536400 -0.79659800

C 5.61895500 -1.40324400 0.06242600

H 6.43245100 -1.35015800 -0.66509300

H 5.95220500 -0.89791500 0.97117700

H 5.45765600 -2.45978400 0.30571600

C 3.98202700 -1.57507700 -1.79635900

H 3.57631000 -2.56330100 -1.54782800

H 3.26632200 -1.05838200 -2.43760300

H 4.88117900 -1.73695900 -2.39643200

C 2.61378700 2.20274700 0.08395300

H 2.48026300 3.19137600 0.53429000

H 3.67966900 1.97303500 0.11718400

H 2.33688700 2.28436900 -0.97206000

C -1.29825900 3.71093400 -1.05022800

H -1.08658700 3.62841800 -2.12166800

H -2.10736700 4.43303800 -0.92025100

H -0.40637900 4.11436700 -0.55963800

C -0.87658300 -2.12828400 -2.06654100

H -0.41068000 -2.95762200 -1.52350600

H -1.78100200 -2.51199300 -2.53886700

H -0.20424600 -1.82548800 -2.86879700

C -3.29809700 -2.24836200 -0.48211900

C -4.43138700 -1.99034000 0.52457500

C -3.85199200 -0.93108100 1.47891800

H -2.69841800 -3.13149400 -0.21857900

H -3.64656900 -2.43221700 -1.50506200

H -5.31486800 -1.60756700 0.00759300

H -4.73026700 -2.90038900 1.04813800

H -4.64255800 -0.31753100 1.92197000

C -3.01283100 -1.56128700 2.59000300

H -2.55505500 -0.79494700 3.22203100

H -3.62701300 -2.20061800 3.22807900

H -2.20530400 -2.17927600 2.17816200

**TS_8d-9d-F**

C -0.81677875 1.44526450 -0.38989957

C -2.15267938 1.20391760 -1.08248384

C -3.08506429 0.64649687 1.84846004

C -2.86888826 0.08128605 -0.93808064

C -2.93049286 -0.81994876 1.45575936

C -2.50120526 -1.06867536 -0.01668704

H -3.97169387 1.10115644 1.38804831

H -3.90029943 -1.29275633 1.63977273

H -3.27496751 0.73624262 2.93573656

H -2.21880381 -1.31464652 2.12550467

H -1.42150141 -1.25341437 -0.03925733

C -0.66192116 1.11492199 1.19821412

H -0.63999119 2.52347431 -0.41730463

C -1.95432340 1.57225068 1.63652270

H -0.57431515 0.03189067 1.30229038

C 1.58292613 0.54456725 -0.21640718

C 0.35514435 0.72228874 -1.11025128

C 0.60824249 1.82198589 1.72426144

C 1.78861631 1.75013427 0.72588895

H 0.58108454 1.33188989 -1.99442019

H -0.00686630 -0.24639604 -1.46828185

H 1.36356327 -0.30476931 0.44907019

H 0.39485142 2.86496960 1.98014976

H 0.87963632 1.30974141 2.65478907

C 3.97143383 0.87147407 0.19473589

C 2.98457509 0.22079790 -0.82345224

C 3.12682127 1.37002066 1.37799805

H 4.75142286 0.17393602 0.51032012

H 4.48665084 1.71966441 -0.26287505

H 3.11305225 -0.86776212 -0.78221076

H 3.59452196 2.20265122 1.91276859

H 2.95758489 0.55916289 2.09485441

C 3.23272571 0.61012630 -2.29706137

H 2.86409912 1.63258331 -2.47039090

C 4.72612454 0.57434956 -2.64351550

H 4.87027148 0.73900808 -3.71457578

H 5.30918284 1.33000971 -2.11435378

H 5.14704281 -0.40811694 -2.40024841

C 2.51240959 -0.34826223 -3.25340820

H 2.93965323 -1.35282158 -3.15630318

H 1.44016092 -0.42949768 -3.06832119

H 2.64780149 -0.03244110 -4.29138200

C 1.96757400 3.08407263 -0.02017357

H 2.12401901 3.89227553 0.70195008

H 2.84153766 3.05942147 -0.67428151

H 1.11392788 3.36004295 -0.64506461

C -2.19682869 3.00544490 1.90736047

H -3.25484654 3.26584009 1.88410004

H -1.81528983 3.19664840 2.92283973

H -1.61756391 3.65320793 1.24451502

C -2.53583322 2.30257529 -2.04074869

H -2.74938566 3.23731681 -1.50728136

H -1.71319858 2.51659031 -2.73284623

H -3.41367849 2.04368605 -2.63284501

C -4.14848934 -0.26835289 -1.68329168

C -4.56783794 -1.63460498 -1.10962071

C -3.25031210 -2.27037361 -0.63029855

H -3.94642584 -0.33842210 -2.75828548

H -4.92609641 0.49223936 -1.56172357

H -5.24836764 -1.49154472 -0.26286533

H -5.08978319 -2.25827691 -1.83877140

H -3.43237278 -3.04446016 0.12496203

C -2.45192218 -2.87247696 -1.78536086

H -2.23075920 -2.11753704 -2.54827061

H -1.49674535 -3.27378918 -1.43193002

H -3.00380641 -3.68417407 -2.26529036

C 1.06404905 -3.24734801 0.55479554

C 0.08875544 -2.89392168 1.48699775

C 0.44853152 -2.20678203 2.64747097

C 1.78388735 -1.87586602 2.87551124

C 2.76013049 -2.24277285 1.94988442

C 2.40036424 -2.93069641 0.79227158

H 0.78849316 -3.79749906 -0.33940996

H -0.94584245 -3.18547530 1.32179392

H -0.30247283 -1.96339890 3.39469604

H 2.06849789 -1.35925148 3.78735306

H 3.80255933 -2.00401634 2.13798856

H 3.16305693 -3.23168059 0.08105815

**TS_8d-9d-R**

C -0.81586972 1.96175199 -1.14272902

C -2.13742460 1.57554420 -1.28814786

C -2.73890339 0.63935831 1.66144443

C -2.56285259 0.24194079 -1.20546292

C -2.60253504 -0.74077877 1.01481926

C -1.94378912 -0.84551402 -0.42088144

H -3.39185558 1.27607912 1.04478673

H -3.60297829 -1.17759499 0.94478499

H -3.29345856 0.51180400 2.60059726

H -2.00913215 -1.40942592 1.64614014

H -0.85823240 -0.77036899 -0.31821092

C -0.27320333 0.81338250 1.94143203

H -0.68783554 3.03969234 -1.07267184

C -1.47167689 1.41077721 1.95616002

H -0.24978991 -0.25409514 1.72418000

C 1.47642077 0.81197300 -0.32742946

C 0.44936179 1.22346242 -1.43114170

C 1.08986247 1.41459262 2.15478786

C 1.76437817 1.84957071 0.82534876

H 0.96780569 1.91233300 -2.11404975

H 0.21187216 0.32816131 -2.01157673

H 1.07034327 -0.09917409 0.11945980

H 1.05998521 2.27064168 2.83759246

H 1.71507964 0.65300871 2.63287604

C 3.77852465 1.61277053 -0.50280151

C 2.87829534 0.45214264 -0.93721550

C 3.30735110 1.84936648 0.92789136

H 4.84094160 1.36841155 -0.56715523

H 3.60542744 2.49872734 -1.12945796

H 3.22489118 -0.42453936 -0.36469210

H 3.69078811 2.76737494 1.38075802

H 3.64186382 1.01113307 1.55216337

C 2.95089866 0.04133002 -2.41518460

H 2.42367536 0.78590252 -3.02707678

C 4.39946639 -0.00202505 -2.90899713

H 4.44350945 -0.39064580 -3.92995795

H 4.86178449 0.98760820 -2.90699589

H 5.00281208 -0.66129791 -2.27468622

C 2.30175279 -1.32922139 -2.63325988

H 2.94053488 -2.11275257 -2.20912810

H 1.32195704 -1.41089408 -2.14981144

H 2.17549046 -1.54858532 -3.69688435

C 1.31791213 3.28333628 0.50188223

H 1.78888217 3.97662538 1.20462189

H 1.59955587 3.60156198 -0.50790794

H 0.23954773 3.40528487 0.62468343

C -1.71718954 2.85188887 2.31658949

H -2.11244190 3.40867927 1.45615628

H -2.47687359 2.91847799 3.10304641

H -0.81971818 3.36093670 2.66921543

C -3.18199594 2.63727274 -1.58644980

H -2.76048861 3.63789308 -1.48142724

H -3.54795648 2.54013231 -2.61361833

H -4.04370667 2.55889473 -0.91760852

C -3.85208489 -0.24343912 -1.77959406

C -3.86493639 -1.77282206 -1.57180527

C -2.42712989 -2.13136046 -1.12122085

H -3.89391554 0.05752671 -2.83536224

H -4.69783877 0.27648889 -1.30640943

H -4.60100065 -2.04925683 -0.81460433

H -4.13837979 -2.29964628 -2.48807676

H -2.43449725 -2.96237659 -0.40845718

C -1.52912956 -2.47801526 -2.30610103

H -1.49617915 -1.65567996 -3.03327930

H -0.50364006 -2.67220362 -1.97797231

H -1.89211145 -3.36663571 -2.82800615

C 0.23104820 -3.36802848 0.93549506

C -0.43813892 -3.41735952 2.15863586

C 0.10954539 -2.79433968 3.27939479

C 1.33270350 -2.13338267 3.18021074

C 2.00343154 -2.08730255 1.95912628

C 1.45134178 -2.70062134 0.83490949

H -0.17871909 -3.88313219 0.07125209

H -1.37695738 -3.95726162 2.24295383

H -0.40646168 -2.84012928 4.23277971

H 1.76768895 -1.66430293 4.05717721

H 2.96577584 -1.58723415 1.88695050

H 1.98430335 -2.68059861 -0.11141580

**TS_8d-9d_TS**

C -0.81812600 1.95314800 -1.23159200

C -2.15590300 1.60570500 -1.28087200

C -2.77472600 0.75565500 1.73855500

C -2.62330000 0.29085400 -1.12508700

C -2.67152200 -0.64018100 1.11934500

C -2.02762200 -0.79265900 -0.31938700

H -3.41608100 1.39260900 1.10948400

H -3.68091200 -1.05844300 1.06673000

H -3.33052000 0.65862400 2.68069300

H -2.08517500 -1.30829900 1.75776200

H -0.93937500 -0.74451300 -0.22840500

C -0.30430500 0.88759400 1.98338700

H -0.65948400 3.02745800 -1.29323100

C -1.49025200 1.50648100 2.01222500

H -0.30643500 -0.18082800 1.77765200

C 1.43127500 0.78630700 -0.30850000

C 0.42590000 1.15730900 -1.45247800

C 1.07642600 1.46161900 2.15335900

C 1.71059200 1.87486700 0.79894400

H 0.97999900 1.77633800 -2.17115400

H 0.16690200 0.22910500 -1.96957600

H 1.02123000 -0.10977100 0.16840000

H 1.08741700 2.32193000 2.83162000

H 1.70374500 0.68851400 2.61109200

C 3.70846100 1.62179700 -0.55590100

C 2.83690000 0.40850400 -0.88900500

C 3.25574200 1.93558200 0.86733100

H 4.77691700 1.40649200 -0.62474000

H 3.49617700 2.45932100 -1.23536000

H 3.20041100 -0.40297400 -0.24010200

H 3.61332900 2.89495700 1.25017000

H 3.63282700 1.15247500 1.53697000

C 2.90954500 -0.13475100 -2.32233600

H 2.39436200 0.55648300 -3.00313800

C 4.35965200 -0.23856500 -2.80180200

H 4.40578000 -0.72281400 -3.78098800

H 4.83210600 0.74237600 -2.89063300

H 4.95304800 -0.84021200 -2.10369200

C 2.24202800 -1.51131900 -2.41708700

H 2.87427300 -2.26580300 -1.93522700

H 1.26955000 -1.54163600 -1.91319400

H 2.10066600 -1.81555200 -3.45781400

C 1.20787700 3.27929700 0.43977300

H 1.62950500 4.00546800 1.14090000

H 1.50417400 3.58962100 -0.56858900

H 0.12161600 3.35289400 0.53181700

C -1.70339700 2.95589800 2.35815800

H -2.10606500 3.50779300 1.49816500

H -2.44401100 3.04732100 3.16018500

H -0.78861200 3.45259800 2.68307600

C -3.17647700 2.68283900 -1.60587000

H -2.72128100 3.67319200 -1.56233900

H -3.57844000 2.54779000 -2.61511600

H -4.01825800 2.66608500 -0.90794800

C -3.93480500 -0.16939500 -1.66787400

C -3.98355300 -1.69403600 -1.43717600

C -2.55444800 -2.08203000 -0.98304700

H -3.99342700 0.11931500 -2.72597600

H -4.75471100 0.38041400 -1.18228400

H -4.72522100 -1.94229800 -0.67568900

H -4.27037500 -2.22712700 -2.34568600

H -2.58757400 -2.89053800 -0.24690600

C -1.66711600 -2.49525100 -2.15368800

H -1.59715300 -1.69501700 -2.90259000

H -0.65387900 -2.72189700 -1.80911700

H -2.06348800 -3.38252000 -2.65304000

C 1.10670600 -3.32362300 0.52309100

C -0.03978000 -3.43852900 1.30886700

C -0.06839500 -2.88074700 2.58744500

C 1.04400200 -2.19661900 3.07569100

C 2.19173300 -2.08633100 2.29191700

C 2.22509400 -2.65677100 1.02045100

H 1.14533900 -3.78715300 -0.45857600

H -0.89359400 -4.00073800 0.94185100

H -0.94862900 -2.99810700 3.21276500

H 1.02221400 -1.76545900 4.07165000

H 3.06931500 -1.57898400 2.68201200

H 3.13257500 -2.60437900 0.42708900

TS_9d-10d-F

C -0.02540500 -0.88172600 1.37731400

C 0.99299800 -2.00928800 1.38858600

C 2.83632800 0.46626200 1.67447300

C 1.99055200 -2.09572400 0.50608300

C 3.39572300 -0.07040800 0.35662100

C 2.41607200 -0.97528500 -0.40984100

H 2.69240900 -0.34506500 2.39201900

H 4.31269000 -0.62889500 0.57416400

H 3.57745200 1.13729400 2.11631300

H 3.68321100 0.76926500 -0.28664900

H 1.56203500 -0.37344600 -0.75840600

C 0.23931300 0.56341800 1.50133500

H -0.58969900 -0.95656100 2.33235700

C 1.53372200 1.27116100 1.51807600

H 1.46550200 1.62555200 0.45302400

C -1.99399800 -0.01440000 -0.06314200

C -1.12185800 -1.21004700 0.27597500

C -0.98045100 1.41687600 1.65195900

C -2.31183100 0.80377500 1.20328800

H -1.69874700 -2.06055900 0.65022800

H -0.55477000 -1.55100100 -0.59393700

H -1.36009900 0.65565300 -0.66526700

H -0.98631100 1.70615600 2.71872600

H -0.78943900 2.35147200 1.10949500

C -4.21524300 0.98819500 -0.25682200

C -3.33531800 -0.15540300 -0.84906400

C -3.29863700 1.83724400 0.63893400

H -4.69914900 1.58213500 -1.03483400

H -5.01953400 0.56623100 0.35164200

H -3.12605100 0.07338900 -1.90256900

H -3.84687000 2.38144700 1.41358400

H -2.74932900 2.57439700 0.03948300

C -4.01948500 -1.53780200 -0.84776700

H -4.06074700 -1.91428500 0.18614500

C -5.45341600 -1.44365700 -1.38299000

H -5.89635600 -2.44013000 -1.45581700

H -6.10832100 -0.83807100 -0.75401100

H -5.45267200 -1.00733900 -2.38842100

C -3.26102100 -2.54522800 -1.71982900

H -3.27953400 -2.21546600 -2.76446700

H -2.21582700 -2.67734300 -1.43509900

H -3.74086100 -3.52645700 -1.67705500

C -3.00979400 0.00279600 2.31566100

H -3.17222100 0.64131800 3.18940100

H -3.98849500 -0.34811100 1.98341100

H -2.46384500 -0.88366000 2.65065600

C 1.56746000 2.55506100 2.37172000

H 2.48965200 3.08920100 2.13990700

H 0.73995900 3.23719300 2.18238500

H 1.58296700 2.29469100 3.43432900

C 0.64139600 -3.11465100 2.35180800

H 0.78256400 -2.79126700 3.39001800

H -0.40803900 -3.41776000 2.25068100

H 1.25967200 -3.99834200 2.19258300

C 2.90317000 -3.28926300 0.27771300

C 3.81819700 -2.87600100 -0.89717100

C 3.08745800 -1.70507900 -1.58777800

H 2.30231700 -4.16950900 0.02390100

H 3.47674600 -3.55129400 1.17225700

H 4.79257500 -2.54987800 -0.52330700

H 4.00374300 -3.70198400 -1.58775500

H 3.79701400 -1.04710900 -2.10363600

C 2.03916100 -2.20282300 -2.58155900

H 1.32016500 -2.86850700 -2.08826500

H 1.47823400 -1.36843200 -3.01646200

H 2.50129200 -2.76101400 -3.39933400

C 0.68066800 1.98067200 -2.26694400

C 2.06530600 2.09427600 -2.14629100

C 2.61731500 3.12641400 -1.38599200

C 1.78483800 4.04235500 -0.74205000

C 0.39928000 3.93190600 -0.86568300

C -0.15095200 2.90475700 -1.63313400

H 0.25016500 1.18972400 -2.87462700

H 2.71358200 1.38500100 -2.65357100

H 3.69559700 3.22573400 -1.30764000

H 2.21488500 4.85405000 -0.16371800

H -0.24897600 4.66263000 -0.39034100

H -1.22781600 2.83863400 -1.76245300

**TS_9d-10d-R**

C 0.27820500 1.24029800 0.96059000

C -0.67935400 2.36255500 0.57066300

C -2.27421300 0.38414500 2.42810800

C -1.92905400 2.26956400 0.09628900

C -3.36089600 0.59784700 1.29167000

C -2.77306300 1.01326000 -0.06404300

H -1.79617000 1.34160500 2.63244100

H -4.01178000 1.38425600 1.68594600

H -2.78739300 -0.02057400 3.30359200

H -3.95821100 -0.31370100 1.18917000

H -2.21101600 0.17343100 -0.48720800

C -0.17421200 -0.22825100 1.07553600

H 0.64427500 1.50646400 1.96260800

C -1.38185000 -0.58622300 1.81723100

H -0.32167300 -0.68429400 0.08304000

C 2.24124400 -0.01164900 -0.14726400

C 1.49579900 1.31415700 -0.02511400

C 0.98024700 -1.12014200 1.72448400

C 2.37809000 -0.69831400 1.22253600

H 2.16776900 2.10855900 0.31375600

H 1.11140200 1.61672200 -1.00486400

H 1.59855600 -0.68535500 -0.73495100

H 0.93068300 -1.04191900 2.81712900

H 0.78292500 -2.16073000 1.45062400

C 4.34976800 -1.25118000 -0.03697900

C 3.64540100 -0.10798100 -0.83060600

C 3.27558400 -1.89154200 0.85402400

H 4.83219700 -1.97655400 -0.69566300

H 5.13863500 -0.83756500 0.59748700

H 3.48750600 -0.44711000 -1.86300400

H 3.69270600 -2.40584300 1.72513900

H 2.68739500 -2.62241600 0.28337600

C 4.49212000 1.17729600 -0.94451300

H 4.52642600 1.67385200 0.03698800

C 5.92893800 0.84405700 -1.36577600

H 6.50369200 1.76199400 -1.51302600

H 6.46168100 0.23603100 -0.63241500

H 5.92567200 0.29924500 -2.31696500

C 3.92046100 2.15521300 -1.97763600

H 3.90891600 1.68450400 -2.96713700

H 2.90540500 2.48622500 -1.75521800

H 4.54966600 3.04669800 -2.04620600

C 3.08784200 0.17473900 2.26924000

H 3.22839600 -0.39587900 3.19298200

H 4.07407200 0.49165500 1.92491800

H 2.52890800 1.07918800 2.52122700

C -1.75782600 -2.00919800 1.91407000

H -2.83213200 -2.13600700 2.05990000

H -1.39138700 -2.61250300 1.08164000

H -1.27077900 -2.37624600 2.83356000

C -0.01461300 3.71915800 0.68836500

H 0.68604200 3.74480200 1.52906100

H 0.55956200 3.95879400 -0.21417900

H -0.74109500 4.51883400 0.83323700

C -2.76632700 3.46607200 -0.34672200

C -4.15477900 2.89352400 -0.67768600

C -3.88516600 1.42667400 -1.05136600

H -2.31139700 3.93174300 -1.22875700

H -2.81923800 4.24495400 0.41955900

H -4.80863700 2.95145400 0.19860300

H -4.65407200 3.43740300 -1.48281200

H -4.77963200 0.80604400 -0.91774400

C -3.39237200 1.29866500 -2.49312400

H -2.47518700 1.87840800 -2.64508200

H -3.17313600 0.25747500 -2.75136700

H -4.14300100 1.66592700 -3.19689400

C -1.33535000 -2.15332500 -1.73643100

C -2.65396000 -2.51277500 -1.45392600

C -2.92878800 -3.74751200 -0.86666200

C -1.88570100 -4.61578600 -0.54690200

C -0.56564600 -4.25076200 -0.81344900

C -0.29015500 -3.02128800 -1.41308800

H -1.12283100 -1.20784500 -2.23130500

H -3.46881300 -1.84293900 -1.71565300

H -3.95562700 -4.03864200 -0.67030600

H -2.10098500 -5.58095000 -0.10032400

H 0.24499700 -4.93257100 -0.57672800

H 0.73447600 -2.75438500 -1.65731500

**TS_9d-10d_TS**

C -0.33301600 -1.02629500 1.47238400

C 0.66329800 -2.17877200 1.38078500

C 2.60632200 0.11566000 2.00953700

C 1.68673100 -2.19383400 0.52139400

C 3.06384100 -0.09781700 0.54867300

C 2.12550300 -0.99292800 -0.28497000

H 2.46425800 -0.85464600 2.48882000

H 4.05575200 -0.55690400 0.60501000

H 3.38700000 0.66206000 2.54298300

H 3.18648900 0.87135100 0.04972000

H 1.27088400 -0.40072500 -0.63688000

C 0.06044000 0.43979500 1.74242500

H -0.92462100 -1.21782500 2.37687600

C 1.34323700 0.91609000 2.11319600

H 0.65915500 0.93501700 0.79280300

C -2.02755400 0.18830700 -0.02499400

C -1.30994400 -1.12651300 0.25512800

C -1.16453400 1.33663000 2.01238000

C -2.44710600 0.88287300 1.28346200

H -2.01762600 -1.93584200 0.46477700

H -0.72437300 -1.43560000 -0.61506400

H -1.26762900 0.85928000 -0.46005200

H -1.31153200 1.36829900 3.09975400

H -0.93947300 2.35732700 1.69369000

C -4.13797800 1.38636700 -0.35410300

C -3.25390900 0.27259900 -0.99166100

C -3.28226700 2.05043200 0.73261700

H -4.50112200 2.10162900 -1.09503300

H -5.02451700 0.94311100 0.10809400

H -2.88626200 0.63344400 -1.96218700

H -3.87846600 2.55676400 1.49772000

H -2.61423200 2.79793200 0.28388100

C -4.02515500 -1.02814800 -1.29685400

H -4.25202300 -1.53905500 -0.34860700

C -5.34938600 -0.72036200 -2.00678400

H -5.85649300 -1.64839700 -2.28276000

H -6.03930400 -0.13745300 -1.39403000

H -5.16069900 -0.15899400 -2.92918500

C -3.22329600 -1.97530600 -2.19679700

H -3.02265400 -1.48983200 -3.15864400

H -2.26630900 -2.28126900 -1.77248100

H -3.79709400 -2.88299700 -2.40183900

C -3.33705300 0.01200400 2.18504500

H -3.65507700 0.58995000 3.05854100

H -4.23782000 -0.31194200 1.65991400

H -2.84172300 -0.89016500 2.55109200

C 1.55598500 2.31287000 2.60144600

H 2.44981300 2.73541300 2.13576900

H 0.71379500 2.98819900 2.48509300

H 1.76832800 2.21193800 3.67495100

C 0.27293500 -3.37789600 2.20592100

H 0.33610300 -3.15442000 3.27735600

H -0.76414300 -3.67142900 2.00431400

H 0.91101900 -4.23834400 2.00283600

C 2.60173800 -3.36567300 0.20211500

C 3.57716800 -2.82741200 -0.86706000

C 2.85823000 -1.61396600 -1.49147500

H 2.01024800 -4.20214500 -0.18648800

H 3.12732000 -3.74133100 1.08569100

H 4.51678100 -2.51640400 -0.40043700

H 3.83075800 -3.57992900 -1.61739100

H 3.57243300 -0.90310000 -1.92382300

C 1.85374000 -2.04585800 -2.55891100

H 1.13620300 -2.76674800 -2.14935000

H 1.28257000 -1.19048300 -2.93608200

H 2.35562600 -2.51961700 -3.40609600

C 0.91455100 1.91113700 -1.96777600

C 2.18996300 1.75720300 -2.51041000

C 3.24352500 2.54503800 -2.04925800

C 3.02507500 3.48837800 -1.04500800

C 1.75006600 3.64658100 -0.50463400

C 0.69395200 2.85613800 -0.96403100

H 0.08762600 1.31481700 -2.34631000

H 2.35803600 1.03802300 -3.30615400

H 4.23228100 2.43452200 -2.48258700

H 3.84196900 4.11520400 -0.70237200

H 1.57031800 4.41139300 0.24533700

H -0.31218500 3.01599700 -0.58185800

**TS_10d-11d-F**

C -0.37599500 -1.36710800 0.90663600

C 0.30989300 -2.49823300 0.40452700

C 2.48867700 -1.03408600 2.26752200

C 1.55850300 -2.39677100 -0.18844400

C 3.32627600 -1.22946300 1.00461600

C 2.46147700 -1.21386700 -0.28169200

H 1.93596900 -1.94423100 2.52627800

H 3.91450000 -2.15160800 1.06131700

H 3.16761300 -0.84980300 3.10580400

H 4.03813900 -0.40115900 0.90584400

H 1.92786000 -0.26206000 -0.33319600

C 0.12732200 -0.08876600 1.51517000

H -0.56449300 -0.00729500 2.36890700

C 1.54207400 0.16357500 2.12190400

H 2.03907800 0.88672000 1.46353700

C -2.17887600 -0.23519000 -0.23483400

C -1.87435600 -1.37010300 0.77966200

C -0.27039400 1.11513100 0.58948600

C -1.76541900 1.14659300 0.29550600

H -2.35807200 -1.15672500 1.73867700

H -2.25124100 -2.31621800 0.40123100

H -1.49802300 -0.41702200 -1.08291200

H 0.05992600 2.02523300 1.10009000

H 0.30454000 1.05929200 -0.34432800

C -3.55330900 1.43282300 -1.31242500

C -3.57733700 -0.07729900 -0.89764700

C -2.17673700 2.00485700 -0.91141100

H -3.75993400 1.56972800 -2.37580300

H -4.34046900 1.96797900 -0.77263800

H -3.58628300 -0.70533400 -1.79812700

H -2.21600700 3.07362000 -0.68024400

H -1.44531000 1.86832800 -1.71731100

C -4.86149100 -0.45063800 -0.12527900

H -4.93694600 0.17210700 0.77612900

C -6.07584800 -0.15506700 -1.01571700

H -7.00408600 -0.36593400 -0.47917000

H -6.11413100 0.88336500 -1.35128200

H -6.05253200 -0.79475600 -1.90555800

C -4.92811500 -1.92390900 0.29582700

H -4.65690600 -2.57920900 -0.54084700

H -4.28606000 -2.16306800 1.14493200

H -5.94848500 -2.18228900 0.59076000

C -2.54168900 1.60437300 1.53888900

H -2.15817100 2.57571000 1.86684800

H -3.60591200 1.72736300 1.33249100

H -2.45601200 0.91722700 2.38702500

C 1.33581100 0.86407800 3.46959100

H 2.28427200 1.24941800 3.85167600

H 0.64312100 1.70788000 3.38109200

H 0.92939100 0.16850200 4.21180600

C -0.40997000 -3.83419100 0.31727300

H -1.15955200 -3.94072600 1.10104200

H -0.90330200 -3.97598100 -0.65040200

H 0.30276100 -4.64958100 0.44541500

C 2.18486100 -3.54065700 -0.93860600

C 3.48799500 -2.99330800 -1.54628700

C 3.27721600 -1.46875700 -1.56502200

H 1.47584200 -3.88069400 -1.70634900

H 2.33247600 -4.40455800 -0.27962200

H 4.34097100 -3.26270700 -0.91869300

H 3.67734100 -3.39707800 -2.54277700

H 4.23047800 -0.93159100 -1.51912800

C 2.49297800 -1.02462600 -2.79973000

H 1.55040400 -1.57853800 -2.89073700

H 2.24271100 0.03881400 -2.74431400

H 3.06757300 -1.19622200 -3.71280200

C 1.90119800 2.81240500 -1.94831200

C 2.95613300 2.35952900 -1.15622200

C 3.12070100 2.85739200 0.13636900

C 2.22874200 3.80407600 0.63862400

C 1.17427800 4.25599900 -0.15374400

C 1.00999500 3.76000400 -1.44602600

H 1.78726600 2.44809900 -2.96516000

H 3.66921300 1.64328100 -1.55660200

H 3.95778100 2.52621400 0.74459000

H 2.36616600 4.20153900 1.63946400

H 0.48921000 5.00524400 0.23060200

H 0.19975600 4.12595900 -2.06878200

**TS_10d-11d-R**

C -0.08127100 -1.32266900 1.17156400

C 0.86037000 -2.43585400 0.71041500

C 2.94177600 -0.45287200 1.91890400

C 1.86763000 -2.22286300 -0.14183700

C 3.45664600 -0.41330800 0.48226400

C 2.40908700 -0.87541800 -0.54638900

H 2.76890600 -1.48387500 2.23920600

H 4.34342400 -1.05210800 0.41079600

H 3.69653100 -0.03503200 2.59298200

H 3.78052800 0.60193800 0.22437700

H 1.61331000 -0.11164700 -0.61270800

C 0.43635900 0.05292700 1.34749000

H -0.24539200 -1.50725400 2.26084700

C 1.67138400 0.37861100 2.11646500

H 1.89748400 1.42752700 1.88469900

C -1.96525400 -0.23397900 -0.20541300

C -1.46752300 -1.50464200 0.46088700

C -0.37765600 1.23408900 0.98656200

C -1.85661400 0.96530100 0.75016500

H -2.19984400 -1.87438200 1.18620700

H -1.33407100 -2.28686200 -0.28788400

H -1.26010100 -0.01594800 -1.02482200

H -0.17502000 2.05138900 1.69153700

H 0.10962400 1.58077500 0.04761600

C -3.83654900 1.30861700 -0.54833600

C -3.39690900 -0.15866400 -0.83661200

C -2.58684500 2.04716200 -0.05136200

H -4.28427200 1.78585500 -1.42240800

H -4.59839100 1.32126600 0.23757200

H -3.28191100 -0.28454900 -1.92087800

H -2.81677900 2.94326300 0.53339900

H -1.95832600 2.34942700 -0.89652600

C -4.45780900 -1.19509300 -0.40739100

H -4.52592400 -1.20618100 0.69132500

C -5.83100000 -0.80218700 -0.96830300

H -6.58043500 -1.54980100 -0.69700700

H -6.18209200 0.16495900 -0.60397700

H -5.79007500 -0.75550000 -2.06269700

C -4.14376500 -2.61245700 -0.90098800

H -4.01366000 -2.61082600 -1.98899300

H -3.24894300 -3.04849900 -0.45628100

H -4.97608600 -3.28340000 -0.67254300

C -2.54181400 0.73377900 2.10528900

H -2.52305600 1.65477000 2.69694500

H -3.58537400 0.43503700 1.98883300

H -2.04783100 -0.04629800 2.69646800

C 1.21498800 0.33147100 3.60791700

H 2.00262200 0.78776600 4.21010600

H 0.28637500 0.87992900 3.78694500

H 1.08801900 -0.70479100 3.93185400

C 0.45187300 -3.80471800 1.18720400

H 0.67689900 -3.92188300 2.25411500

H -0.62259100 -3.97764900 1.06579400

H 0.98238200 -4.59037300 0.64908500

C 2.68341900 -3.30001900 -0.84138900

C 3.63009000 -2.53516900 -1.79421700

C 3.01274600 -1.12854100 -1.94076700

H 2.01546400 -3.96968900 -1.39335900

H 3.23517000 -3.92436000 -0.13160100

H 4.63185100 -2.46813800 -1.36137600

H 3.73518500 -3.03292200 -2.76093900

H 3.78460800 -0.38579900 -2.17508900

C 1.92429800 -1.09266900 -3.01188900

H 1.14075800 -1.82828500 -2.79470000

H 1.44974400 -0.10618300 -3.05666800

H 2.33292600 -1.31850000 -3.99956100

C 1.03681300 2.89580800 -2.04244800

C 2.20732700 2.55341900 -1.36368500

C 2.51597300 3.17379400 -0.15193400

C 1.65015200 4.12490700 0.38775300

C 0.48073000 4.46638900 -0.29021400

C 0.17960200 3.85868000 -1.50831200

H 0.80859900 2.43722300 -2.99970100

H 2.89222100 1.82845500 -1.79594800

H 3.44728200 2.94159100 0.35727200

H 1.90039700 4.61903100 1.32143600

H -0.18365100 5.22121700 0.11780300

H -0.71601800 4.14631900 -2.04988400

**TS_10d-11d_TS**

C 0.14362400 -1.13117800 0.76777500

C 1.14978200 -2.20131100 0.66338500

C 2.92445200 -0.00641900 2.05611700

C 2.28323100 -1.93004200 -0.01160700

C 3.57066600 0.08845600 0.67851800

C 2.74917400 -0.56355400 -0.45123300

H 2.87813500 -1.04679300 2.39224900

H 4.56028200 -0.37871600 0.72632200

H 3.57089200 0.51085700 2.77333000

H 3.73604600 1.14051900 0.41520600

H 1.90831200 0.09413400 -0.70282000

C 0.33581800 0.13949300 1.38564500

H -0.16571900 -0.77318600 2.07925500

C 1.54669400 0.64992200 2.18773200

H 1.64205000 1.68966400 1.84390200

C -2.02473600 -0.27577100 -0.25382400

C -1.19672900 -1.49295100 0.12490000

C -0.70659500 1.23362100 1.18212400

C -2.11401200 0.71223100 0.92055100

H -1.73573300 -2.17230600 0.79959000

H -0.93367400 -2.08921000 -0.75337300

H -1.44429700 0.25664300 -1.02335800

H -0.67636000 1.91745900 2.03620400

H -0.34273700 1.80526300 0.31565100

C -4.17546500 0.87102900 -0.30245000

C -3.46919500 -0.41709400 -0.83029200

C -3.08764900 1.74584300 0.34248400

H -4.71784600 1.39509800 -1.09194000

H -4.91658300 0.60391200 0.45657200

H -3.39481100 -0.35715000 -1.92371100

H -3.48530700 2.43236000 1.09579500

H -2.56636100 2.34263500 -0.41442100

C -4.26245200 -1.70958100 -0.53871600

H -4.25076800 -1.90442200 0.54471400

C -5.72230600 -1.54532200 -0.98164100

H -6.27752400 -2.47142200 -0.81361500

H -6.24386300 -0.74672900 -0.45148900

H -5.76575700 -1.32197900 -2.05384300

C -3.69443400 -2.93240500 -1.27015200

H -3.65525000 -2.74004900 -2.34808600

H -2.69388500 -3.22180800 -0.94577600

H -4.34313100 -3.79866900 -1.11609900

C -2.70140900 0.10187100 2.20306500

H -2.77310300 0.86991500 2.97953200

H -3.70405500 -0.29744000 2.03987000

H -2.10471500 -0.72109000 2.61714800

C 1.14221800 0.66852800 3.67562100

H 1.86402200 1.24909400 4.25295800

H 0.15269800 1.10147300 3.84354200

H 1.14214500 -0.35243500 4.07403500

C 0.71362200 -3.60263700 1.02145400

H 0.19238100 -3.62577000 1.98342200

H 0.03822000 -4.02867700 0.27086500

H 1.57933800 -4.26096900 1.09870500

C 3.26384800 -2.97316700 -0.50730000

C 4.30763700 -2.18644500 -1.32724800

C 3.60621900 -0.86366800 -1.69762100

H 2.73367400 -3.70873800 -1.12332300

H 3.71143800 -3.53148700 0.32206500

H 5.19632600 -1.99403100 -0.72052900

H 4.63734800 -2.73543400 -2.21196100

H 4.33723000 -0.06367500 -1.86313200

C 2.73017400 -1.01698300 -2.93989200

H 1.98207100 -1.80656000 -2.79933500

H 2.19391400 -0.08850600 -3.16034500

H 3.32895600 -1.27703500 -3.81585800

C 0.68384600 2.59637000 -2.18407200

C 1.84218500 2.68389100 -1.41157000

C 1.89112700 3.55422700 -0.32226700

C 0.78379100 4.34207400 -0.00844400

C -0.36919600 4.26430400 -0.78796500

C -0.41968900 3.39111000 -1.87467000

H 0.65071200 1.93532300 -3.04488000

H 2.71720500 2.09630900 -1.67830700

H 2.80188400 3.64155400 0.26357900

H 0.82755100 5.03152600 0.82846300

H -1.22473800 4.89062800 -0.55585800

H -1.31206900 3.34327200 -2.49137900

^1^H-NMR spectrum of **2**


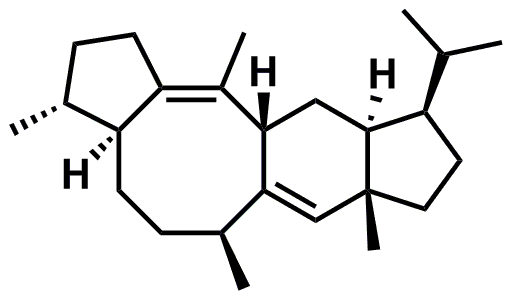


**2**

**^1^H-NMR**

^13^C-NMR spectrum of **
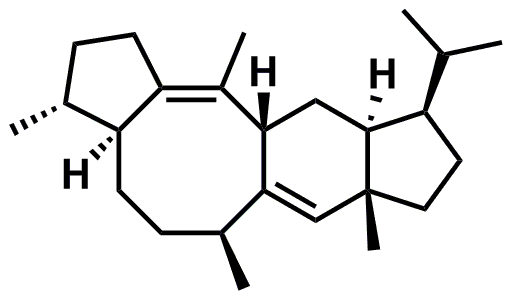
2**

**2**

**^13^C-NMR**

H-H COSY spectrum of **
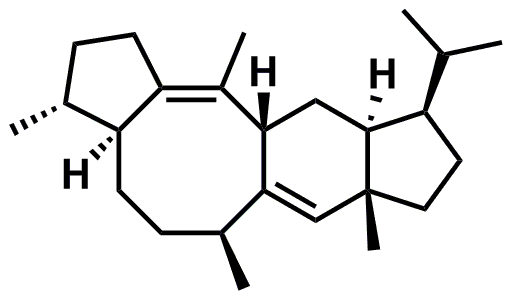
2**

**2**

**H-H COSY**

HSQC spectrum of **
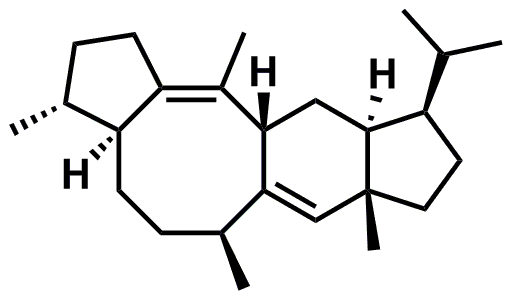
2**

**2**

**HSQC**

HMBC spectrum of **
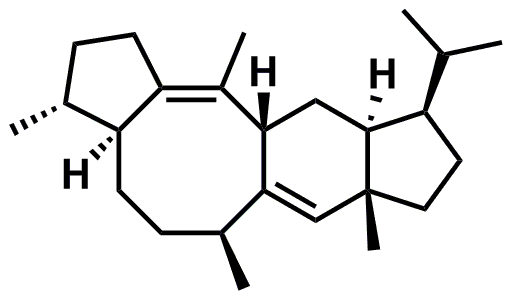
2**

**2**

**HMBC**

NOESY spectrum of **2**


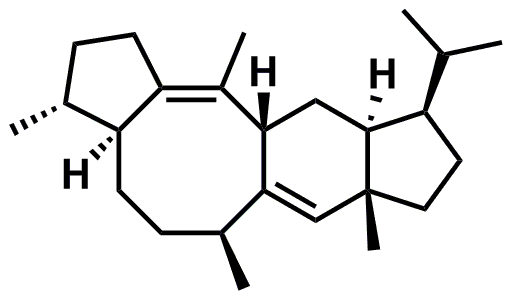


**2**

**NOESY**

^1^H-NMR spectrum of
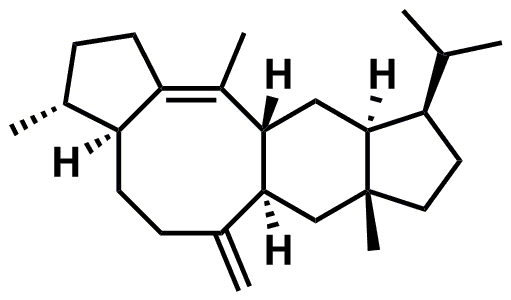
**3**

**3**

**^1^H-NMR**

^13^C-NMR spectrum of
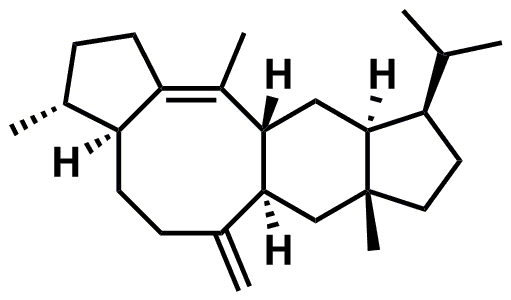
**3**

**3**

**^13^C-NMR**

H-H COSY spectrum of
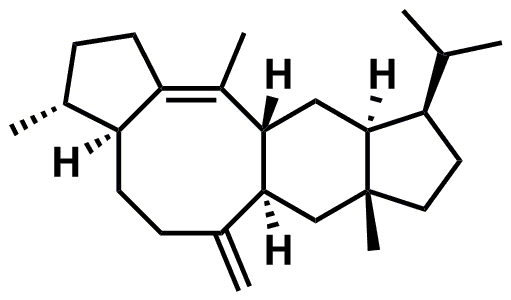
**3**

**3**

**H-H COSY**

HSQC spectrum of
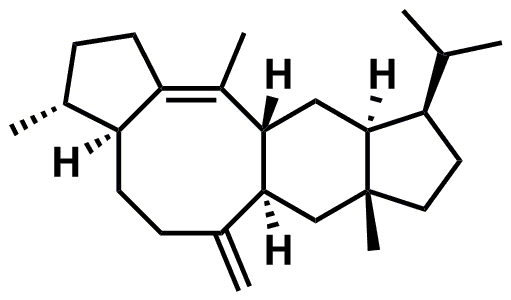
**3**

**3**

**HSQC**

**
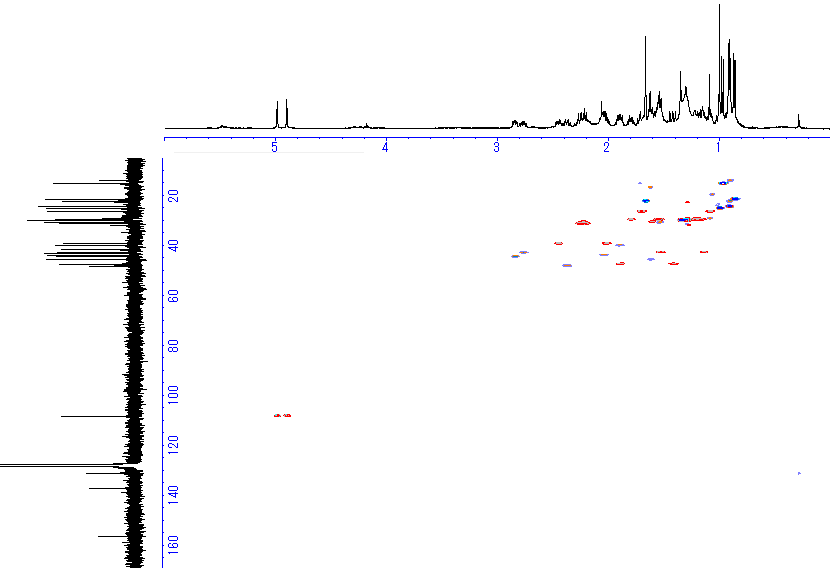
**

HMBC spectrum of
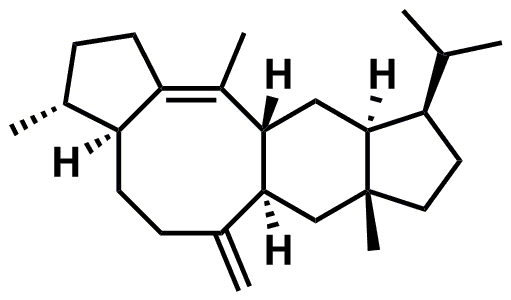
**3**

**3**

**HMBC**

**
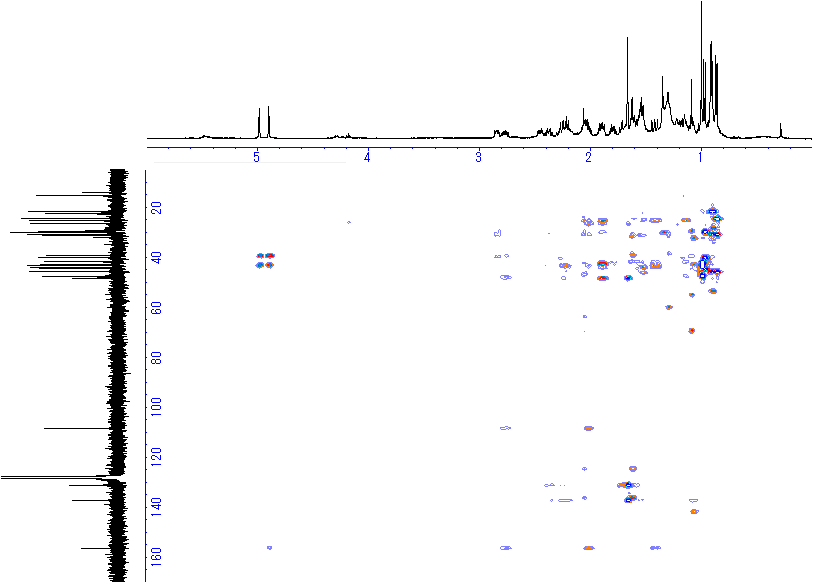
**

NOESY spectrum of
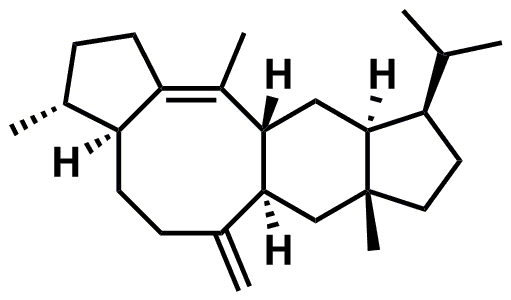
**3**

**3**

**NOESY**
